# *Lactobacillus paracasei* ATG-E1 improves particulate matter 10 plus diesel exhaust particles (PM_10_D)-induced airway inflammation by regulating immune responses

**DOI:** 10.3389/fmicb.2023.1145546

**Published:** 2023-04-27

**Authors:** Young-Sil Lee, Gun-Seok Park, Seung-Hyun Ko, Won-Kyung Yang, Hye-Jin Seo, Seung-Hyung Kim, Nara Jeong, Jihee Kang

**Affiliations:** ^1^AtoGen Co., Ltd., Daejeon, Republic of Korea; ^2^Institute of Traditional Medicine and Bioscience, Daejeon University, Daejeon, Republic of Korea

**Keywords:** probiotics, *Lactobacillus paracasei*, particulate matter, diesel exhaust particles, airway inflammation

## Abstract

Particulate matter (PM) exposure can adversely affect respiratory function. Probiotics can alleviate the inflammatory responses in respiratory diseases. We examined the protective effects of *Lactobacillus paracasei* ATG-E1 isolated from the feces of a newborn baby against airway inflammation in a PM_10_ plus diesel exhaust particle (DEP) (PM_10_D)-induced airway inflammation model. BALB/c mice were exposed to PM_10_D by intranasal injection three times at 3-day intervals for 12 days, and *L. paracasei* ATG-E1 was administered orally for 12 days. Analysis of immune cell population and expression of various inflammatory mediators and gut barrier-related genes were determined in bronchoalveolar lavage fluid (BALF), lung, peyer’s patch, and small intestine. A histological analysis of the lungs was performed. In addition, the *in vitro* safety and their safety in genomic analyses were examined. *L. paracasei* ATG-E1 was found to be safe *in vitro* and by genomic analysis. *L. paracasei* ATG-E1 suppressed neutrophil infiltration and the number of CD4^+^, CD4^+^CD69^+^, CD62L^–^CD44^+high^, CD21/35^+^B220^+^, and Gr-1^+^CD11b^+^ cells, as well as the expression of inflammatory mediators, including chemokine (C-X-C motif) ligand (CXCL)-1, macrophage inflammatory protein (MIP)-2, interleukin (IL)-17a, tumor necrosis factor (TNF)-α, and IL-6 in BALF and lungs in PM_10_D-induced airway inflammation. It protected against histopathological damage in the lungs of mice with PM_10_D-induced airway inflammation. *L. paracasei* ATG-E1 concomitantly increased the expression levels of the gut barrier function-related genes occludin, claudin-1, and IL-10 in the small intestine, with an increased number of CD4^+^ and CD4^+^CD25^+^ immune cells in the peyer’s patch. *L. paracasei* ATG-E1 suppressed immune activation and airway inflammatory responses in the airways and lungs by restoring the lung damage by PM_10_D. It also regulated intestinal immunity and ameliorated the gut barrier function in the ileum. These results indicate the potential of *L. paracasei* ATG-E1 as an protective and therapeutic agent against airway inflammation and respiratory diseases.

## Introduction

With increasing industrialization and urbanization, air pollution is steadily increasing and has become a great concern because it induces various adverse health effects and rises the risks of mortality and morbidity through damage to the lungs and respiratory system (Brauer et al., 2012; Kim et al., 2013). Particulate matter (PM) is one of air pollutant that health threats (Gehring et al., 2010; Nkhama et al., 2017). PM consists of a heterogeneous mixture of solid and liquid granular substances in the atmosphere (Kim et al., 2015), and its chemical composition varies depending on its source, including metals, ions, nitrates, sulfates, elemental and organic carbon, and polycyclic aromatic hydrocarbons (van der Valk et al., 2015; Winterbottom et al., 2018; Riediker et al., 2019). According to the diameter, PM is divided into coarse particles (diameter ≤ 10 μm, PM_10_), fine particles (diameter ≤ 2.5 μm, PM_2.5_), and ultrafine particles (diameter ≤ 0.1 μm, PM_0.1_) (Gehring et al., 2010), and the size of PM may differently affect the respiratory system (Kim et al., 2015; Riediker et al., 2019). When PM is inhaled, the body eliminates it through processes such as sneezing and coughing (Cadelis et al., 2014). PM less than 10 μm in diameter is deposited on epithelial cells and alveolar macrophages in the lung, which causes an inflammatory response and injury in the airway and lung through stimulation of oxidative stress and the immune system. This leads to cell death, bronchial fibrosis, and a decline in respiratory functions, but it has also been reported to make worsen the asthma and chronic obstructive pulmonary disease (COPD) (Leikauf et al., 2020). Therefore, despite the interest in PM and related research is growing, there is a need for novel agents to prevent and treat PM-induced inflammation and respiratory damage.

A probiotic is defined as “a live microorganism that when administrated in adequate amounts confers a health benefit on the host” (FAO/WHO, 2002). Probiotics were originally known to regulate the intestinal microbial balance, and they have been shown to improve the inflammatory response in hosts due to their immunomodulatory effects (Forsythe, 2011, 2014). *Lactobacillus rhamnosus* GG, *Lactobacillus gasseri*, *Lactobacillus reuteri, and Bifidobacterium lactis Bb12* have been shown to decrease airway inflammatory responses by regulating immune properties in lung diseases, such as asthma, allergic rhinitis, and COPD (Feleszko et al., 2007; Forsythe et al., 2007; Carvalho et al., 2020). Furthermore, it has recently been reported that *Bifidobacterium lactis* can inhibit PM-induced lung inflammation (Wang et al., 2017). Therefore, these studies suggest that probiotics can alleviate respiratory diseases by modulating immune functions. However, few studies to date have examined the effects of probiotic strains in pathological conditions caused by air pollution, especially PM and DEP, for which there have been few studies so far. In the present study, we investigated whether *L. paracasei* ATG-E1 has beneficial effects on respiratory function through regulation of the immune and inflammatory responses in the airway and lung, and we also examined the probiotics properties including their safety and functionalities in genomic analyses as potential probiotics for improving respiratory health.

## Materials and methods

### Isolation and identification of *L. paracasei* ATG-E1

*Lactobacillus paracasei* ATG-E1 was isolated from an anonymous donor in Daejeon, Republic of Korea, who kindly provided fecal samples from a newborn baby (January 2016). In brief, fecal samples were blended with NaCl (0.85% w/v) in a sterile bag and then serially diluted with NaCl (0.85%). The serially diluted fecal samples were plated on De Man Rogosa Sharpe broth (MRS, Difco Laboratories Inc., Franklin Lakes, NJ, USA) agar plates and then incubated at 37°C for 24−48 h. Colonies grown on MRS agar plates were selected for isolation and purification based on colony morphology by microscopy and catalase activity. A catalase-negative strain was identified by 16S rRNA sequencing and analyzed using the BLAST database with a sequence-matching program and was called *L. paracasei* ATG-E1. Isolated *L. paracasei* ATG-E1 was deposited in the Korean Collection for Type Cultures (KCTC 14245BP).

### Probiotic properties of *L. paracasei* ATG-E1

#### Carbohydrate fermentation patterns

The fermentation patterns of *L. paracasei* ATG-E1 were examined using an API^®^ 50 CHL test (bioMérieux, Inc., Marcy l’Etoile, France), according to the manufacturer’s protocols. Briefly, cultures of *L. paracasei* ATG-E1 were suspended in API^®^ 50 CHL medium, and then applied to cupules on an API^®^ 50CH test strip. The patterns of fermentation were observed for up 72 h at 37°C.

#### Acid and bile tolerance

An aliquot of *L. paracasei* ATG-E1 culture was inoculated into MRS broth adjusted to pH 3.0 and incubated at 37°C for 1 h. For the bile tolerance test, *L. paracasei* ATG-E1 culture was inoculated into MRS broth containing 0.3% (w/v) oxgall (Sigma-Aldrich, St. Louis, MO, USA) that was incubated at 37°C for a further 3 h. The number of viable bacterial cells was determined by MRS agar plating after 0, 1, and 3 h and expressed as log CFU/mL.

#### Bile salt hydrolase (BSH) activity

*L. paracasei* ATG-E1 was spotted onto MRS agar plates containing the 0.5% (w/v) sodium salt taurodeoxycholic acid (TDCA, Sigma-Aldrich, St. Louis, MO, USA) and incubated under anaerobic conditions at 37°C for 48 h. The presence of halos around opaque white colonies indicated BSH activity. An MRS agar plate without *L. paracasei* ATG-E1 was used as a negative control.

### Safety test assessment of *L. paracasei* ATG-E1

#### Antibiotic resistance testing

A culture of *L. paracasei* ATG-E1 was diluted in 0.9% saline to an OD_600_ of 0.8. The bacterial suspensions were spread onto counting agar plates (PCA; Difco Laboratories Inc., Franklin Lakes, NJ, USA), and E-test strips (BioMérieux, Inc., Marcy-l’Étoile, France) were placed on the agar plates. The minimal inhibitory concentration (MIC) values were compared with the breakpoint values, as suggested by the EFSA and Panel on Additives and Products or Substances used in Animal Feed [FEEDAP], 2012. The antibiotics erythromycin, gentamycin, ampicillin, tetracycline, chloramphenicol, streptomycin, ciprofloxacin, and penicillin G were used in this experiment.

#### Hemolysis

*L. paracasei* ATG-E1 was streaked on a blood agar plate containing 5% sheep blood (Asanpharm Co., Ltd., Seoul, Republic of Korea) and incubated at 37°C for 24−48 h. The ability to produce hemolysins was measured by the formation of any clear (β-hemolysis) or greenish (α-hemolysis) hemolytic zones and no such zone (γ-hemolysis) around the *Lactobacillus* colonies. Biogenic amine formation *L. paracasei* ATG-E1 was cultured in MRS broth containing the precursor amino acids tyrosine, histidine, ornithine, and lysine (Sigma-Aldrich, St. Louis, MO, USA) to identify the production of the biogenic amines such as tyramine, histamine, putrescine, and cadaverine. Production of biogenic amines was determined by the color change of the pH indicator bromocresol purple. MRS broth was used as a negative control.

### Whole-genome analysis of *L. paracasei* ATG-E1

A single colony of *L. paracasei* ATG-E1 was transferred into MRS broth in a shaking incubator at 37°C for 6 h. Genomic DNA was extracted from the culture broths after 6 h using a QIAmp DNA mini Kit (QIAGEN, Stanford, CA, USA) and the concentration was determined using a Qubit^®^ 3.0 Fluorometer (Thermo Fisher Scientific, Waltham, MA, USA), and then the DNA quality and integrity were checked using a LabChip^®^ GX Touch™ nucleic acid analyzer (PerkinElmer, Waltham, MA, USA). Whole genomes were sequenced on a MinION platform (Oxford Nanopore Technologies, Oxford, UK) with an R9.4.1-flow cell.

### Genome assembly, gene prediction, and safety assessment

Sequence reads (fastq files) were base-called using Guppy v3.4.5^1^ with the high-accuracy base-calling algorithm. Fastq files were assembled with Flye v. 2.9 (Kolmogorov et al., 2019) using the default settings. Complete genomes were annotated using the NCBI Prokaryotic Genomes Automatic Annotation Pipeline (PGAAP) (Tatusova et al., 2016) and Average Nucleotide Identity (ANI) values were obtained using the ANI Calculator tool on EzBiocloud^2^ (Yoon et al., 2017). PathogenFinder was used to determine pathogenic potential (Cosentino et al., 2013) and ResFinder was used to determine antimicrobial susceptibility (Bortolaia et al., 2020).

### Animal experiments

#### Sample preparation

*L. paracasei* ATG-E1 was incubated in MRS broth at 37°C for 16 h, and cells were then obtained by centrifugation (3,000 × g, 10 min, 4°C) and washed three times with phosphate-buffered saline (PBS; pH 7.4). The cell pellets were resuspended in a cryoprotectant solution and lyophilized using an FD8508 freeze-dryer (ilShinBioBase, Seoul, Republic of Korea). The freeze-dried *L. paracasei* ATG-E1 powder was resuspended in saline and prepared daily for the animal experiments.

#### Animals and experimental procedures

BALB/c mice (male, 6−8 weeks old) were obtained from Orient Bio Co., Ltd. (Gyeonggi-do, Republic of Korea). The mice were housed in animal facility with environmental conditions (temperature of 22 ± 2°C, a humidity of 60 ± 10%, and a 12-h light/dark cycle), with free access to food and water. This experimental protocol was approved by the Committee for Animal Welfare at Daejeon University (DJUARB2019-021) and was performed in accordance with the committee guidelines. The mice received intranasal administration of a mixture of PM_10_ (ERM CZ-120, Sigma-Aldrich, USA) and diesel exhaust particles (DEP, SRM 2975, Sigma-Aldrich, St. Louis, MO, USA) on days 4, 7, and 10 and were orally treated with *L. paracasei* ATG-E1 every other day for 12 days. PM_10_ (3 mg/mL) and DEP (0.6 mg/mL) were dissolved in 1% aluminum hydroxide gel adjuvant. The mice were divided into following groups (*n* = 8 per group): (1) 1% aluminum hydroxide gel adjuvant-treated mice (NC), (2) PM_10_ + DEP-sensitized mice (PM_10_D-CTL), (3) 4 × 10^10^ CFU of ATG-E1-treated PM_10_ + DEP-sensitized mice (PM_10_D-ATG-E1), and (4) 3 mg/kg dexamethasone-treated PM_10_ + DEP-sensitized mice (PM_10_D-Dexa, positive control). All mice were sacrificed and blood, bronchoalveolar lavage fluid (BALF), and lung and intestinal tissues were harvested on day 12.

#### BALF collection and cytological analysis

Bronchoalveolar lavage fluid was harvested by cannulation into the trachea and airways and centrifuged (400 × *g*, 5 min, 4°C). The collected BALF cells were washed and suspended in PBS and then used by fluorescence-activated cell sorting (FACS). For cytological analysis, cells from the BALF were collected on glass slides by centrifugation (400 × *g*, 4 min), fixed, stained with Diff-Quick^®^ Stain (Baxter Healthcare Corp., Miami, FL, USA), and the number of neutrophils was counted.

#### Digestion of pulmonary tissue and cell preparations

Lung tissues were minced and incubated in PBS containing collagenase IV (1 mg/mL) and dispase (2 mg/mL) at 37°C on a shaker. After incubation, the dissolved lung tissues were filtered through a cell strainer and centrifuged (2,000 × *g*, 20 min). The total cell numbers were measured with a hemocytometer.

#### Flow-cytometric analysis

Cells from the BALF and lung tissue were incubated with monoclonal antibodies against CD3, CD4, CD8, CD69, CD62L, CD44, CD25, granulocytic marker Gr-1, and CD11b, CD11c, and B220, washed with FACS buffer, fixed with 0.5% paraformaldehyde, washed again with FACS buffer, and analyzed on a FACSCalibur™ with the CellQuest™ software (BD Biosciences, San Diego, CA, USA).

#### Analysis of inflammatory mediators in BALF

The levels of CXCL-1, MIP-2, IL-17a, and TNF-α in BALF were measured using ELISA kits, according to the manufacturer’s instructions (R&D Systems, St. Louis, MO, USA).

#### Quantitative reverse-transcription polymerase chain reaction (qRT-PCR)

Total RNA from the lung and small intestine was extracted using TRIzol^®^ reagent (Thermo Fisher Scientific), and the total RNA (1 μg) was reverse-transcribed into cDNA using a reverse transcription system (AccuPower RT PreMix, Bioneer, Daejeon, Republic of Korea), according to the manufacturer’s instructions. qRT-PCR was carried out using SYBR™ Green PCR Master Mix on the Applied Biosystems 7,500 Real-Time PCR system, follow a manufacturer’s instructions. The primer sequences are presented in Supplementary Table 1. All the samples were normalized for the corresponding expression of GAPDH, and the expression levels of the gene of interest in treated groups relative to its expression level of the PM_CTL group.

#### Histopathological analysis of lung tissues

Lung tissues were fixed with 10% neutral-buffered formalin, embedded in paraffin, and sliced into 5 μm sections. The tissue sections were stained with hematoxylin–eosin (H&E), Masson’s trichrome (M-T), and periodic acid-Schiff (PAS). Tissue damage were quantified in a blinded manner using a 5-point (0−4) grading system, as previously described (Ozaki et al., 1996).

### Statistical analysis

The data are presented as means ± the standard error of the mean (SEM) and were analyzed by one-way analysis of variance followed by Dunnett’s multiple comparison test using Prism 8.0 software (GraphPad Inc., San Diego, CA, USA). Differences were considered statistically significant at *p* < 0.05.

## Results

### Whole-genome sequence assembly and annotation of *L. paracasei* ATG-E1

**FIGURE 1:**
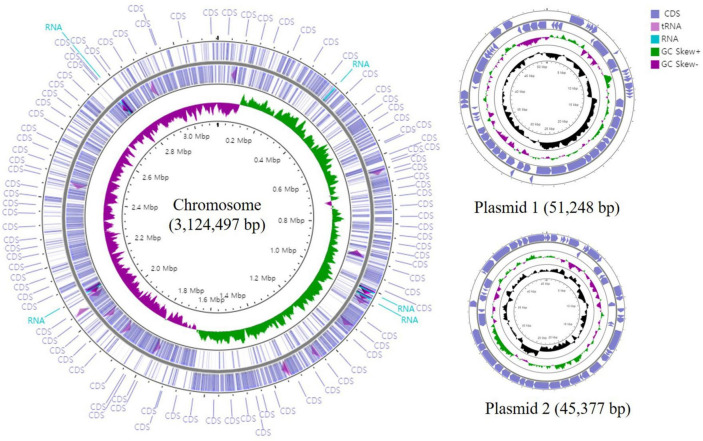
Circular genome map of *L. paracasei* ATG-E1.

The genome of *L. paracasei* ATG-E1 was sequenced using the MinION platform with 320× coverage. The raw sequencing data of ATG-E1 was assembled, results shows a circular chromosome with two plasmids (a total genome size of 3,221,112 bp, Figure 1). The genome was annotated using NCBI PGAAP and uploaded in GenBank under accession number GCA_026013725.1. A total of 3,159 open reading frames (ORFs) were predicted from the genome of strain ATG-E1; 3,081 were protein-coding genes, and 78 were RNA genes (Table 1). Based on ANI analysis, ATG-E1 exhibited 98.37% sequence identity with the type strain *Lactobacillus paracasei* ATCC 25302.

**TABLE 1:** General features of *L. paracasei* ATG-E1.

Attribute	Value of strain ATG-E1
Total genome size (bp)	32,21,122
Chromosome size	31,24,497
Plasmid number	2
DNA G+C (%)	46
Total genes	3,159
Protein coding genes	2,910
rRNA genes	5,5,5 (5S, 16S, 23S)
tRNA genes	60
ncRNA genes	3
Pseudo genes	171
GenBank accession	GCA_02613725.1

### Characterization of *L. paracasei* ATG-E1

The fermentation patterns of carbohydrate sources by *L. paracasei* ATG-E1 are presented in Supplementary Table 2. Based on the pattern identification through the APIWEB database of bioMérieux, the identities (%) of *L. paracasei* ATG-E1 were 99.5% in the *L. paracasei* control group. The gastrointestinal tract is considered to be the main location that affects the viability of probiotics. The survival rate of *L. paracasei* ATG-E1 was 128.13% after 1 h incubation in the environment with a pH = 3.0. As for the simulated 0.3% oxgall solution, the survival rate of *L. paracasei* ATG-E1 was 51.48% (Supplementary Table 3). When *L. paracasei* ATG-E1 was cultured on MRS agar plates supplemented with TDCA, no visible halo surrounding colonies or white precipitates with colonies were observed, indicating that *L. paracasei* ATG-E1 did not have BSH activity (Supplementary Figure 1).

### Safety of *L. paracasei* ATG-E1 confirmed through *in vitro* and whole genome sequence analysis

As shown in Table 2, *L. paracasei* ATG-E1 was sensitive to ampicillin, gentamicin, vancomycin, kanamycin, erythromycin, streptomycin, tetracycline, clindamycin, and chloramphenicol compared to the MIC suggested by the EFSA and Panel on Additives and Products or Substances used in Animal Feed [FEEDAP] (2012). When *L. paracasei* ATG-E1 was grown on sheep blood agar, there was no colorless zone around the colonies, which is considered to indicate γ-hemolytic activity (Table 2), *L. paracasei* ATG-E1 did not produce any biogenic amines, cadaverine, histamine, putrescine, or tyramine from lysine, histidine, ornithine, or tyrosine (Table 2 and Supplementary Figure 2). In further safety tests, the *L. paracasei* ATG-E1 strain was predicted to be a non-human pathogen with PathogenFinder1.1 and did not have antibiotic resistance genes with ResFinder 4.1 (Supplementary Datasheet 1, 2).

**TABLE 2:** *In vitro* safety test of *L. paracasei* ATG-E1.

		MIC (μg/ml)									Biogenic amines			
	Hemolysis	AMP	VAN	GEN	KAN	STR	ERY	TET	CLI	CHL	Tyramine	Histamine	Putrescine	Cadaverine
*Lactobacillus paracasei* ATG-E1	γ	2	N.R	24	48	16	0.125	0.75	0.19	4	−	−	−	−
EFSA and Panel on Additives and Products or Substances used in Animal Feed [FEEDAP], 2012		4	N.R	32	64	64	1	1	4	4				

AMP, ampicillin; VAN, vancomycin; GEN, gentamicin; KAN, kanamycin; STR, streptomycin; ERY, erythromycin; TET, tetracycline; CLI, clindamycin; CHL, chloramphenicol; N.R, not required.

### Effects of *L. paracasei* ATG-E1 on the number of total cells and neutrophils in BALF and lung tissue

**FIGURE 2:**
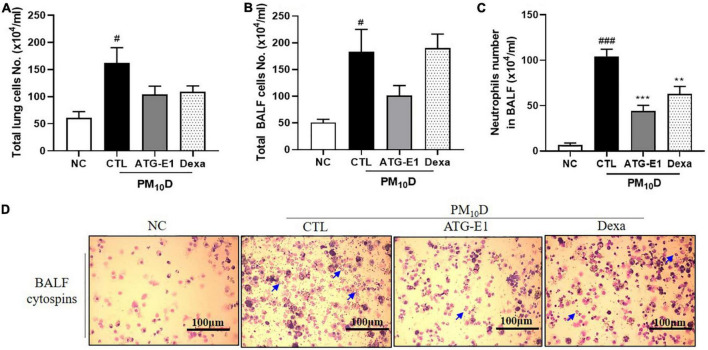
Effects of *L. paracasei* ATG-E1 on cell numbers in a PM_10_D-induced airway inflammation animal. Total number of **(A)** lung cells, **(B)** BALF cells, **(C)** neutrophil number in BALF cytospins, and **(D)** photomicrograph of BALF cytospins. The data are presented as means ± SEM (*n* = 8). Blue arrow represents the neutrophils. ^#^*p* < 0.05 and ^###^*p* < 0.005 vs. NC; **p < 0.01 and ***p < 0.005 vs. CTL. NC: normal mice; CTL: PM_10_D-sensitized control mice; Dexa: 3 mg/kg dexamethasone-treated PM_10_D-sensitized mice; ATG-E1: 4 × 10^9^ CFU/day of *L. paracasei* ATG-E1-treated PM_10_D-sensitized mice.

The total number of cells in the BALF and lungs was higher in the CTL group than in the NC group, but it did not differ in the lungs and BALF after *L. paracasei* ATG-E1 and dexamethasone treatment (Figures 2A, B). The number of neutrophils in BALF in the CTL group was increased than that in the NC group, but the *L. paracasei* ATG-E1- and dexamethasone-treated groups had significantly lower neutrophil infiltration than the CTL group (Figures 2C, D).

### Effects of *L. paracasei* ATG-E1 on lung tissue damage

**FIGURE 3:**
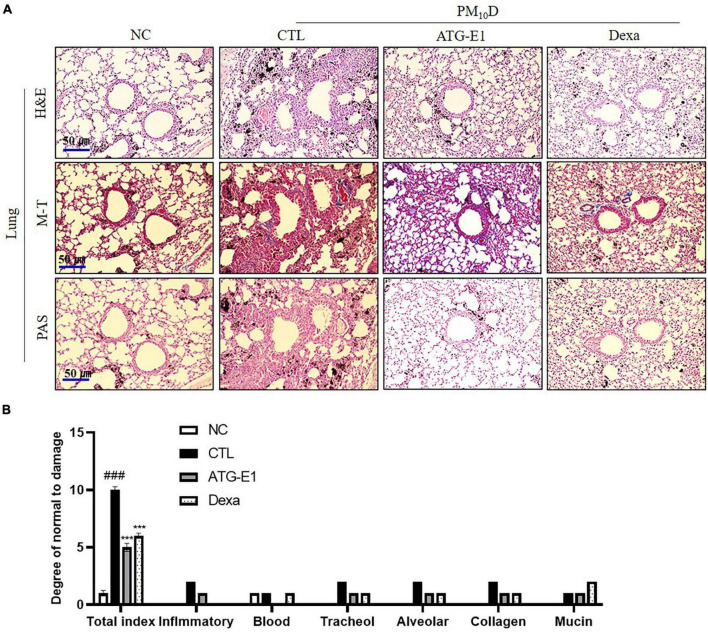
Effects of *L. paracasei* ATG-E1 on histological analysis in a PM_10_D-induced airway inflammation animal. **(A)** H&E, MT, and PAS staining of lung tissues and **(B)** quantitative analyses of the degree of lung tissue damage. The data are presented as means ± SEM (*n* = 8). ^###^*p* < 0.001 vs. NC; ***p < 0.005 vs. CTL. NC: normal mice; CTL: PM_10_D-sensitized control mice; Dexa: 3 mg/kg dexamethasone-treated PM_10_D-sensitized mice; ATG-E1: 4 × 10^9^ CFU/day of *L. paracasei* ATG-E1-treated PM_10_D-sensitized mice.

As shown in Figure 3A, various immune cells, including neutrophils and macrophages, infiltrated into the airway, and destruction of alveolar cells, collagen deposition, and increase of goblet cells were observed in the CTL group compared with the NC group. *L. paracasei* ATG-E1 and dexamethasone treatment recovered these histological scores compared with the CTL group (Figure 3B).

### Effects of *L. paracasei* ATG-E1 on the number of immune cells in BALF and lung tissues

The absolute numbers of lymphocytes, neutrophils, neutrophils to eosinophils ratio, CD4^+^, CD8^+^, CD4^+^CD69^+^, CD62L^–^CD44^+high^, and Gr-1^+^CD11b^+^ cells were increased in the BALF of the CTL group compared with the NC group (Table 3 and Supplementary Figures 3-5). *L. paracasei* ATG-E1 treatment decreased the absolute number of neutrophils and CD4^+^, CD4^+^CD69^+^, and CD62L^–^CD44^+high^ in BALF compared with the CTL group, but there was no change in the absolute number of lymphocytes, neutrophils to eosinophils ratio, CD8^+^, and Gr-1^+^CD11b^+^ cells. The absolute numbers of eosinophils did not differ among treated groups. Dexamethasone treatment did not affect the number of immune cells. In the lungs, the absolute numbers of neutrophils, eosinophils, CD4^+^, CD8^+^, CD4^+^CD69^+^, CD62L^–^CD44^+high^, and Gr-1^+^CD11b^+^ cells were higher in the CTL group than in the NC group, whereas the absolute numbers of these cells, including neutrophils, CD4^+^, CD4^+^CD69^+^, CD62L^–^CD44^+high^, CD21/35^+^B220^+^, and Gr-1^+^CD11b^+^ were decreased by *L. paracasei* ATG-E1 and dexamethasone treatment (Table 3). These findings indicate that *L. paracasei* ATG-E1 effectively inhibited the hyperactivation of the immune system caused by PM_10_D.

**TABLE 3:** The effects of *L. paracasei* ATG-E1 on airway immune cell number in PM_10_D-induced airway inflammation mouse.

Cell phenotype		PM_10_D-induced airway inflammation model (Absolute no.)			
		**NC**	**CTL**	**ATG-E1**	**Dexa_3 mg/kg**
Lymphocytes (× 10^7^ cells)	BALF	0.08 @ 0.02	0.82 @ 0.05^###^	0.87 @ 0.25	0.96 @ 0.53
Neutrophils (× 10^7^ cells)		3.59 @ 0.73	15.09 @ 3.49^##^	7.58 @ 1.40*	15.01 @ 2.14
Eosinophils (× 10^7^ cells)		1.18 @ 0.14	1.82 @ 0.49	1.25 @ 1.15	2.29 @ 0.28
Neutrophils: eosinophils ratio		3.22 @ 0.85	8.52 @ 0.57^#^	5.97 @ 0.61	6.82 @ 1.50
CD4^+^ (× 10^7^ cells)		0.07 @ 0.03	5.26 @ 1.14^###^	2.18 @ 0.48**	3.43 @ 0.58
CD8^+^ (× 10^7^ cells)		0.14 @ 0.08	3.72 @ 0.52^###^	2.54 @ 0.52	2.80 @ 0.71
CD4^+^CD69^+^(× 10^7^ cells)		0.08 @ 0.04	1.73 @ 0.52^##^	0.61 @ 0.10*	1.40 @ 0.19
CD62L^–^CD44^+high^ (× 10^7^ cells)		0.99 @ 0.21	6.40 @ 1.52^##^	2.54 @ 0.41*	5.95 @ 0.85
Gr-1^+^CD11b^+^ (× 10^7^ cells)		0.34 @ 0.06	1.62 @ 0.42^##^	0.79 @ 0.14	1.38 @ 0.12
Lymphocytes (× 10^7^ cells)	Lung	2.60 @ 0.64	3.17 @ 0.51	4.39 @ 0.83	9.34 @ 6.56
Neutrophils (× 10^7^ cells)		3.03 @ 0.48	12.04 @ 2.26^##^	5.06 @ 0.67*	3.41 @ 0.83**
Eosinophils (× 10^7^ cells)		1.56 @ 0.20	6.87 @ 0.97^###^	4.28 @ 1.02	3.41 @ 1.06
CD4^+^ (× 10^7^ cells)		1.97 @ 0.35	6.13 @ 1.08^##^	3.39 @ 0.62*	3.32 @ 0.42*
CD8^+^ (× 10^7^ cells)		1.11 @ 0.23	3.46 @ 0.78^##^	1.84 @ 0.28	1.92 @ 0.18
CD4^+^CD69^+^(× 10^7^ cells)		0.20 @ 0.08	3.01 @ 1.08^#^	0.46 @ 0.11*	0.29 @ 0.07*
CD62L^–^CD44^+high^ (× 10^7^ cells)		0.35 @ 0.05	2.53 @ 0.50^##^s	0.87 @ 0.06*	1.27 @ 0.15
CD21/35^+^B220^+^(× 10^7^ cells)		0.27 @ 0.09	3.40 @ 0.72^###^	1.81 @ 0.35*	1.35 @ 0.32**
Gr-1^+^CD11b^+^ (× 10^7^ cells)		0.17 @ 0.05	1.07 @ 0.09^###^	0.38 @ 0.06***	0.60 @ 0.08***

The data are presented as means ± SEM (n = 8). ^#^p < 0.05, ^##^p < 0.01 and ^###^p < 0.005 vs. NC; *p < 0.05, **p < 0.01 and ***p < 0.005 vs. CTL. NC: BALB/c normal mice; CTL: PM_10_D-sensitized control mice; Dexa: 3 mg/kg dexamethasone-treated PM_10_D-sensitized mice; ATG-E1: 4 × 10^9^ CFU/day of L. paracasei ATG-E1-treated PM_10_D-sensitized mice.

### Effects of *L. paracasei* ATG-E1 on inflammatory mediators in BALF and lung

**FIGURE 4:**
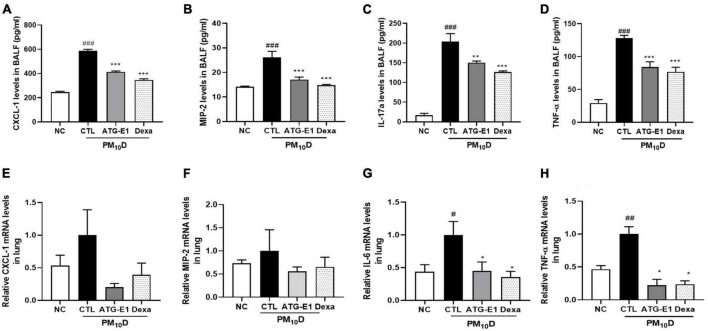
Effects of *L. paracasei* ATG-E1 on proinflammatory mediators in BALF and lung of a PM_10_D-induced airway inflammation animal. **(A)** CXCL-1, **(B)** MIP-2, **(C)** IL-17a, and **(D)** TNF-α levels in the BALF. **(E)** CXCL-1, **(F)** MIP-2, **(G)** IL-6, and **(H)** TNF-α gene expression levels in the lung. The data are presented as means ± SEM (*n* = 8). ^#^*p* < 0.05, ^##^*p* < 0.01, and ^###^*p* < 0.005 vs. NC; *p < 0.05, **p < 0.01 and ***p < 0.005 vs. CTL. NC: normal mice; CTL: PM_10_D-sensitized control mice; Dexa: 3 mg/kg dexamethasone-treated PM_10_D-sensitized mice; ATG-E1: 4 × 10^9^ CFU/day of *L. paracasei* ATG-E1-treated PM_10_D-sensitized mice.

CXCL-1, MIP-2, IL-17a, and TNF-α levels in BALF of the CTL group were increased than those of the NC group, but *L. paracasei* ATG-E1 and dexamethasone treatment significantly suppressed the levels of these cytokines and chemokines compared with the CTL group (Figures 4A–D). In the lungs, CXCL-1 and MIP-2 mRNA expression levels showed a reduction tendency, although there were no significant differences (Figures 4E, F). IL-6 and TNF-α mRNA expression levels were higher in the CTL group than in the NC group, but these gene expression levels were lowered by administration of *L. paracasei* ATG-E1 and dexamethasone (Figures 4G, H). These results demonstrate that *L. paracasei* ATG-E1 may downregulate cytokine expression during PM_10_D-induced airway inflammation.

### Effects of *L. paracasei* ATG-E1 on intestinal immunity

**FIGURE 5:**
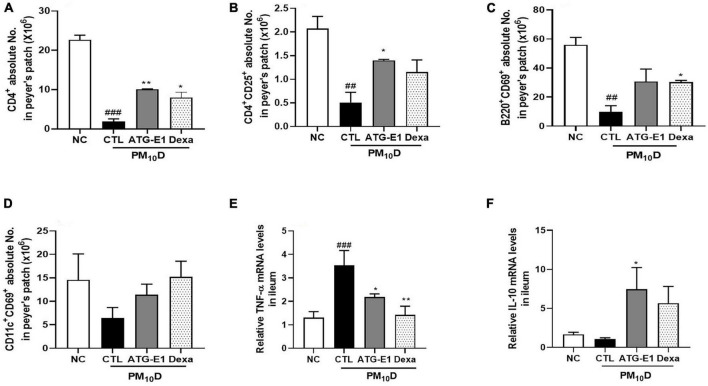
Effects of *L. paracasei* ATG-E1 on immune cell numbers and cytokine expression levels in the peyer’s patch and the ileum of a PM_10_D-induced airway inflammation animal. **(A)** CD4^+^, **(B)** CD4^+^CD69^+^, **(C)** B220^+^CD69^+^, and **(D)** CD11c^+^CD69^+^ absolute cell numbers in peyer’s patch. **(E)** TNF-α, and **(F)** IL-10 mRNA expression levels in the ileum. The data are presented as means ± SEM (*n* = 8). ^##^*p* < 0.01 and ^###^*p* < 0.05 vs. NC; *p < 0.05 and **p < 0.01 vs. CTL. NC: normal mice; CTL: PM_10_D-sensitized control mice; Dexa: 3 mg/kg dexamethasone-treated PM_10_D-sensitized mice; ATG-E1: 4 × 10^9^ CFU/day of *L. paracasei* ATG-E1-treated PM_10_D-sensitized mice.

In peyer’s patch, the absolute numbers of CD4^+^, CD4^+^CD25^+^, and B220^+^CD69^+^ cells were decreased in the CTL group compared with the NC group. However, the absolute number of CD4^+^ and CD4^+^CD25^+^ cells increased, and the absolute number of B220^+^CD69^+^ cells increased in the *L. paracasei* ATG-E1-treated group (Figures 5A–C). In the case of CD11c^+^CD69^+^, the absolute cell number showed an increasing tendency compared with that in the CTL group after administration of ATG-E1 (Figure 5D). Dexamethasone treatment resulted in a significant increase in the number of CD4^+^ and B220^+^CD69^+^ cells. Next, we investigated the mRNA expression of inflammatory cytokines in the small intestine. TNF-α mRNA expression levels were upregulated in the CTL group than in the NC group, while were downregulated in the *L. paracasei* ATG-E1- and dexamethasone-treated groups (Figure 5E). IL-10 mRNA expression levels did not differ among the NC-, CTL-, and dexamethasone-treated groups, but their expression levels were increased by *L. paracasei* ATG-E1 (Figure 5F).

### Effects of *L. paracasei* ATG-E1 on gut barrier function

**FIGURE 6:**
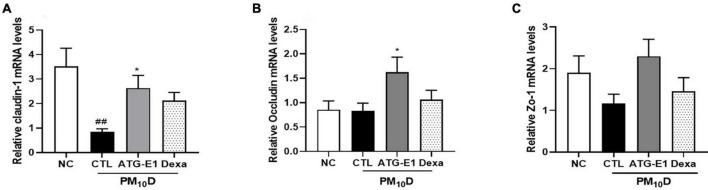
Effects of *L. paracasei* ATG-E1 on the expression of tight junction-related genes in the ileum of a PM_10_D-induced airway inflammation animal. **(A)** Occludin, **(B)** claudin-1, and **(C)** ZO-1 mRNA expression levels in the ileum. The data are presented as means ± SEM (*n* = 8). ^##^*p* < 0.01 vs. NC; *p < 0.05 vs. CTL. NC: normal mice; CTL: PM_10_D-sensitized control mice; Dexa: 3 mg/kg dexamethasone-treated PM_10_D-sensitized mice; ATG-E1: 4 × 10^9^ CFU/day of *L. paracasei* ATG-E1-treated PM_10_D-sensitized mice.

Claudin-1 mRNA expression levels were downregulated in the ileum of the CTL group compared to the NC group, while they were upregulated by *L. paracasei* ATG-E1 compared to the CTL group (Figure 6A). Occludin mRNA expression levels were also increased by *L. paracasei* ATG-E1, although there was no difference between the NC-, CTL-, and dexamethasone-treated groups (Figure 6B). ZO-1 mRNA expression levels in the *L. paracasei* ATG-E1 group showed a tendency to increase compared with the CTL group, but did not significant (Figure 6C).

## Discussion

The application of new probiotic strains as functional health foods and therapies requires careful consideration with regard to safety, although probiotics, especially *Lactobacillus*, are generally considered to be safe. In the present study, we performed genetic and phenotypic analyses of *L. paracasei* ATG-E1 isolated from feces of a newborn baby and *in vitro* safety analysis based on international guidelines for the evaluation of probiotics recommended by the FAO/WHO (2002). *L. paracasei* ATG-E1 did not exhibit antibiotic resistance, toxin production, or hemolytic activity. In addition, there were no antibiotic resistance- or pathogen-related genes in the genome sequence of *L. paracasei* ATG-E1. These results demonstrate that this strain is safe for use in the development of probiotics.

Exposure to PM and DEP can interact with the immune system and enhance inflammatory responses in the airways and lungs. They cause infiltration of neutrophils and macrophages into the airways and lungs, with increased proinflammatory cytokine levels, in addition to inducing an antigen-presenting cell-mediated inflammatory response and imbalance of T-helper 1 cells (Losacco and Perillo, 2018; Tiotiu et al., 2020). Neutrophils are considered a hallmark of pulmonary inflammation caused by PM since they play an important role in the PM-mediated inflammatory cells infiltration to the airway (Becker et al., 2005; Sarir et al., 2008; Møller et al., 2014). CD4^+^CD69^+^ cells are activated by T cells and suppress the production of proinflammatory cytokines (Ishikawa et al., 1998). CD62L^–^CD44^+high^ markers are increased on naive T cells after activation and have been required for activated T cell extravasation into inflammation site. Additionally, PM has been known to increase the number of CD62L^–^CD44^+high^ cells (DeGrendele et al., 1997; Deiuliis et al., 2012). CD21/35^+^B220^+^ are markers of mature B cells and are involved in T cell-dependent responses (Haas et al., 2002). Gr-1^+^CD11b^+^ markers on the surface of granulocytes are involved in inflammation and cytotoxic T-cell inhibition (Lee et al., 2010). Among chemokines and cytokines, CXCL1, and MIP2 recruit neutrophils and lymphocytes and mediate the neutrophilic inflammatory response in lungs, subsequently promoting the expression of other chemokines (Tsujimoto et al., 2002; Sawant et al., 2015). TNF-α is a pleiotropic cytokine associated with inflammation and immunomodulation and involved in the pathogenesis of lung inflammation (Matera et al., 2010). In the present study, we found that *L. paracasei* ATG-E1 reduced the number of neutrophils and various immune cells, including CD4^+^, CD4^+^CD69^+^, and CD62L^–^CD44^+high^ cells, with a tendency of reduced Gr-1^+^CD11b^+^ cells in BALF, but also a decreased number of CD21/35B220^+^ and Gr-1^+^CD11b^+^ immune cells in the lung. In addition, *L. paracasei* ATG-E1 suppressed the expression of various proinflammatory cytokines, such as CXCL-1, MIP2, IL-17A, and TNF-α, in the BALF and lungs. These results indicated that *L. paracasei* ATG-E1 effectively inhibited PM_10_D-induced immune activation, consequently suppressing inflammation.

Particulate matter and DEP can result in histopathological changes accompanied by an increase in inflammatory cell infiltration, oxidative products, various cytokines and chemokines, mucus production, and epithelial thickness (Curtis et al., 1991). We observed that airway inflammation and airway wall thickening in the lung were reduced by *L. paracasei* ATG-E1 treatment. These results indicate that *L. paracasei* ATG-E1 protects the lung against PM_10_D-induced histopathological changes.

Although the lungs are the primary organ exposed to PM and DEP, several studies have reported that exposure to PM is also linked to adverse health effects in the gastrointestinal tract (Kaplan et al., 2010). A part of their exposure can be deposited in the gastrointestinal tract directly or indirectly through swallowing of the PM and DEP deposited in the upper airway (Mutlu et al., 2011). PM and DEP may perturb barrier integrity by affecting the expression of tight junctions and induce inflammation in the gut (Mutlu et al., 2011; Wang et al., 2012). In addition, it has been reported that oral probiotic administration affects immune response in intestine by inducing Treg cells such as CD4^+^CD25^+^Foxp3^+^ cells and immunosuppressive cytokines including IL-10 and transforming growth factor-β (Segawa et al., 2008; Lim et al., 2017; Song et al., 2019), Although the exact mechanisms of immunomodulation exerted by probiotics are not fully understood yet, they also improve gut barrier function. *L. paracasei* ATG-E1 increased the number of CD4^+^CD25^+^ cells and IL-10 expression, while decreasing TNF-α expression in the peyer’s patch and ileum. These results demonstrate that *L. paracasei* ATG-E1 may have beneficial effects on intestinal immunity by inducing a Treg response; however, further studies are needed in this regard. In addition, we observed that *L. paracasei* ATG-E1 increased the expression of tight junction genes claudin-1 and occludin-1 in the ileum, suggesting that *L. paracasei* ATG-E1 may improve barrier integrity impaired by PM_10_D. These results indicate that *L. paracasei* ATG-E1 has beneficial effects on immunity and barrier function in the intestine during PM_10_D-induced airway inflammation.

In conclusion, *L. paracasei* ATG-E1 was safe and effectively ameliorated lung tissue damage and inhibited airway inflammation by regulation of the immune system. It also regulated intestinal immunity, with improved gut barrier function in PM_10_D-induced airway inflammation. Therefore, the findings of the present study suggest that *L. paracasei* ATG-E1 exerts preventive and protective effects against airway inflammation and respiratory diseases.

## Data availability statement

The original contributions presented in this study are included in the article/Supplementary material, further inquiries can be directed to the corresponding author.

## Ethics statement

This animal study was reviewed and approved by the Committee for Animal Welfare at Daejeon University.

## Author contributions

Y-SL designed and performed the experiments, analyzed the data, and wrote and supervised the drafting of the manuscript. S-HKo and G-SP performed the whole-genome analysis, genome assembly, gene prediction, and safety assessment and wrote the manuscript. W-KY, H-JS, S-HKi, and NJ performed the experiments and analyzed the data. JK supervised the drafting of the manuscript. All authors contributed to the manuscript and approved the final manuscript.

## Footnotes

1
https://community.nanoporetech.com/downloads

2
http://www.ezbiocloud.net/
