# Supplementary material for: Lactobacillus paracasei ATG-E1 improves particulate matter 10 plus diesel exhaust particles (PM10D)-induced airway inflammation by regulating immune responses
Source: Front Microbiol. 2023 Apr 27;14:1145546. doi: 10.3389/fmicb.2023.1145546 (PMC10174254; doi:10.3389/fmicb.2023.1145546)
Supplement: Supplementary file 1 [file Data_Sheet_1.pdf]

Center for Genomic Epidemiology

Home Services Instructions Output

The input organism was predicted as non human pathogen

**Probability of being a human pathogen**	0.188
**Input proteome coverage (%)**	15.6
**Matched Pathogenic Families**	0
**Matched Not Pathogenic Families**	480

**Sequences**	3076
**Total bpp**	886996
**Longest seq**	2240
**Shortest seq**	30
**Avg seq lenght**	288.0

Input Sequence: ATG-E1_Chromosome_917 # 928824 # 929573 # -1 # ID=1_917;partial=00;start_type=ATG;rbs_motif=None;rbs_spacer=None;gc_cont=0.464

	PROJECT ID	ACCESSION ID	ORGANISMS	CLASS	PROTEIN FUNCTION	PROTEIN ID	%IDENTITY
**Matched Family**	28537	CP001084	Lactobacillus casei str. Zhang, complete genome.	Lactobacillales	Cation transport ATPase	ADK17977	100.0

Input Sequence: ATG-E1_Chromosome_2169 # 2179347 # 2181638 # -1 # ID=1_2169;partial=00;start_type=GTG;rbs_motif=GGA/GAG/AGG;rbs_spacer=5-10bp;gc_cont=0.471

	PROJECT ID	ACCESSION ID	ORGANISMS	CLASS	PROTEIN FUNCTION	PROTEIN ID	%IDENTITY
**Matched Family**	28537	CP001084	Lactobacillus casei str. Zhang, complete genome.	Lactobacillales	Superfamily I DNA and RNA helicase	ADK19132	100.0

Input Sequence: ATG-E1_Chromosome_2843 # 2885127 # 2887415 # 1 # ID=1_2843;partial=00;start_type=ATG;rbs_motif=AGGAG;rbs_spacer=5-10bp;gc_cont=0.478

	PROJECT ID	ACCESSION ID	ORGANISMS	CLASS	PROTEIN FUNCTION	PROTEIN ID	%IDENTITY
**Matched Family**	402	CP000423	Lactobacillus casei ATCC 334, complete genome.	Lactobacillales	Superfamily I DNA and RNA helicase	ABJ71350	100.0

Input Sequence: ATG-E1_Chromosome_1900 # 1913032 # 1915158 # -1 # ID=1_1900;partial=00;start_type=GTG;rbs_motif=AGGAG;rbs_spacer=5-10bp;gc_cont=0.465

	PROJECT ID	ACCESSION ID	ORGANISMS	CLASS	PROTEIN FUNCTION	PROTEIN ID	%IDENTITY
**Matched Family**	28537	CP001084	Lactobacillus casei str. Zhang, complete genome.	Lactobacillales	Cell division protein FtsI/penicillin-binding protein 2	ADK18880	100.0

Input Sequence: ATG-E1_Chromosome_2536 # 2576948 # 2579002 # 1 # ID=1_2536;partial=00;start_type=ATG;rbs_motif=AGGAGG;rbs_spacer=5-10bp;gc_cont=0.475

	PROJECT ID	ACCESSION ID	ORGANISMS	CLASS	PROTEIN FUNCTION	PROTEIN ID	%IDENTITY
**Matched Family**	30359	FM177140	Lactobacillus casei BL23 complete genome, strain BL23.	Lactobacillales	Probable potassium transport system protein kup	CAQ67584	100.0

Input Sequence: ATG-E1_Chromosome_1571 # 1560289 # 1562268 # 1 # ID=1_1571;partial=00;start_type=TTG;rbs_motif=GGxGG;rbs_spacer=5-10bp;gc_cont=0.448

	PROJECT ID	ACCESSION ID	ORGANISMS	CLASS	PROTEIN FUNCTION	PROTEIN ID	%IDENTITY
**Matched Family**	30359	FM177140	Lactobacillus casei BL23 complete genome, strain BL23.	Lactobacillales	Acyltransferase 3	CAQ66654	100.0

Input Sequence: ATG-E1_Chromosome_661 # 680622 # 681368 # 1 # ID=1_661;partial=00;start_type=GTG;rbs_motif=GGAGG;rbs_spacer=5-10bp;gc_cont=0.438

	PROJECT ID	ACCESSION ID	ORGANISMS	CLASS	PROTEIN FUNCTION	PROTEIN ID	%IDENTITY
**Matched Family**	30359	FM177140	Lactobacillus casei BL23 complete genome, strain BL23.	Lactobacillales	Putative membrane-bound ATPase	CAQ65720	100.0

Input Sequence: ATG-E1_Chromosome_2947 # 2995810 # 2997678 # -1 # ID=1_2947;partial=00;start_type=TTG;rbs_motif=GGAGG;rbs_spacer=5-10bp;gc_cont=0.414

PROJECT	ACCESSION ID	ORGANISMS	CLASS	PROTEIN FUNCTION	PROTEIN ID	%IDENTITY

	ID						
**Matched Family**	28537	CP001084	Lactobacillus casei str. Zhang, complete genome.	Lactobacillales	sorbitol operon transcription regulator	ADK19916	100.0

Input Sequence ATG-E1_Chromosome_2177 # 2189166 # 2190974 # -1 # ID=1_2177;partial=00;start_type=ATG;rbs_motif=GGA/GAG/AGG;rbs_spacer=5-10bp;gc_cont=0.464

	PROJECT ID	ACCESSION ID	ORGANISMS	CLASS	PROTEIN FUNCTION	PROTEIN ID	%IDENTITY
**Matched Family**	28537	CP001084	Lactobacillus casei str. Zhang, complete genome.	Lactobacillales	ABC-type antimicrobial peptide transport system, permease component	ADK19140	100.0

Input Sequence ATG-E1_Chromosome_1076 # 1091345 # 1093012 # 1 # ID=1_1076;partial=00;start_type=ATG;rbs_motif=GGAGG;rbs_spacer=5-10bp;gc_cont=0.486

	PROJECT ID	ACCESSION ID	ORGANISMS	CLASS	PROTEIN FUNCTION	PROTEIN ID	%IDENTITY
**Matched Family**	28537	CP001084	Lactobacillus casei str. Zhang, complete genome.	Lactobacillales	Signal transduction histidine kinase	ADK18125	100.0

Input Sequence ATG-E1_Chromosome_268 # 282263 # 283888 # 1 # ID=1_268;partial=00;start_type=ATG;rbs_motif=GGAG/GAGG;rbs_spacer=5-10bp;gc_cont=0.451

	PROJECT ID	ACCESSION ID	ORGANISMS	CLASS	PROTEIN FUNCTION	PROTEIN ID	%IDENTITY
**Matched Family**	28537	CP001084	Lactobacillus casei str. Zhang, complete genome.	Lactobacillales	ABC-type oligopeptide transport system, periplasmic component	ADK17497	100.0

Input Sequence ATG-E1_Chromosome_2012 # 2023677 # 2025212 # 1 # ID=1_2012;partial=00;start_type=ATG;rbs_motif=GGAGG;rbs_spacer=5-10bp;gc_cont=0.420

	PROJECT ID	ACCESSION ID	ORGANISMS	CLASS	PROTEIN FUNCTION	PROTEIN ID	%IDENTITY
**Matched Family**	28537	CP001084	Lactobacillus casei str. Zhang, complete genome.	Lactobacillales	conserved hypothetical protein	ADK18989	100.0

Input Sequence ATG-E1_Chromosome_2602 # 2642280 # 2643809 # -1 # ID=1_2602;partial=00;start_type=ATG;rbs_motif=AGxAGG/AGGxGG;rbs_spacer=5-10bp;gc_cont=0.495

	PROJECT ID	ACCESSION ID	ORGANISMS	CLASS	PROTEIN FUNCTION	PROTEIN ID	%IDENTITY
**Matched Family**	28537	CP001084	Lactobacillus casei str. Zhang, complete genome.	Lactobacillales	Putative multicopper oxidase	ADK19590	100.0

Input Sequence ATG-E1_Chromosome_2070 # 2090326 # 2091855 # 1 # ID=1_2070;partial=00;start_type=ATG;rbs_motif=AGxAGG/AGGxGG;rbs_spacer=5-10bp;gc_cont=0.439

	PROJECT ID	ACCESSION ID	ORGANISMS	CLASS	PROTEIN FUNCTION	PROTEIN ID	%IDENTITY
**Matched Family**	402	CP000423	Lactobacillus casei ATCC 334, complete genome.	Lactobacillales	hypothetical protein	ABJ70597	100.0

Input Sequence ATG-E1_Chromosome_2830 # 2868981 # 2870510 # -1 # ID=1_2830;partial=00;start_type=ATG;rbs_motif=GGxGG;rbs_spacer=5-10bp;gc_cont=0.434

	PROJECT ID	ACCESSION ID	ORGANISMS	CLASS	PROTEIN FUNCTION	PROTEIN ID	%IDENTITY
**Matched Family**	28537	CP001084	Lactobacillus casei str. Zhang, complete genome.	Lactobacillales	Exopolyphosphatase	ADK19773	100.0

Input Sequence ATG-E1_Chromosome_1083 # 1098698 # 1100179 # 1 # ID=1_1083;partial=00;start_type=ATG;rbs_motif=GGAGG;rbs_spacer=5-10bp;gc_cont=0.471

	PROJECT ID	ACCESSION ID	ORGANISMS	CLASS	PROTEIN FUNCTION	PROTEIN ID	%IDENTITY
**Matched Family**	28537	CP001084	Lactobacillus casei str. Zhang, complete genome.	Lactobacillales	conserved hypothetical protein	ADK18132	100.0

Input Sequence ATG-E1_Chromosome_98 # 109499 # 110977 # 1 # ID=1_98;partial=00;start_type=ATG;rbs_motif=GGAG/GAGG;rbs_spacer=5-10bp;gc_cont=0.499

	PROJECT ID	ACCESSION ID	ORGANISMS	CLASS	PROTEIN FUNCTION	PROTEIN ID	%IDENTITY
**Matched Family**	28537	CP001084	Lactobacillus casei str. Zhang, complete genome.	Lactobacillales	Cytochrome bd-type quinol oxidase, subunit 1	ADK17313	100.0

Input Sequence ATG-E1_Chromosome_1691 # 1689722 # 1691173 # -1 # ID=1_1691;partial=00;start_type=ATG;rbs_motif=AGGAGG;rbs_spacer=5-10bp;gc_cont=0.492

	PROJECT ID	ACCESSION ID	ORGANISMS	CLASS	PROTEIN FUNCTION	PROTEIN ID	%IDENTITY
**Matched**	402	CP000423	Lactobacillus casei ATCC 334,	Lactobacillales	hypothetical protein	ABJ70244	100.0

Family			complete genome.				

**Input Sequence**	ATG-E1_Chromosome_2263 # 2286904 # 2288334 # -1 # ID=1_2263;partial=00;start_type=ATG;rbs_motif=GGA/GAG/AGG;rbs_spacer=5-10bp;gc_cont=0.492						
	PROJECT ID	ACCESSION ID	ORGANISMS	CLASS	PROTEIN FUNCTION	PROTEIN ID	%IDENTITY
**Matched Family**	28537	CP001084	Lactobacillus casei str. Zhang, complete genome.	Lactobacillales	efflux pump antibiotic resistance protein	ADK19237	100.0

**Input Sequence**	ATG-E1_Chromosome_2054 # 2069742 # 2071112 # 1 # ID=1_2054;partial=00;start_type=ATG;rbs_motif=GGA/GAG/AGG;rbs_spacer=5-10bp;gc_cont=0.454						
	PROJECT ID	ACCESSION ID	ORGANISMS	CLASS	PROTEIN FUNCTION	PROTEIN ID	%IDENTITY
**Matched Family**	30359	FM177140	Lactobacillus casei BL23 complete genome, strain BL23.	Lactobacillales	Putative melibiose permease II	CAQ67114	100.0

**Input Sequence**	ATG-E1_Chromosome_2417 # 2454383 # 2455774 # 1 # ID=1_2417;partial=00;start_type=ATG;rbs_motif=GGAG/GAGG;rbs_spacer=5-10bp;gc_cont=0.431						
	PROJECT ID	ACCESSION ID	ORGANISMS	CLASS	PROTEIN FUNCTION	PROTEIN ID	%IDENTITY
**Matched Family**	28537	CP001084	Lactobacillus casei str. Zhang, complete genome.	Lactobacillales	Hemolysin-related protein with CBS domains	ADK19404	100.0

**Input Sequence**	ATG-E1_Chromosome_3014 # 3060022 # 3061398 # -1 # ID=1_3014;partial=00;start_type=GTG;rbs_motif=AGxAGG/AGGxGG;rbs_spacer=5-10bp;gc_cont=0.450						
	PROJECT ID	ACCESSION ID	ORGANISMS	CLASS	PROTEIN FUNCTION	PROTEIN ID	%IDENTITY
**Matched Family**	28537	CP001084	Lactobacillus casei str. Zhang, complete genome.	Lactobacillales	ABC-type sugar transport system, periplasmic component	ADK19960	100.0

**Input Sequence**	ATG-E1_Chromosome_2601 # 2640867 # 2642240 # -1 # ID=1_2601;partial=00;start_type=ATG;rbs_motif=None;rbs_spacer=None;gc_cont=0.501						
	PROJECT ID	ACCESSION ID	ORGANISMS	CLASS	PROTEIN FUNCTION	PROTEIN ID	%IDENTITY
**Matched Family**	30359	FM177140	Lactobacillus casei BL23 complete genome, strain BL23.	Lactobacillales	Permease of the major facilitator superfamily	CAQ67652	100.0

**Input Sequence**	ATG-E1_Chromosome_216 # 231545 # 232072 # -1 # ID=1_216;partial=00;start_type=ATG;rbs_motif=AGxAG;rbs_spacer=5-10bp;gc_cont=0.394						
	PROJECT ID	ACCESSION ID	ORGANISMS	CLASS	PROTEIN FUNCTION	PROTEIN ID	%IDENTITY
**Matched Family**	30359	FM177140	Lactobacillus casei BL23 complete genome, strain BL23.	Lactobacillales	FAD/FMN-containing dehydrogenase	CAQ65259	100.0

**Input Sequence**	ATG-E1_Chromosome_2834 # 2873651 # 2875006 # -1 # ID=1_2834;partial=00;start_type=ATG;rbs_motif=GGAGG;rbs_spacer=5-10bp;gc_cont=0.493						
	PROJECT ID	ACCESSION ID	ORGANISMS	CLASS	PROTEIN FUNCTION	PROTEIN ID	%IDENTITY
**Matched Family**	28537	CP001084	Lactobacillus casei str. Zhang, complete genome.	Lactobacillales	Na+-driven multidrug efflux pump	ADK19777	100.0

**Input Sequence**	ATG-E1_Chromosome_2567 # 2609980 # 2611335 # -1 # ID=1_2567;partial=00;start_type=ATG;rbs_motif=AGGA/GGAG/GAGG;rbs_spacer=11-12bp;gc_cont=0.500						
	PROJECT ID	ACCESSION ID	ORGANISMS	CLASS	PROTEIN FUNCTION	PROTEIN ID	%IDENTITY
**Matched Family**	30359	FM177140	Lactobacillus casei BL23 complete genome, strain BL23.	Lactobacillales	Putative uncharacterized protein	CAQ67619	100.0

**Input Sequence**	ATG-E1_Chromosome_1494 # 1479903 # 1481249 # 1 # ID=1_1494;partial=00;start_type=ATG;rbs_motif=AGGAGG;rbs_spacer=5-10bp;gc_cont=0.489						
	PROJECT ID	ACCESSION ID	ORGANISMS	CLASS	PROTEIN FUNCTION	PROTEIN ID	%IDENTITY
**Matched Family**	28537	CP001084	Lactobacillus casei str. Zhang, complete genome.	Lactobacillales	ATPase for cell division	ADK18499	100.0

**Input Sequence**	ATG-E1_Chromosome_241 # 252309 # 253529 # -1 # ID=1_241;partial=00;start_type=ATG;rbs_motif=GGA/GAG/AGG;rbs_spacer=5-10bp;gc_cont=0.423						
	PROJECT ID	ACCESSION ID	ORGANISMS	CLASS	PROTEIN FUNCTION	PROTEIN ID	%IDENTITY
**Matched Family**	28537	CP001084	Lactobacillus casei str. Zhang, complete genome.	Lactobacillales	hypothetical protein	ADK17467	100.0

Input Sequence	ATG-E1_Chromosome_3049 # 3091557 # 3092900 # -1 # ID=1_3049;partial=00;start_type=ATG;rbs_motif=AGxAGG/AGGxGG;rbs_spacer=5-10bp;gc_cont=0.501					
	PROJECT ID	ACCESSION ID	ORGANISMS	CLASS	PROTEIN FUNCTION	PROTEIN ID	%IDENTITY
Matched Family	402	CP000423	Lactobacillus casei ATCC 334, complete genome.	Lactobacillales	Acetylornithine deacetylase/Succinyl-diaminopimelate desuccinylase related deacylase	ABJ71510	100.0

Input Sequence	ATG-E1_Chromosome_2879 # 2925785 # 2926222 # -1 # ID=1_2879;partial=00;start_type=ATG;rbs_motif=None;rbs_spacer=None;gc_cont=0.466						
	PROJECT ID	ACCESSION ID	ORGANISMS	CLASS	PROTEIN FUNCTION	PROTEIN ID	%IDENTITY
Matched Family	28537	CP001084	Lactobacillus casei str. Zhang, complete genome.	Lactobacillales	PTS system, cellobiose-specific enzyme II, C component	ADK19831	100.0

Input Sequence	ATG-E1_Chromosome_281 # 296916 # 298229 # 1 # ID=1_281;partial=00;start_type=ATG;rbs_motif=AGGAGG;rbs_spacer=5-10bp;gc_cont=0.501						
	PROJECT ID	ACCESSION ID	ORGANISMS	CLASS	PROTEIN FUNCTION	PROTEIN ID	%IDENTITY
Matched Family	28537	CP001084	Lactobacillus casei str. Zhang, complete genome.	Lactobacillales	Beta-propeller domains of methanol dehydrogenase type	ADK17508	100.0

Input Sequence	ATG-E1_Chromosome_1129 # 1150507 # 1151820 # -1 # ID=1_1129;partial=00;start_type=ATG;rbs_motif=AGGA/GGAG/GAGG;rbs_spacer=11-12bp;gc_cont=0.449						
	PROJECT ID	ACCESSION ID	ORGANISMS	CLASS	PROTEIN FUNCTION	PROTEIN ID	%IDENTITY
Matched Family	28537	CP001084	Lactobacillus casei str. Zhang, complete genome.	Lactobacillales	ABC-type sugar transport system, periplasmic component	ADK18180	100.0

Input Sequence	ATG-E1_Chromosome_1601 # 1591919 # 1593187 # 1 # ID=1_1601;partial=00;start_type=ATG;rbs_motif=GGAGG;rbs_spacer=5-10bp;gc_cont=0.484						
	PROJECT ID	ACCESSION ID	ORGANISMS	CLASS	PROTEIN FUNCTION	PROTEIN ID	%IDENTITY
Matched Family	28537	CP001084	Lactobacillus casei str. Zhang, complete genome.	Lactobacillales	TPR repeats containing protein	ADK18602	100.0

Input Sequence	ATG-E1_Chromosome_2928 # 2978495 # 2979754 # 1 # ID=1_2928;partial=00;start_type=ATG;rbs_motif=GGxGG;rbs_spacer=5-10bp;gc_cont=0.452						
	PROJECT ID	ACCESSION ID	ORGANISMS	CLASS	PROTEIN FUNCTION	PROTEIN ID	%IDENTITY
Matched Family	28537	CP001084	Lactobacillus casei str. Zhang, complete genome.	Lactobacillales	putative PTS system, IIc component	ADK19897	100.0

Input Sequence	ATG-E1_Chromosome_109 # 116308 # 117543 # -1 # ID=1_109;partial=00;start_type=ATG;rbs_motif=GGAG/GAGG;rbs_spacer=5-10bp;gc_cont=0.478						
	PROJECT ID	ACCESSION ID	ORGANISMS	CLASS	PROTEIN FUNCTION	PROTEIN ID	%IDENTITY
Matched Family	30359	FM177140	Lactobacillus casei BL23 complete genome, strain BL23.	Lactobacillales	Surface antigen	CAQ65155	100.0

Input Sequence	ATG-E1_Chromosome_1001 # 1015266 # 1016474 # 1 # ID=1_1001;partial=00;start_type=GTG;rbs_motif=GGAGG;rbs_spacer=5-10bp;gc_cont=0.469						
	PROJECT ID	ACCESSION ID	ORGANISMS	CLASS	PROTEIN FUNCTION	PROTEIN ID	%IDENTITY
Matched Family	30359	FM177140	Lactobacillus casei BL23 complete genome, strain BL23.	Lactobacillales	Glycosyltransferase	CAQ66021	100.0

Input Sequence	ATG-E1_Chromosome_2859 # 2902352 # 2903554 # 1 # ID=1_2859;partial=00;start_type=ATG;rbs_motif=AGxAGG/AGGxGG;rbs_spacer=5-10bp;gc_cont=0.470						
	PROJECT ID	ACCESSION ID	ORGANISMS	CLASS	PROTEIN FUNCTION	PROTEIN ID	%IDENTITY
Matched Family	28537	CP001084	Lactobacillus casei str. Zhang, complete genome.	Lactobacillales	HTH containing DNA-binding domain and MocR-like aminotransferase	ADK19805	100.0

Input Sequence	ATG-E1_Chromosome_1909 # 1922354 # 1923556 # -1 # ID=1_1909;partial=00;start_type=GTG;rbs_motif=GGAG/GAGG;rbs_spacer=5-10bp;gc_cont=0.475						
	PROJECT ID	ACCESSION ID	ORGANISMS	CLASS	PROTEIN FUNCTION	PROTEIN ID	%IDENTITY
Matched Family	28537	CP001084	Lactobacillus casei str. Zhang, complete genome.	Lactobacillales	Aminodeoxychorismate lyase family	ADK18888	100.0

Input Sequence	ATG-E1_Chromosome_494 # 506062 # 507237 # 1 # ID=1_494;partial=00;start_type=ATG;rbs_motif=AGGA;rbs_spacer=5-10bp;gc_cont=0.401					
	PROJECT ID	ACCESSION ID	ORGANISMS	CLASS	PROTEIN FUNCTION	PROTEIN ID	%IDENTITY
Matched Family	30359	FM177140	Lactobacillus casei BL23 complete genome, strain BL23.	Lactobacillales	Mannitol-1-phosphate 5-dehydrogenase	CAQ65542	100.0

Input Sequence	ATG-E1_Chromosome_2292 # 2321643 # 2322827 # 1 # ID=1_2292;partial=00;start_type=TTG;rbs_motif=AGGA/GGAG/GAGG;rbs_spacer=11-12bp;gc_cont=0.478						
	PROJECT ID	ACCESSION ID	ORGANISMS	CLASS	PROTEIN FUNCTION	PROTEIN ID	%IDENTITY
Matched Family	28537	CP001084	Lactobacillus casei str. Zhang, complete genome.	Lactobacillales	conserved hypothetical protein	ADK19270	100.0

Input Sequence	ATG-E1_Chromosome_1539 # 1524028 # 1525197 # 1 # ID=1_1539;partial=00;start_type=ATG;rbs_motif=3Base/5BMM;rbs_spacer=13-15bp;gc_cont=0.466						
	PROJECT ID	ACCESSION ID	ORGANISMS	CLASS	PROTEIN FUNCTION	PROTEIN ID	%IDENTITY
Matched Family	30359	FM177140	Lactobacillus casei BL23 complete genome, strain BL23.	Lactobacillales	Cell division protein	CAQ66625	100.0

Input Sequence	ATG-E1_Chromosome_3051 # 3095323 # 3096495 # 1 # ID=1_3051;partial=00;start_type=ATG;rbs_motif=GGAGG;rbs_spacer=5-10bp;gc_cont=0.479						
	PROJECT ID	ACCESSION ID	ORGANISMS	CLASS	PROTEIN FUNCTION	PROTEIN ID	%IDENTITY
Matched Family	28537	CP001084	Lactobacillus casei str. Zhang, complete genome.	Lactobacillales	conserved hypothetical protein	ADK19994	100.0

Input Sequence	ATG-E1_Chromosome_193 # 201995 # 203158 # 1 # ID=1_193;partial=00;start_type=ATG;rbs_motif=GGAG/GAGG;rbs_spacer=5-10bp;gc_cont=0.478						
	PROJECT ID	ACCESSION ID	ORGANISMS	CLASS	PROTEIN FUNCTION	PROTEIN ID	%IDENTITY
Matched Family	28537	CP001084	Lactobacillus casei str. Zhang, complete genome.	Lactobacillales	conserved hypothetical protein	ADK17420	100.0

Input Sequence	ATG-E1_Chromosome_1219 # 1230097 # 1231248 # 1 # ID=1_1219;partial=00;start_type=ATG;rbs_motif=GGAG/GAGG;rbs_spacer=5-10bp;gc_cont=0.454						
	PROJECT ID	ACCESSION ID	ORGANISMS	CLASS	PROTEIN FUNCTION	PROTEIN ID	%IDENTITY
Matched Family	28537	CP001084	Lactobacillus casei str. Zhang, complete genome.	Lactobacillales	conserved hypothetical protein	ADK18277	100.0

Input Sequence	ATG-E1_Chromosome_2386 # 2428234 # 2429046 # -1 # ID=1_2386;partial=00;start_type=ATG;rbs_motif=None;rbs_spacer=None;gc_cont=0.438						
	PROJECT ID	ACCESSION ID	ORGANISMS	CLASS	PROTEIN FUNCTION	PROTEIN ID	%IDENTITY
Matched Family	28537	CP001084	Lactobacillus casei str. Zhang, complete genome.	Lactobacillales	conserved hypothetical protein	ADK19374	100.0

Input Sequence	ATG-E1_Chromosome_1660 # 1654670 # 1655818 # -1 # ID=1_1660;partial=00;start_type=ATG;rbs_motif=AGGAG;rbs_spacer=5-10bp;gc_cont=0.490						
	PROJECT ID	ACCESSION ID	ORGANISMS	CLASS	PROTEIN FUNCTION	PROTEIN ID	%IDENTITY
Matched Family	28537	CP001084	Lactobacillus casei str. Zhang, complete genome.	Lactobacillales	ATP phosphoribosyltransferase for histidine biosynthesis	ADK18656	100.0

Input Sequence	ATG-E1_Chromosome_1447 # 1428974 # 1430089 # -1 # ID=1_1447;partial=00;start_type=GTG;rbs_motif=GGA/GAG/AGG;rbs_spacer=11-12bp;gc_cont=0.438						
	PROJECT ID	ACCESSION ID	ORGANISMS	CLASS	PROTEIN FUNCTION	PROTEIN ID	%IDENTITY
Matched Family	30359	FM177140	Lactobacillus casei BL23 complete genome, strain BL23.	Lactobacillales	Histidine protein kinase; sensor protein	CAQ66525	100.0

Input Sequence	ATG-E1_Chromosome_3047 # 3089128 # 3090228 # 1 # ID=1_3047;partial=00;start_type=ATG;rbs_motif=GGAG/GAGG;rbs_spacer=5-10bp;gc_cont=0.462						
	PROJECT ID	ACCESSION ID	ORGANISMS	CLASS	PROTEIN FUNCTION	PROTEIN ID	%IDENTITY
Matched Family	28537	CP001084	Lactobacillus casei str. Zhang, complete genome.	Lactobacillales	Di- and tricarboxylate transporter	ADK19990	100.0

Input Sequence	ATG-E1_Chromosome_2321 # 2350821 # 2351918 # -1 # ID=1_2321;partial=00;start_type=TTG;rbs_motif=GGAGG;rbs_spacer=5-10bp;gc_cont=0.522

	PROJECT ID	ACCESSION ID	ORGANISMS	CLASS	PROTEIN FUNCTION	PROTEIN ID	%IDENTITY
**Matched Family**	30359	FM177140	Lactobacillus casei BL23 complete genome, strain BL23.	Lactobacillales	Predicted membrane protein	CAQ67378	100.0

**Input Sequence**	ATG-E1_Chromosome_225 # 238279 # 239352 # -1 # ID=1_225;partial=00;start_type=ATG;rbs_motif=GGAGG;rbs_spacer=5-10bp;gc_cont=0.497

	PROJECT ID	ACCESSION ID	ORGANISMS	CLASS	PROTEIN FUNCTION	PROTEIN ID	%IDENTITY
**Matched Family**	28537	CP001084	Lactobacillus casei str. Zhang, complete genome.	Lactobacillales	D-alanine-D-alanine ligase related ATP-grasp enzyme	ADK17452	100.0

**Input Sequence**	ATG-E1_Chromosome_2327 # 2356908 # 2357978 # 1 # ID=1_2327;partial=00;start_type=ATG;rbs_motif=GGAGG;rbs_spacer=5-10bp;gc_cont=0.486

	PROJECT ID	ACCESSION ID	ORGANISMS	CLASS	PROTEIN FUNCTION	PROTEIN ID	%IDENTITY
**Matched Family**	30359	FM177140	Lactobacillus casei BL23 complete genome, strain BL23.	Lactobacillales	Predicted membrane protein	CAQ67384	100.0

**Input Sequence**	ATG-E1_Chromosome_3023 # 3068284 # 3069351 # -1 # ID=1_3023;partial=00;start_type=ATG;rbs_motif=AGxAGG/AGGxGG;rbs_spacer=5-10bp;gc_cont=0.517

	PROJECT ID	ACCESSION ID	ORGANISMS	CLASS	PROTEIN FUNCTION	PROTEIN ID	%IDENTITY
**Matched Family**	402	CP000423	Lactobacillus casei ATCC 334, complete genome.	Lactobacillales	L-alanine-DL-glutamate epimerase related enzyme of enolase superfamily	ABJ71486	100.0

**Input Sequence**	ATG-E1_Chromosome_847 # 865248 # 866309 # -1 # ID=1_847;partial=00;start_type=ATG;rbs_motif=AGGAGG;rbs_spacer=5-10bp;gc_cont=0.458

	PROJECT ID	ACCESSION ID	ORGANISMS	CLASS	PROTEIN FUNCTION	PROTEIN ID	%IDENTITY
**Matched Family**	28537	CP001084	Lactobacillus casei str. Zhang, complete genome.	Lactobacillales	conserved hypothetical protein	ADK17921	100.0

**Input Sequence**	ATG-E1_Chromosome_542 # 554186 # 555247 # 1 # ID=1_542;partial=00;start_type=ATG;rbs_motif=AGGAG;rbs_spacer=5-10bp;gc_cont=0.484

	PROJECT ID	ACCESSION ID	ORGANISMS	CLASS	PROTEIN FUNCTION	PROTEIN ID	%IDENTITY
**Matched Family**	28537	CP001084	Lactobacillus casei str. Zhang, complete genome.	Lactobacillales	ABC-type antimicrobial peptide transport system, permease component	ADK17720	100.0

**Input Sequence**	ATG-E1_Chromosome_2428 # 2469571 # 2470629 # 1 # ID=1_2428;partial=00;start_type=ATG;rbs_motif=AGGAG;rbs_spacer=5-10bp;gc_cont=0.466

	PROJECT ID	ACCESSION ID	ORGANISMS	CLASS	PROTEIN FUNCTION	PROTEIN ID	%IDENTITY
**Matched Family**	28537	CP001084	Lactobacillus casei str. Zhang, complete genome.	Lactobacillales	Transcriptional regulator	ADK19419	100.0

**Input Sequence**	ATG-E1_Chromosome_1039 # 1053714 # 1054769 # 1 # ID=1_1039;partial=00;start_type=ATG;rbs_motif=GGAG/GAGG;rbs_spacer=5-10bp;gc_cont=0.494

	PROJECT ID	ACCESSION ID	ORGANISMS	CLASS	PROTEIN FUNCTION	PROTEIN ID	%IDENTITY
**Matched Family**	30359	FM177140	Lactobacillus casei BL23 complete genome, strain BL23.	Lactobacillales	Competence protein	CAQ66098	100.0

**Input Sequence**	ATG-E1_Chromosome_200 # 212873 # 213928 # 1 # ID=1_200;partial=00;start_type=ATG;rbs_motif=AGGAG;rbs_spacer=5-10bp;gc_cont=0.466

	PROJECT ID	ACCESSION ID	ORGANISMS	CLASS	PROTEIN FUNCTION	PROTEIN ID	%IDENTITY
**Matched Family**	28537	CP001084	Lactobacillus casei str. Zhang, complete genome.	Lactobacillales	metal-dependent hydrolase (putative)	ADK17427	100.0

**Input Sequence**	ATG-E1_Chromosome_1106 # 1124686 # 1125723 # 1 # ID=1_1106;partial=00;start_type=ATG;rbs_motif=AGxAGG/AGGxGG;rbs_spacer=5-10bp;gc_cont=0.490

	PROJECT ID	ACCESSION ID	ORGANISMS	CLASS	PROTEIN FUNCTION	PROTEIN ID	%IDENTITY
**Matched Family**	28537	CP001084	Lactobacillus casei str. Zhang, complete genome.	Lactobacillales	central glycolytic genes regulator	ADK18155	100.0

**Input Sequence**	ATG-E1_Chromosome_2414 # 2451613 # 2452647 # -1 # ID=1_2414;partial=00;start_type=ATG;rbs_motif=AGGAGG;rbs_spacer=5-10bp;gc_cont=0.507

	PROJECT ID	ACCESSION ID	ORGANISMS	CLASS	PROTEIN FUNCTION	PROTEIN ID	%IDENTITY
**Matched**	30359	FM177140	Lactobacillus casei BL23	Lactobacillales	Putative uncharacterized protein	CAQ67475	100.0

Family			complete genome, strain BL23.				

Input Sequence: ATG-E1_Chromosome_1003 # 1017518 # 1018531 # 1 # ID=1_1003;partial=00;start_type=ATG;rbs_motif=GGAG/GAGG;rbs_spacer=5-10bp;gc_cont=0.442

	PROJECT ID	ACCESSION ID	ORGANISMS	CLASS	PROTEIN FUNCTION	PROTEIN ID	%IDENTITY
Matched Family	28537	CP001084	Lactobacillus casei str. Zhang, complete genome.	Lactobacillales	Predicted integral membrane protein	ADK18061	100.0

Input Sequence: ATG-E1_Chromosome_1172 # 1191213 # 1192223 # 1 # ID=1_1172;partial=00;start_type=TTG;rbs_motif=AGxAGG/AGGxGG;rbs_spacer=5-10bp;gc_cont=0.469

	PROJECT ID	ACCESSION ID	ORGANISMS	CLASS	PROTEIN FUNCTION	PROTEIN ID	%IDENTITY
Matched Family	30359	FM177140	Lactobacillus casei BL23 complete genome, strain BL23.	Lactobacillales	Putative modification methylase LaaG	CAQ66279	100.0

Input Sequence: ATG-E1_Chromosome_419 # 429349 # 430356 # 1 # ID=1_419;partial=00;start_type=ATG;rbs_motif=GGA/GAG/AGG;rbs_spacer=11-12bp;gc_cont=0.478

	PROJECT ID	ACCESSION ID	ORGANISMS	CLASS	PROTEIN FUNCTION	PROTEIN ID	%IDENTITY
Matched Family	28537	CP001084	Lactobacillus casei str. Zhang, complete genome.	Lactobacillales	ribose operon repressor	ADK17647	100.0

Input Sequence: ATG-E1_Chromosome_1247 # 1260687 # 1261694 # 1 # ID=1_1247;partial=00;start_type=ATG;rbs_motif=AGGAGG;rbs_spacer=5-10bp;gc_cont=0.482

	PROJECT ID	ACCESSION ID	ORGANISMS	CLASS	PROTEIN FUNCTION	PROTEIN ID	%IDENTITY
Matched Family	28537	CP001084	Lactobacillus casei str. Zhang, complete genome.	Lactobacillales	Ribonuclease BN-like family enzyme	ADK18308	100.0

Input Sequence: ATG-E1_Chromosome_587 # 608136 # 609140 # 1 # ID=1_587;partial=00;start_type=ATG;rbs_motif=GGAG/GAGG;rbs_spacer=5-10bp;gc_cont=0.449

	PROJECT ID	ACCESSION ID	ORGANISMS	CLASS	PROTEIN FUNCTION	PROTEIN ID	%IDENTITY
Matched Family	28537	CP001084	Lactobacillus casei str. Zhang, complete genome.	Lactobacillales	Surface protein from Gram-positive cocci, anchor region	ADK17777	100.0

Input Sequence: ATG-E1_Chromosome_1916 # 1930379 # 1931377 # 1 # ID=1_1916;partial=00;start_type=ATG;rbs_motif=GGA/GAG/AGG;rbs_spacer=5-10bp;gc_cont=0.475

	PROJECT ID	ACCESSION ID	ORGANISMS	CLASS	PROTEIN FUNCTION	PROTEIN ID	%IDENTITY
Matched Family	30359	FM177140	Lactobacillus casei BL23 complete genome, strain BL23.	Lactobacillales	Membrane protein chaperone oxaA	CAQ66975	100.0

Input Sequence: ATG-E1_Chromosome_458 # 468573 # 469556 # -1 # ID=1_458;partial=00;start_type=TTG;rbs_motif=GGAG/GAGG;rbs_spacer=5-10bp;gc_cont=0.430

	PROJECT ID	ACCESSION ID	ORGANISMS	CLASS	PROTEIN FUNCTION	PROTEIN ID	%IDENTITY
Matched Family	30359	FM177140	Lactobacillus casei BL23 complete genome, strain BL23.	Lactobacillales	Galactose operon repressor	CAQ65495	100.0

Input Sequence: ATG-E1_Chromosome_1963 # 1972461 # 1973438 # -1 # ID=1_1963;partial=00;start_type=GTG;rbs_motif=GGA/GAG/AGG;rbs_spacer=5-10bp;gc_cont=0.438

	PROJECT ID	ACCESSION ID	ORGANISMS	CLASS	PROTEIN FUNCTION	PROTEIN ID	%IDENTITY
Matched Family	28537	CP001084	Lactobacillus casei str. Zhang, complete genome.	Lactobacillales	Predicted HD-superfamily hydrolase	ADK18940	100.0

Input Sequence: ATG-E1_Chromosome_763 # 771742 # 772716 # -1 # ID=1_763;partial=00;start_type=ATG;rbs_motif=AGGA;rbs_spacer=5-10bp;gc_cont=0.501

	PROJECT ID	ACCESSION ID	ORGANISMS	CLASS	PROTEIN FUNCTION	PROTEIN ID	%IDENTITY
Matched Family	28537	CP001084	Lactobacillus casei str. Zhang, complete genome.	Lactobacillales	permease of the drug/metabolite transporter (DMT) superfamily	ADK17821	100.0

Input Sequence: ATG-E1_Chromosome_1413 # 1398315 # 1399283 # 1 # ID=1_1413;partial=00;start_type=TTG;rbs_motif=AGGAGG;rbs_spacer=5-10bp;gc_cont=0.444

	PROJECT ID	ACCESSION ID	ORGANISMS	CLASS	PROTEIN FUNCTION	PROTEIN ID	%IDENTITY
Matched Family	30359	FM177140	Lactobacillus casei BL23 complete genome, strain BL23.	Lactobacillales	Putative uncharacterized protein	CAQ66490	100.0

Input Sequence	ATG-E1_Chromosome_1639 # 1635504 # 1636472 # 1 # ID=1_1639;partial=00;start_type=ATG;rbs_motif=GGA/GAG/AGG;rbs_spacer=5-10bp;gc_cont=0.441						
	PROJECT ID	ACCESSION ID	ORGANISMS	CLASS	PROTEIN FUNCTION	PROTEIN ID	%IDENTITY
Matched Family	28537	CP001084	Lactobacillus casei str. Zhang, complete genome.	Lactobacillales	Transcriptional regulator	ADK18632	100.0

Input Sequence	ATG-E1_Chromosome_2175 # 2187033 # 2187998 # -1 # ID=1_2175;partial=00;start_type=ATG;rbs_motif=GGAGG;rbs_spacer=5-10bp;gc_cont=0.497						
	PROJECT ID	ACCESSION ID	ORGANISMS	CLASS	PROTEIN FUNCTION	PROTEIN ID	%IDENTITY
Matched Family	402	CP000423	Lactobacillus casei ATCC 334, complete genome.	Lactobacillales	ferrochelatase	ABJ70749	100.0

Input Sequence	ATG-E1_Chromosome_2486 # 2528143 # 2529102 # -1 # ID=1_2486;partial=00;start_type=ATG;rbs_motif=GGxGG;rbs_spacer=5-10bp;gc_cont=0.478						
	PROJECT ID	ACCESSION ID	ORGANISMS	CLASS	PROTEIN FUNCTION	PROTEIN ID	%IDENTITY
Matched Family	28537	CP001084	Lactobacillus casei str. Zhang, complete genome.	Lactobacillales	1,4-dihydroxy-2-naphthoate octaprenyltransferase	ADK19473	100.0

Input Sequence	ATG-E1_Chromosome_852 # 872352 # 872549 # 1 # ID=1_852;partial=00;start_type=ATG;rbs_motif=None;rbs_spacer=None;gc_cont=0.439						
	PROJECT ID	ACCESSION ID	ORGANISMS	CLASS	PROTEIN FUNCTION	PROTEIN ID	%IDENTITY
Matched Family	28537	CP001084	Lactobacillus casei str. Zhang, complete genome.	Lactobacillales	ABC-type uncharacterized transport system, periplasmic component	ADK17927	100.0

Input Sequence	ATG-E1_Chromosome_2216 # 2236378 # 2237322 # -1 # ID=1_2216;partial=00;start_type=ATG;rbs_motif=AGGAGG;rbs_spacer=5-10bp;gc_cont=0.507						
	PROJECT ID	ACCESSION ID	ORGANISMS	CLASS	PROTEIN FUNCTION	PROTEIN ID	%IDENTITY
Matched Family	28537	CP001084	Lactobacillus casei str. Zhang, complete genome.	Lactobacillales	Predicted Co/Zn/Cd cation transporter	ADK19181	100.0

Input Sequence	ATG-E1_Chromosome_2828 # 2865884 # 2866825 # -1 # ID=1_2828;partial=00;start_type=ATG;rbs_motif=AGGAGG;rbs_spacer=5-10bp;gc_cont=0.493						
	PROJECT ID	ACCESSION ID	ORGANISMS	CLASS	PROTEIN FUNCTION	PROTEIN ID	%IDENTITY
Matched Family	28537	CP001084	Lactobacillus casei str. Zhang, complete genome.	Lactobacillales	Exopolyphosphatase	ADK19771	100.0

Input Sequence	ATG-E1_Chromosome_1699 # 1699891 # 1700826 # -1 # ID=1_1699;partial=00;start_type=ATG;rbs_motif=AGGAG;rbs_spacer=5-10bp;gc_cont=0.499						
	PROJECT ID	ACCESSION ID	ORGANISMS	CLASS	PROTEIN FUNCTION	PROTEIN ID	%IDENTITY
Matched Family	30359	FM177140	Lactobacillus casei BL23 complete genome, strain BL23.	Lactobacillales	D-3-phosphoglycerate dehydrogenase	CAQ66779	100.0

Input Sequence	ATG-E1_Chromosome_907 # 918287 # 919222 # 1 # ID=1_907;partial=00;start_type=ATG;rbs_motif=GGAG/GAGG;rbs_spacer=5-10bp;gc_cont=0.471						
	PROJECT ID	ACCESSION ID	ORGANISMS	CLASS	PROTEIN FUNCTION	PROTEIN ID	%IDENTITY
Matched Family	30359	FM177140	Lactobacillus casei BL23 complete genome, strain BL23.	Lactobacillales	Mg2+ and Co2+ transporter	CAQ65927	100.0

Input Sequence	ATG-E1_Chromosome_3026 # 3071192 # 3072124 # -1 # ID=1_3026;partial=00;start_type=ATG;rbs_motif=GGA/GAG/AGG;rbs_spacer=3-4bp;gc_cont=0.505						
	PROJECT ID	ACCESSION ID	ORGANISMS	CLASS	PROTEIN FUNCTION	PROTEIN ID	%IDENTITY
Matched Family	28537	CP001084	Lactobacillus casei str. Zhang, complete genome.	Lactobacillales	Transcriptional regulator	ADK19971	100.0

Input Sequence	ATG-E1_Chromosome_1060 # 1074262 # 1075191 # 1 # ID=1_1060;partial=00;start_type=ATG;rbs_motif=None;rbs_spacer=None;gc_cont=0.477						
	PROJECT ID	ACCESSION ID	ORGANISMS	CLASS	PROTEIN FUNCTION	PROTEIN ID	%IDENTITY
Matched Family	28537	CP001084	Lactobacillus casei str. Zhang, complete genome.	Lactobacillales	Xre-like DNA-binding protein	ADK18109	100.0

Input Sequence	ATG-E1_Chromosome_2218 # 2238007 # 2238936 # 1 # ID=1_2218;partial=00;start_type=ATG;rbs_motif=GGAGG;rbs_spacer=5-10bp;gc_cont=0.509						
	PROJECT ID	ACCESSION ID	ORGANISMS	CLASS	PROTEIN FUNCTION	PROTEIN ID	%IDENTITY
Matched Family	28537	CP001084	Lactobacillus casei str. Zhang, complete genome.	Lactobacillales	Thiamine biosynthesis membrane-associated lipoprotein	ADK19183	100.0

Input Sequence	ATG-E1_Chromosome_53 # 57121 # 58050 # 1 # ID=1_53;partial=00;start_type=ATG;rbs_motif=GGAGG;rbs_spacer=5-10bp;gc_cont=0.495						
	PROJECT ID	ACCESSION ID	ORGANISMS	CLASS	PROTEIN FUNCTION	PROTEIN ID	%IDENTITY
Matched Family	402	CP000423	Lactobacillus casei ATCC 334, complete genome.	Lactobacillales	hydrolase of the alpha/beta superfamily	ABJ71591	100.0

Input Sequence	ATG-E1_Chromosome_2823 # 2862607 # 2863533 # -1 # ID=1_2823;partial=00;start_type=ATG;rbs_motif=AGGAG;rbs_spacer=5-10bp;gc_cont=0.484						
	PROJECT ID	ACCESSION ID	ORGANISMS	CLASS	PROTEIN FUNCTION	PROTEIN ID	%IDENTITY
Matched Family	30359	FM177140	Lactobacillus casei BL23 complete genome, strain BL23.	Lactobacillales	Putative uncharacterized protein	CAQ67828	100.0

Input Sequence	ATG-E1_Chromosome_2400 # 2440530 # 2441450 # -1 # ID=1_2400;partial=00;start_type=GTG;rbs_motif=GGAG/GAGG;rbs_spacer=5-10bp;gc_cont=0.476						
	PROJECT ID	ACCESSION ID	ORGANISMS	CLASS	PROTEIN FUNCTION	PROTEIN ID	%IDENTITY
Matched Family	28537	CP001084	Lactobacillus casei str. Zhang, complete genome.	Lactobacillales	permease of the drug/metabolite transporter (DMT) superfamily	ADK19387	100.0

Input Sequence	ATG-E1_Chromosome_1744 # 1746648 # 1747568 # -1 # ID=1_1744;partial=00;start_type=ATG;rbs_motif=GGAG/GAGG;rbs_spacer=5-10bp;gc_cont=0.472						
	PROJECT ID	ACCESSION ID	ORGANISMS	CLASS	PROTEIN FUNCTION	PROTEIN ID	%IDENTITY
Matched Family	28537	CP001084	Lactobacillus casei str. Zhang, complete genome.	Lactobacillales	conserved hypothetical protein	ADK18743	100.0

Input Sequence	ATG-E1_Chromosome_67 # 74037 # 74957 # 1 # ID=1_67;partial=00;start_type=ATG;rbs_motif=AGxAGG/AGGxGG;rbs_spacer=5-10bp;gc_cont=0.471						
	PROJECT ID	ACCESSION ID	ORGANISMS	CLASS	PROTEIN FUNCTION	PROTEIN ID	%IDENTITY
Matched Family	32195	FM179322	Lactobacillus rhamnosus GG whole genome sequence, strain GG (ATCC 53103).	Lactobacillales	Transposase, IS30 family protein	CAR86359	100.0

Input Sequence	ATG-E1_Chromosome_2611 # 2650563 # 2651480 # 1 # ID=1_2611;partial=00;start_type=ATG;rbs_motif=AGGA/GGAG/GAGG;rbs_spacer=11-12bp;gc_cont=0.447						
	PROJECT ID	ACCESSION ID	ORGANISMS	CLASS	PROTEIN FUNCTION	PROTEIN ID	%IDENTITY
Matched Family	28537	CP001084	Lactobacillus casei str. Zhang, complete genome.	Lactobacillales	ABC-type metal ion transport system, periplasmic component/surface adhesin	ADK19600	100.0

Input Sequence	ATG-E1_Chromosome_1361 # 1350842 # 1351759 # 1 # ID=1_1361;partial=00;start_type=ATG;rbs_motif=GGA/GAG/AGG;rbs_spacer=5-10bp;gc_cont=0.487						
	PROJECT ID	ACCESSION ID	ORGANISMS	CLASS	PROTEIN FUNCTION	PROTEIN ID	%IDENTITY
Matched Family	28537	CP001084	Lactobacillus casei str. Zhang, complete genome.	Lactobacillales	Predicted dehydrogenase related protein	ADK18362	100.0

Input Sequence	ATG-E1_Chromosome_1552 # 1538833 # 1539744 # 1 # ID=1_1552;partial=00;start_type=ATG;rbs_motif=AGGAGG;rbs_spacer=5-10bp;gc_cont=0.477						
	PROJECT ID	ACCESSION ID	ORGANISMS	CLASS	PROTEIN FUNCTION	PROTEIN ID	%IDENTITY
Matched Family	30359	FM177140	Lactobacillus casei BL23 complete genome, strain BL23.	Lactobacillales	B	CAQ66638	100.0

Input Sequence	ATG-E1_Chromosome_2172 # 2184158 # 2185063 # 1 # ID=1_2172;partial=00;start_type=ATG;rbs_motif=GGA/GAG/AGG;rbs_spacer=5-10bp;gc_cont=0.472						
	PROJECT ID	ACCESSION ID	ORGANISMS	CLASS	PROTEIN FUNCTION	PROTEIN ID	%IDENTITY
Matched Family	30359	FM177140	Lactobacillus casei BL23 complete genome, strain BL23.	Lactobacillales	Prolinase	CAQ67229	100.0

Input	ATG-E1_Chromosome_365 # 375376 # 376278 # -1 # ID=1_365;partial=00;start_type=ATG;rbs_motif=GGxGG;rbs_spacer=5-10bp;gc_cont=0.476

Sequence	

	PROJECT ID	ACCESSION ID	ORGANISMS	CLASS	PROTEIN FUNCTION	PROTEIN ID	%IDENTITY
Matched Family	28537	CP001084	Lactobacillus casei str. Zhang, complete genome.	Lactobacillales	alpha/beta hydrolase superfamily protein	ADK17587	100.0

Input Sequence	ATG-E1_Chromosome_1962 # 1971363 # 1972265 # 1 # ID=1_1962;partial=00;start_type=ATG;rbs_motif=AGGA/GGAG/GAGG;rbs_spacer=11-12bp;gc_cont=0.426

	PROJECT ID	ACCESSION ID	ORGANISMS	CLASS	PROTEIN FUNCTION	PROTEIN ID	%IDENTITY
Matched Family	30359	FM177140	Lactobacillus casei BL23 complete genome, strain BL23.	Lactobacillales	Protein maturation protease (Peptidylprolyl isomerase)	CAQ67021	100.0

Input Sequence	ATG-E1_Chromosome_2616 # 2656120 # 2657022 # 1 # ID=1_2616;partial=00;start_type=ATG;rbs_motif=AGGAG;rbs_spacer=5-10bp;gc_cont=0.478

	PROJECT ID	ACCESSION ID	ORGANISMS	CLASS	PROTEIN FUNCTION	PROTEIN ID	%IDENTITY
Matched Family	28537	CP001084	Lactobacillus casei str. Zhang, complete genome.	Lactobacillales	ABC-type metal ion transport system, periplasmic component/surface adhesin	ADK19606	100.0

Input Sequence	ATG-E1_Chromosome_2749 # 2783995 # 2784894 # -1 # ID=1_2749;partial=00;start_type=ATG;rbs_motif=AGGAGG;rbs_spacer=5-10bp;gc_cont=0.490

	PROJECT ID	ACCESSION ID	ORGANISMS	CLASS	PROTEIN FUNCTION	PROTEIN ID	%IDENTITY
Matched Family	402	CP000423	Lactobacillus casei ATCC 334, complete genome.	Lactobacillales	hypothetical protein	ABJ71258	100.0

Input Sequence	ATG-E1_Chromosome_1431 # 1417139 # 1417783 # -1 # ID=1_1431;partial=00;start_type=ATG;rbs_motif=None;rbs_spacer=None;gc_cont=0.474

	PROJECT ID	ACCESSION ID	ORGANISMS	CLASS	PROTEIN FUNCTION	PROTEIN ID	%IDENTITY
Matched Family	402	CP000423	Lactobacillus casei ATCC 334, complete genome.	Lactobacillales	Signal transduction histidine kinase	ABJ69994	100.0

Input Sequence	ATG-E1_Chromosome_277 # 291379 # 292272 # 1 # ID=1_277;partial=00;start_type=ATG;rbs_motif=GGA/GAG/AGG;rbs_spacer=5-10bp;gc_cont=0.399

	PROJECT ID	ACCESSION ID	ORGANISMS	CLASS	PROTEIN FUNCTION	PROTEIN ID	%IDENTITY
Matched Family	28537	CP001084	Lactobacillus casei str. Zhang, complete genome.	Lactobacillales	Transcriptional regulator, xre family	ADK17504	100.0

Input Sequence	ATG-E1_Chromosome_601 # 636476 # 637369 # -1 # ID=1_601;partial=00;start_type=ATG;rbs_motif=AGGAGG;rbs_spacer=5-10bp;gc_cont=0.484

	PROJECT ID	ACCESSION ID	ORGANISMS	CLASS	PROTEIN FUNCTION	PROTEIN ID	%IDENTITY
Matched Family	402	CP000423	Lactobacillus casei ATCC 334, complete genome.	Lactobacillales	transcriptional regulator, LysR family	ABJ69324	100.0

Input Sequence	ATG-E1_Chromosome_1535 # 1519730 # 1520620 # 1 # ID=1_1535;partial=00;start_type=ATG;rbs_motif=AGGAG/GGAGG;rbs_spacer=11-12bp;gc_cont=0.457

	PROJECT ID	ACCESSION ID	ORGANISMS	CLASS	PROTEIN FUNCTION	PROTEIN ID	%IDENTITY
Matched Family	30359	FM177140	Lactobacillus casei BL23 complete genome, strain BL23.	Lactobacillales	L-lactate dehydrogenase (L-LDH)	CAQ66621	100.0

Input Sequence	ATG-E1_Chromosome_2430 # 2471720 # 2472607 # -1 # ID=1_2430;partial=00;start_type=ATG;rbs_motif=AGGAGG;rbs_spacer=5-10bp;gc_cont=0.482

	PROJECT ID	ACCESSION ID	ORGANISMS	CLASS	PROTEIN FUNCTION	PROTEIN ID	%IDENTITY
Matched Family	30359	FM177140	Lactobacillus casei BL23 complete genome, strain BL23.	Lactobacillales	ABC-type multidrug transport system, ATPase component	CAQ67491	100.0

Input Sequence	ATG-E1_Chromosome_1415 # 1400189 # 1401076 # 1 # ID=1_1415;partial=00;start_type=ATG;rbs_motif=GGAG/GAGG;rbs_spacer=5-10bp;gc_cont=0.439

	PROJECT ID	ACCESSION ID	ORGANISMS	CLASS	PROTEIN FUNCTION	PROTEIN ID	%IDENTITY
Matched Family	28537	CP001084	Lactobacillus casei str. Zhang, complete genome.	Lactobacillales	Transcriptional regulator, xre family	ADK18418	100.0

Input Sequence	ATG-E1_Chromosome_1695 # 1695128 # 1696009 # -1 # ID=1_1695;partial=00;start_type=ATG;rbs_motif=AGxAGG/AGGxGG;rbs_spacer=5-10bp;gc_cont=0.490

	PROJECT ID	ACCESSION ID	ORGANISMS	CLASS	PROTEIN FUNCTION	PROTEIN ID	%IDENTITY

Matched Family	28537	CP001084	Lactobacillus casei str. Zhang, complete genome.	Lactobacillales	fhu operon transcription regulator	ADK18694	100.0

Input Sequence: ATG-E1_Chromosome_2786 # 2827419 # 2828300 # -1 # ID=1_2786;partial=00;start_type=ATG;rbs_motif=AGxAGG/AGGxGG;rbs_spacer=5-10bp;gc_cont=0.432

	PROJECT ID	ACCESSION ID	ORGANISMS	CLASS	PROTEIN FUNCTION	PROTEIN ID	%IDENTITY
Matched Family	28537	CP001084	Lactobacillus casei str. Zhang, complete genome.	Lactobacillales	Predicted integral membrane protein	ADK19728	100.0

Input Sequence: ATG-E1_Chromosome_2802 # 2842128 # 2843006 # -1 # ID=1_2802;partial=00;start_type=ATG;rbs_motif=GGAGG;rbs_spacer=5-10bp;gc_cont=0.514

	PROJECT ID	ACCESSION ID	ORGANISMS	CLASS	PROTEIN FUNCTION	PROTEIN ID	%IDENTITY
Matched Family	28537	CP001084	Lactobacillus casei str. Zhang, complete genome.	Lactobacillales	Co/Zn/Cd efflux system component	ADK19745	100.0

Input Sequence: ATG-E1_Chromosome_2535 # 2575908 # 2576786 # 1 # ID=1_2535;partial=00;start_type=ATG;rbs_motif=AGGAGG;rbs_spacer=5-10bp;gc_cont=0.469

	PROJECT ID	ACCESSION ID	ORGANISMS	CLASS	PROTEIN FUNCTION	PROTEIN ID	%IDENTITY
Matched Family	30359	FM177140	Lactobacillus casei BL23 complete genome, strain BL23.	Lactobacillales	Sodium ABC tranporter ATP-binding protein NatA	CAQ67583	100.0

Input Sequence: ATG-E1_Chromosome_2298 # 2330311 # 2331186 # -1 # ID=1_2298;partial=00;start_type=TTG;rbs_motif=AGGAG;rbs_spacer=5-10bp;gc_cont=0.494

	PROJECT ID	ACCESSION ID	ORGANISMS	CLASS	PROTEIN FUNCTION	PROTEIN ID	%IDENTITY
Matched Family	28537	CP001084	Lactobacillus casei str. Zhang, complete genome.	Lactobacillales	Predicted nucleoside-diphosphate-sugar epimerase	ADK19276	100.0

Input Sequence: ATG-E1_Chromosome_139 # 146514 # 147305 # -1 # ID=1_139;partial=00;start_type=ATG;rbs_motif=GGA/GAG/AGG;rbs_spacer=5-10bp;gc_cont=0.438

	PROJECT ID	ACCESSION ID	ORGANISMS	CLASS	PROTEIN FUNCTION	PROTEIN ID	%IDENTITY
Matched Family	28537	CP001084	Lactobacillus casei str. Zhang, complete genome.	Lactobacillales	Predicted metal-dependent membrane protease	ADK17355	100.0

Input Sequence: ATG-E1_Chromosome_1751 # 1754203 # 1755078 # -1 # ID=1_1751;partial=00;start_type=ATG;rbs_motif=AGGAGG;rbs_spacer=5-10bp;gc_cont=0.478

	PROJECT ID	ACCESSION ID	ORGANISMS	CLASS	PROTEIN FUNCTION	PROTEIN ID	%IDENTITY
Matched Family	28537	CP001084	Lactobacillus casei str. Zhang, complete genome.	Lactobacillales	ABC-type polar amino acid transport system, ATPase component	ADK18749	100.0

Input Sequence: ATG-E1_Chromosome_10 # 8351 # 9226 # -1 # ID=1_10;partial=00;start_type=ATG;rbs_motif=GGA/GAG/AGG;rbs_spacer=11-12bp;gc_cont=0.475

	PROJECT ID	ACCESSION ID	ORGANISMS	CLASS	PROTEIN FUNCTION	PROTEIN ID	%IDENTITY
Matched Family	28537	CP001084	Lactobacillus casei str. Zhang, complete genome.	Lactobacillales	Transcriptional regulator, xre family	ADK20033	100.0

Input Sequence: ATG-E1_Chromosome_1178 # 1197181 # 1198050 # 1 # ID=1_1178;partial=00;start_type=ATG;rbs_motif=GGxGG;rbs_spacer=3-4bp;gc_cont=0.478

	PROJECT ID	ACCESSION ID	ORGANISMS	CLASS	PROTEIN FUNCTION	PROTEIN ID	%IDENTITY
Matched Family	28537	CP001084	Lactobacillus casei str. Zhang, complete genome.	Lactobacillales	1-acyl-sn-glycerol-3-phosphate acyltransferase	ADK18234	100.0

Input Sequence: ATG-E1_Chromosome_2361 # 2395216 # 2396082 # 1 # ID=1_2361;partial=00;start_type=ATG;rbs_motif=AGGAG/GGAGG;rbs_spacer=11-12bp;gc_cont=0.468

	PROJECT ID	ACCESSION ID	ORGANISMS	CLASS	PROTEIN FUNCTION	PROTEIN ID	%IDENTITY
Matched Family	30359	FM177140	Lactobacillus casei BL23 complete genome, strain BL23.	Lactobacillales	ABC-type multidrug transport system, ATPase component	CAQ67420	100.0

Input Sequence: ATG-E1_Chromosome_1165 # 1187513 # 1188379 # 1 # ID=1_1165;partial=00;start_type=ATG;rbs_motif=GGA/GAG/AGG;rbs_spacer=5-10bp;gc_cont=0.507

	PROJECT ID	ACCESSION ID	ORGANISMS	CLASS	PROTEIN FUNCTION	PROTEIN ID	%IDENTITY
Matched Family	28537	CP001084	Lactobacillus casei str. Zhang, complete genome.	Lactobacillales	Type II secretory pathway/competence component, ATPase	ADK18221	100.0

Input Sequence: ATG-E1_Chromosome_445 # 454453 # 455313 # 1 # ID=1_445;partial=00;start_type=ATG;rbs_motif=GGAGG;rbs_spacer=5-10bp;gc_cont=0.463

	PROJECT ID	ACCESSION ID	ORGANISMS	CLASS	PROTEIN FUNCTION	PROTEIN ID	%IDENTITY
**Matched Family**	28537	CP001084	Lactobacillus casei str. Zhang, complete genome.	Lactobacillales	Fructose/tagatose bisphosphate aldolase	ADK17673	100.0

Input Sequence: ATG-E1_Chromosome_1476 # 1463187 # 1464047 # 1 # ID=1_1476;partial=00;start_type=ATG;rbs_motif=GGAGG;rbs_spacer=5-10bp;gc_cont=0.475

	PROJECT ID	ACCESSION ID	ORGANISMS	CLASS	PROTEIN FUNCTION	PROTEIN ID	%IDENTITY
**Matched Family**	28537	CP001084	Lactobacillus casei str. Zhang, complete genome.	Lactobacillales	Cell shape-determining protein	ADK18480	100.0

Input Sequence: ATG-E1_Chromosome_375 # 386803 # 387651 # -1 # ID=1_375;partial=00;start_type=ATG;rbs_motif=GGA/GAG/AGG;rbs_spacer=5-10bp;gc_cont=0.519

	PROJECT ID	ACCESSION ID	ORGANISMS	CLASS	PROTEIN FUNCTION	PROTEIN ID	%IDENTITY
**Matched Family**	28537	CP001084	Lactobacillus casei str. Zhang, complete genome.	Lactobacillales	conserved hypothetical protein	ADK17599	100.0

Input Sequence: ATG-E1_Chromosome_9 # 7506 # 8354 # -1 # ID=1_9;partial=00;start_type=ATG;rbs_motif=AGGAGG;rbs_spacer=5-10bp;gc_cont=0.444

	PROJECT ID	ACCESSION ID	ORGANISMS	CLASS	PROTEIN FUNCTION	PROTEIN ID	%IDENTITY
**Matched Family**	28537	CP001084	Lactobacillus casei str. Zhang, complete genome.	Lactobacillales	Transcriptional regulator, xre family	ADK20032	100.0

Input Sequence: ATG-E1_Chromosome_929 # 939643 # 940488 # 1 # ID=1_929;partial=00;start_type=ATG;rbs_motif=AGGAG;rbs_spacer=5-10bp;gc_cont=0.479

	PROJECT ID	ACCESSION ID	ORGANISMS	CLASS	PROTEIN FUNCTION	PROTEIN ID	%IDENTITY
**Matched Family**	28537	CP001084	Lactobacillus casei str. Zhang, complete genome.	Lactobacillales	alpha/beta hydrolase superfamily protein	ADK17989	100.0

Input Sequence: ATG-E1_Chromosome_1610 # 1601733 # 1602578 # 1 # ID=1_1610;partial=00;start_type=ATG;rbs_motif=AGGAG;rbs_spacer=5-10bp;gc_cont=0.452

	PROJECT ID	ACCESSION ID	ORGANISMS	CLASS	PROTEIN FUNCTION	PROTEIN ID	%IDENTITY
**Matched Family**	28537	CP001084	Lactobacillus casei str. Zhang, complete genome.	Lactobacillales	Lysophospholipase L1 related esterase	ADK18611	100.0

Input Sequence: ATG-E1_Chromosome_1053 # 1066250 # 1067092 # 1 # ID=1_1053;partial=00;start_type=ATG;rbs_motif=AGGAGG;rbs_spacer=5-10bp;gc_cont=0.511

	PROJECT ID	ACCESSION ID	ORGANISMS	CLASS	PROTEIN FUNCTION	PROTEIN ID	%IDENTITY
**Matched Family**	402	CP000423	Lactobacillus casei ATCC 334, complete genome.	Lactobacillales	SAM-dependent methyltransferase	ABJ69712	100.0

Input Sequence: ATG-E1_Chromosome_1966 # 1976295 # 1977128 # 1 # ID=1_1966;partial=00;start_type=TTG;rbs_motif=GGAG/GAGG;rbs_spacer=5-10bp;gc_cont=0.492

	PROJECT ID	ACCESSION ID	ORGANISMS	CLASS	PROTEIN FUNCTION	PROTEIN ID	%IDENTITY
**Matched Family**	30359	FM177140	Lactobacillus casei BL23 complete genome, strain BL23.	Lactobacillales	Pseudouridine synthase, 23S-rRNA-specific	CAQ67025	100.0

Input Sequence: ATG-E1_Chromosome_1379 # 1370903 # 1371733 # 1 # ID=1_1379;partial=00;start_type=ATG;rbs_motif=GGA/GAG/AGG;rbs_spacer=5-10bp;gc_cont=0.508

	PROJECT ID	ACCESSION ID	ORGANISMS	CLASS	PROTEIN FUNCTION	PROTEIN ID	%IDENTITY
**Matched Family**	28537	CP001084	Lactobacillus casei str. Zhang, complete genome.	Lactobacillales	Methylase of polypeptide chain release factor	ADK18383	100.0

Input Sequence: ATG-E1_Chromosome_2515 # 2554699 # 2555529 # 1 # ID=1_2515;partial=00;start_type=ATG;rbs_motif=AGGAG;rbs_spacer=5-10bp;gc_cont=0.468

	PROJECT ID	ACCESSION ID	ORGANISMS	CLASS	PROTEIN FUNCTION	PROTEIN ID	%IDENTITY
**Matched Family**	30359	FM177140	Lactobacillus casei BL23 complete genome, strain BL23.	Lactobacillales	Transcriptional regulator	CAQ67561	100.0

Input Sequence	ATG-E1_Chromosome_304 # 312052 # 312882 # 1 # ID=1_304;partial=00;start_type=GTG;rbs_motif=GGAGG;rbs_spacer=5-10bp;gc_cont=0.480					
	PROJECT ID	ACCESSION ID	ORGANISMS	CLASS	PROTEIN FUNCTION	PROTEIN ID	%IDENTITY
Matched Family	28537	CP001084	Lactobacillus casei str. Zhang, complete genome.	Lactobacillales	Spo0J-like protein, ParB-like nuclease domain	ADK17531	100.0

Input Sequence	ATG-E1_Chromosome_447 # 456355 # 457185 # 1 # ID=1_447;partial=00;start_type=GTG;rbs_motif=GGAG/GAGG;rbs_spacer=5-10bp;gc_cont=0.420						
	PROJECT ID	ACCESSION ID	ORGANISMS	CLASS	PROTEIN FUNCTION	PROTEIN ID	%IDENTITY
Matched Family	30359	FM177140	Lactobacillus casei BL23 complete genome, strain BL23.	Lactobacillales	EF0126 (Putative uncharacterized protein)	CAQ65478	100.0

Input Sequence	ATG-E1_Chromosome_1249 # 1262492 # 1263316 # 1 # ID=1_1249;partial=00;start_type=TTG;rbs_motif=GGAGG;rbs_spacer=5-10bp;gc_cont=0.487						
	PROJECT ID	ACCESSION ID	ORGANISMS	CLASS	PROTEIN FUNCTION	PROTEIN ID	%IDENTITY
Matched Family	28537	CP001084	Lactobacillus casei str. Zhang, complete genome.	Lactobacillales	methionine aminopeptidase	ADK18310	100.0

Input Sequence	ATG-E1_Chromosome_2832 # 2871996 # 2872820 # 1 # ID=1_2832;partial=00;start_type=ATG;rbs_motif=AGGA;rbs_spacer=5-10bp;gc_cont=0.521						
	PROJECT ID	ACCESSION ID	ORGANISMS	CLASS	PROTEIN FUNCTION	PROTEIN ID	%IDENTITY
Matched Family	30359	FM177140	Lactobacillus casei BL23 complete genome, strain BL23.	Lactobacillales	SAM-dependent methyltransferase	CAQ67836	100.0

Input Sequence	ATG-E1_Chromosome_1027 # 1043168 # 1043989 # -1 # ID=1_1027;partial=00;start_type=ATG;rbs_motif=AGxAGG/AGGxGG;rbs_spacer=5-10bp;gc_cont=0.527						
	PROJECT ID	ACCESSION ID	ORGANISMS	CLASS	PROTEIN FUNCTION	PROTEIN ID	%IDENTITY
Matched Family	30359	FM177140	Lactobacillus casei BL23 complete genome, strain BL23.	Lactobacillales	Predicted sugar kinase	CAQ66039	100.0

Input Sequence	ATG-E1_Chromosome_846 # 864253 # 865074 # -1 # ID=1_846;partial=00;start_type=GTG;rbs_motif=AGGAGG;rbs_spacer=5-10bp;gc_cont=0.491						
	PROJECT ID	ACCESSION ID	ORGANISMS	CLASS	PROTEIN FUNCTION	PROTEIN ID	%IDENTITY
Matched Family	28537	CP001084	Lactobacillus casei str. Zhang, complete genome.	Lactobacillales	Hydroxymethylpyrimidine/phosphomethylpyrimidine kinase	ADK17920	100.0

Input Sequence	ATG-E1_Chromosome_2955 # 3007761 # 3008582 # 1 # ID=1_2955;partial=00;start_type=ATG;rbs_motif=AGGA;rbs_spacer=5-10bp;gc_cont=0.429						
	PROJECT ID	ACCESSION ID	ORGANISMS	CLASS	PROTEIN FUNCTION	PROTEIN ID	%IDENTITY
Matched Family	28537	CP001084	Lactobacillus casei str. Zhang, complete genome.	Lactobacillales	Predicted hydrolase of the HAD superfamily	ADK19919	100.0

Input Sequence	ATG-E1_Chromosome_2030 # 2041101 # 2041919 # 1 # ID=1_2030;partial=00;start_type=ATG;rbs_motif=AGGA/GGAG/GAGG;rbs_spacer=11-12bp;gc_cont=0.504						
	PROJECT ID	ACCESSION ID	ORGANISMS	CLASS	PROTEIN FUNCTION	PROTEIN ID	%IDENTITY
Matched Family	30359	FM177140	Lactobacillus casei BL23 complete genome, strain BL23.	Lactobacillales	Mro protein	CAQ67087	100.0

Input Sequence	ATG-E1_Chromosome_1529 # 1513238 # 1514053 # 1 # ID=1_1529;partial=00;start_type=ATG;rbs_motif=AGGAG;rbs_spacer=5-10bp;gc_cont=0.513						
	PROJECT ID	ACCESSION ID	ORGANISMS	CLASS	PROTEIN FUNCTION	PROTEIN ID	%IDENTITY
Matched Family	30359	FM177140	Lactobacillus casei BL23 complete genome, strain BL23.	Lactobacillales	Putative uncharacterized protein	CAQ66616	100.0

Input Sequence	ATG-E1_Chromosome_1483 # 1468016 # 1468828 # 1 # ID=1_1483;partial=00;start_type=ATG;rbs_motif=AGxAGG/AGGxGG;rbs_spacer=5-10bp;gc_cont=0.442						
	PROJECT ID	ACCESSION ID	ORGANISMS	CLASS	PROTEIN FUNCTION	PROTEIN ID	%IDENTITY
Matched Family	28537	CP001084	Lactobacillus casei str. Zhang, complete genome.	Lactobacillales	ABC-type amino acid transport/signal transduction system, periplasmic component/domain	ADK18487	100.0

Input Sequence	ATG-E1_Chromosome_2851 # 2896311 # 2897123 # -1 # ID=1_2851;partial=00;start_type=ATG;rbs_motif=AGGA;rbs_spacer=5-10bp;gc_cont=0.487

	PROJECT ID	ACCESSION ID	ORGANISMS	CLASS	PROTEIN FUNCTION	PROTEIN ID	%IDENTITY
**Matched Family**	28537	CP001084	Lactobacillus casei str. Zhang, complete genome.	Lactobacillales	Predicted hydrolase of the HAD superfamily	ADK19797	100.0

**Input Sequence**	ATG-E1_Chromosome_1429 # 1414190 # 1415002 # 1 # ID=1_1429;partial=00;start_type=GTG;rbs_motif=AGGAGG;rbs_spacer=5-10bp;gc_cont=0.469

	PROJECT ID	ACCESSION ID	ORGANISMS	CLASS	PROTEIN FUNCTION	PROTEIN ID	%IDENTITY
**Matched Family**	402	CP000423	Lactobacillus casei ATCC 334, complete genome.	Lactobacillales	Histidinol phosphatase related hydrolase of the PHP family	ABJ69992	100.0

**Input Sequence**	ATG-E1_Chromosome_113 # 121906 # 122712 # 1 # ID=1_113;partial=00;start_type=ATG;rbs_motif=GGA/GAG/AGG;rbs_spacer=5-10bp;gc_cont=0.468

	PROJECT ID	ACCESSION ID	ORGANISMS	CLASS	PROTEIN FUNCTION	PROTEIN ID	%IDENTITY
**Matched Family**	30359	FM177140	Lactobacillus casei BL23 complete genome, strain BL23.	Lactobacillales	Hydrolase Cof	CAQ65159	100.0

**Input Sequence**	ATG-E1_Chromosome_2575 # 2618226 # 2619032 # 1 # ID=1_2575;partial=00;start_type=ATG;rbs_motif=GGAG/GAGG;rbs_spacer=5-10bp;gc_cont=0.416

	PROJECT ID	ACCESSION ID	ORGANISMS	CLASS	PROTEIN FUNCTION	PROTEIN ID	%IDENTITY
**Matched Family**	30359	FM177140	Lactobacillus casei BL23 complete genome, strain BL23.	Lactobacillales	Response regulator	CAQ67628	100.0

**Input Sequence**	ATG-E1_Chromosome_337 # 346538 # 347335 # 1 # ID=1_337;partial=00;start_type=ATG;rbs_motif=None;rbs_spacer=None;gc_cont=0.467

	PROJECT ID	ACCESSION ID	ORGANISMS	CLASS	PROTEIN FUNCTION	PROTEIN ID	%IDENTITY
**Matched Family**	402	CP000423	Lactobacillus casei ATCC 334, complete genome.	Lactobacillales	ABC-type Mn2+/Zn2+ transport system, permease component	ABJ69106	100.0

**Input Sequence**	ATG-E1_Chromosome_3057 # 3103220 # 3104017 # -1 # ID=1_3057;partial=00;start_type=GTG;rbs_motif=GGAGG;rbs_spacer=5-10bp;gc_cont=0.462

	PROJECT ID	ACCESSION ID	ORGANISMS	CLASS	PROTEIN FUNCTION	PROTEIN ID	%IDENTITY
**Matched Family**	28537	CP001084	Lactobacillus casei str. Zhang, complete genome.	Lactobacillales	conserved hypothetical protein	ADK20002	100.0

**Input Sequence**	ATG-E1_Chromosome_1725 # 1730026 # 1730820 # -1 # ID=1_1725;partial=00;start_type=ATG;rbs_motif=AGGAGG;rbs_spacer=11-12bp;gc_cont=0.486

	PROJECT ID	ACCESSION ID	ORGANISMS	CLASS	PROTEIN FUNCTION	PROTEIN ID	%IDENTITY
**Matched Family**	30359	FM177140	Lactobacillus casei BL23 complete genome, strain BL23.	Lactobacillales	Putative uncharacterized protein	CAQ66802	100.0

**Input Sequence**	ATG-E1_Chromosome_1537 # 1520894 # 1521685 # 1 # ID=1_1537;partial=00;start_type=ATG;rbs_motif=GGA/GAG/AGG;rbs_spacer=5-10bp;gc_cont=0.486

	PROJECT ID	ACCESSION ID	ORGANISMS	CLASS	PROTEIN FUNCTION	PROTEIN ID	%IDENTITY
**Matched Family**	28537	CP001084	Lactobacillus casei str. Zhang, complete genome.	Lactobacillales	Archaeal fructose-1,6-bisphosphatase related enzyme of inositol monophosphatase family	ADK18539	100.0

**Input Sequence**	ATG-E1_Chromosome_1828 # 1846651 # 1846761 # 1 # ID=1_1828;partial=00;start_type=ATG;rbs_motif=AGGA/GGAG/GAGG;rbs_spacer=11-12bp;gc_cont=0.396

	PROJECT ID	ACCESSION ID	ORGANISMS	CLASS	PROTEIN FUNCTION	PROTEIN ID	%IDENTITY
**Matched Family**	28537	CP001084	Lactobacillus casei str. Zhang, complete genome.	Lactobacillales	Predicted membrane protein	ADK18810	100.0

**Input Sequence**	ATG-E1_Chromosome_2319 # 2349272 # 2349616 # -1 # ID=1_2319;partial=00;start_type=ATG;rbs_motif=AGxAGG/AGGxGG;rbs_spacer=5-10bp;gc_cont=0.507

	PROJECT ID	ACCESSION ID	ORGANISMS	CLASS	PROTEIN FUNCTION	PROTEIN ID	%IDENTITY
**Matched Family**	28537	CP001084	Lactobacillus casei str. Zhang, complete genome.	Lactobacillales	conserved hypothetical protein	ADK19298	100.0

**Input Sequence**	ATG-E1_Chromosome_2562 # 2607129 # 2607917 # 1 # ID=1_2562;partial=00;start_type=ATG;rbs_motif=AGGAGG;rbs_spacer=5-10bp;gc_cont=0.459

	PROJECT ID	ACCESSION ID	ORGANISMS	CLASS	PROTEIN FUNCTION	PROTEIN ID	%IDENTITY
**Matched**	402	CP000423	Lactobacillus casei ATCC 334,	Lactobacillales	hypothetical protein	ABJ71114	100.0

Family			complete genome.				

Input Sequence	ATG-E1_Chromosome_60 # 66157 # 66942 # 1 # ID=1_60;partial=00;start_type=ATG;rbs_motif=GGAGG;rbs_spacer=5-10bp;gc_cont=0.471						
	PROJECT ID	ACCESSION ID	ORGANISMS	CLASS	PROTEIN FUNCTION	PROTEIN ID	%IDENTITY
Matched Family	28537	CP001084	Lactobacillus casei str. Zhang, complete genome.	Lactobacillales	Predicted hydrolase of the HAD superfamily	ADK20081	100.0

Input Sequence	ATG-E1_Chromosome_345 # 356297 # 357079 # 1 # ID=1_345;partial=00;start_type=ATG;rbs_motif=AGGAGG;rbs_spacer=5-10bp;gc_cont=0.475						
	PROJECT ID	ACCESSION ID	ORGANISMS	CLASS	PROTEIN FUNCTION	PROTEIN ID	%IDENTITY
Matched Family	28537	CP001084	Lactobacillus casei str. Zhang, complete genome.	Lactobacillales	Glycosyltransferase related enzyme	ADK17571	100.0

Input Sequence	ATG-E1_Chromosome_450 # 459637 # 460416 # 1 # ID=1_450;partial=00;start_type=ATG;rbs_motif=AGGAGG;rbs_spacer=5-10bp;gc_cont=0.399						
	PROJECT ID	ACCESSION ID	ORGANISMS	CLASS	PROTEIN FUNCTION	PROTEIN ID	%IDENTITY
Matched Family	30359	FM177140	Lactobacillus casei BL23 complete genome, strain BL23.	Lactobacillales	Esterase C	CAQ65481	100.0

Input Sequence	ATG-E1_Chromosome_2957 # 3009743 # 3010516 # -1 # ID=1_2957;partial=00;start_type=GTG;rbs_motif=GGAG/GAGG;rbs_spacer=5-10bp;gc_cont=0.481						
	PROJECT ID	ACCESSION ID	ORGANISMS	CLASS	PROTEIN FUNCTION	PROTEIN ID	%IDENTITY
Matched Family	30359	FM177140	Lactobacillus casei BL23 complete genome, strain BL23.	Lactobacillales	Transcriptional regulator of sugar metabolism	CAQ67960	100.0

Input Sequence	ATG-E1_Chromosome_2987 # 3039101 # 3039868 # -1 # ID=1_2987;partial=00;start_type=ATG;rbs_motif=AGGAGG;rbs_spacer=5-10bp;gc_cont=0.474						
	PROJECT ID	ACCESSION ID	ORGANISMS	CLASS	PROTEIN FUNCTION	PROTEIN ID	%IDENTITY
Matched Family	30359	FM177140	Lactobacillus casei BL23 complete genome, strain BL23.	Lactobacillales	C component PTS system mannose-specific enzyme II	CAQ67989	100.0

Input Sequence	ATG-E1_Chromosome_2234 # 2260951 # 2261715 # -1 # ID=1_2234;partial=00;start_type=ATG;rbs_motif=GGA/GAG/AGG;rbs_spacer=5-10bp;gc_cont=0.486						
	PROJECT ID	ACCESSION ID	ORGANISMS	CLASS	PROTEIN FUNCTION	PROTEIN ID	%IDENTITY
Matched Family	402	CP000423	Lactobacillus casei ATCC 334, complete genome.	Lactobacillales	Capsular polysaccharide biosynthesis protein	ABJ70804	100.0

Input Sequence	ATG-E1_Chromosome_1448 # 1430079 # 1430843 # -1 # ID=1_1448;partial=00;start_type=ATG;rbs_motif=AGGAG;rbs_spacer=5-10bp;gc_cont=0.433						
	PROJECT ID	ACCESSION ID	ORGANISMS	CLASS	PROTEIN FUNCTION	PROTEIN ID	%IDENTITY
Matched Family	30359	FM177140	Lactobacillus casei BL23 complete genome, strain BL23.	Lactobacillales	ABC-type multidrug transport system, permease component	CAQ66526	100.0

Input Sequence	ATG-E1_Chromosome_77 # 87768 # 88532 # -1 # ID=1_77;partial=00;start_type=ATG;rbs_motif=AGGAG;rbs_spacer=5-10bp;gc_cont=0.492						
	PROJECT ID	ACCESSION ID	ORGANISMS	CLASS	PROTEIN FUNCTION	PROTEIN ID	%IDENTITY
Matched Family	28537	CP001084	Lactobacillus casei str. Zhang, complete genome.	Lactobacillales	conserved hypothetical protein	ADK20092	100.0

Input Sequence	ATG-E1_Chromosome_82 # 93008 # 93772 # -1 # ID=1_82;partial=00;start_type=ATG;rbs_motif=GGAGG;rbs_spacer=5-10bp;gc_cont=0.478						
	PROJECT ID	ACCESSION ID	ORGANISMS	CLASS	PROTEIN FUNCTION	PROTEIN ID	%IDENTITY
Matched Family	402	CP000423	Lactobacillus casei ATCC 334, complete genome.	Lactobacillales	Predicted RNA-binding protein	ABJ71614	100.0

Input Sequence	ATG-E1_Chromosome_2968 # 3019718 # 3020479 # -1 # ID=1_2968;partial=00;start_type=ATG;rbs_motif=GGAGG;rbs_spacer=5-10bp;gc_cont=0.462						
	PROJECT ID	ACCESSION ID	ORGANISMS	CLASS	PROTEIN FUNCTION	PROTEIN ID	%IDENTITY
Matched Family	30359	FM177140	Lactobacillus casei BL23 complete genome, strain BL23.	Lactobacillales	C component PTS system mannose-specific enzyme II	CAQ67972	100.0

Input Sequence	ATG-E1_Chromosome_526 # 539188 # 539949 # -1 # ID=1_526;partial=00;start_type=ATG;rbs_motif=AGxAGG/AGGxGG;rbs_spacer=5-10bp;gc_cont=0.455					
	PROJECT ID	ACCESSION ID	ORGANISMS	CLASS	PROTEIN FUNCTION	PROTEIN ID	%IDENTITY
Matched Family	28537	CP001084	Lactobacillus casei str. Zhang, complete genome.	Lactobacillales	transcriptional regulator, DeoR family	ADK17703	100.0

Input Sequence	ATG-E1_Chromosome_2972 # 3022789 # 3023550 # 1 # ID=1_2972;partial=00;start_type=ATG;rbs_motif=AGxAGG/AGGxGG;rbs_spacer=5-10bp;gc_cont=0.449						
	PROJECT ID	ACCESSION ID	ORGANISMS	CLASS	PROTEIN FUNCTION	PROTEIN ID	%IDENTITY
Matched Family	30359	FM177140	Lactobacillus casei BL23 complete genome, strain BL23.	Lactobacillales	Nicotinic acid mononucleotide adenyltransferase	CAQ67976	100.0

Input Sequence	ATG-E1_Chromosome_1109 # 1128238 # 1128993 # 1 # ID=1_1109;partial=00;start_type=ATG;rbs_motif=AGGA;rbs_spacer=5-10bp;gc_cont=0.468						
	PROJECT ID	ACCESSION ID	ORGANISMS	CLASS	PROTEIN FUNCTION	PROTEIN ID	%IDENTITY
Matched Family	28537	CP001084	Lactobacillus casei str. Zhang, complete genome.	Lactobacillales	Triosephosphate isomerase	ADK18158	100.0

Input Sequence	ATG-E1_Chromosome_3039 # 3083478 # 3084233 # -1 # ID=1_3039;partial=00;start_type=GTG;rbs_motif=AGGA;rbs_spacer=5-10bp;gc_cont=0.468						
	PROJECT ID	ACCESSION ID	ORGANISMS	CLASS	PROTEIN FUNCTION	PROTEIN ID	%IDENTITY
Matched Family	28537	CP001084	Lactobacillus casei str. Zhang, complete genome.	Lactobacillales	Protein tyrosine/serine phosphatase	ADK19984	100.0

Input Sequence	ATG-E1_Chromosome_976 # 990096 # 990848 # -1 # ID=1_976;partial=00;start_type=ATG;rbs_motif=AGGA;rbs_spacer=5-10bp;gc_cont=0.505						
	PROJECT ID	ACCESSION ID	ORGANISMS	CLASS	PROTEIN FUNCTION	PROTEIN ID	%IDENTITY
Matched Family	28537	CP001084	Lactobacillus casei str. Zhang, complete genome.	Lactobacillales	Biotin operon repressor	ADK18040	100.0

Input Sequence	ATG-E1_Chromosome_630 # 656591 # 656911 # 1 # ID=1_630;partial=00;start_type=ATG;rbs_motif=AGxAGG/AGGxGG;rbs_spacer=5-10bp;gc_cont=0.442						
	PROJECT ID	ACCESSION ID	ORGANISMS	CLASS	PROTEIN FUNCTION	PROTEIN ID	%IDENTITY
Matched Family	30359	FM177140	Lactobacillus casei BL23 complete genome, strain BL23.	Lactobacillales	Putative uncharacterized protein	CAQ65690	100.0

Input Sequence	ATG-E1_Chromosome_1722 # 1728457 # 1729200 # -1 # ID=1_1722;partial=00;start_type=ATG;rbs_motif=GGAG/GAGG;rbs_spacer=5-10bp;gc_cont=0.489						
	PROJECT ID	ACCESSION ID	ORGANISMS	CLASS	PROTEIN FUNCTION	PROTEIN ID	%IDENTITY
Matched Family	28537	CP001084	Lactobacillus casei str. Zhang, complete genome.	Lactobacillales	Predicted SAM-dependent methyltransferase	ADK18720	100.0

Input Sequence	ATG-E1_Chromosome_982 # 995645 # 996382 # 1 # ID=1_982;partial=00;start_type=ATG;rbs_motif=AGxAGG/AGGxGG;rbs_spacer=5-10bp;gc_cont=0.457						
	PROJECT ID	ACCESSION ID	ORGANISMS	CLASS	PROTEIN FUNCTION	PROTEIN ID	%IDENTITY
Matched Family	30359	FM177140	Lactobacillus casei BL23 complete genome, strain BL23.	Lactobacillales	Putative uncharacterized protein yibF	CAQ66005	100.0

Input Sequence	ATG-E1_Chromosome_2016 # 2027866 # 2028603 # -1 # ID=1_2016;partial=00;start_type=TTG;rbs_motif=AGxAGG/AGGxGG;rbs_spacer=5-10bp;gc_cont=0.408						
	PROJECT ID	ACCESSION ID	ORGANISMS	CLASS	PROTEIN FUNCTION	PROTEIN ID	%IDENTITY
Matched Family	28537	CP001084	Lactobacillus casei str. Zhang, complete genome.	Lactobacillales	Transcriptional regulator	ADK18993	100.0

Input Sequence	ATG-E1_Chromosome_2110 # 2125938 # 2126675 # 1 # ID=1_2110;partial=00;start_type=ATG;rbs_motif=GGAGG;rbs_spacer=5-10bp;gc_cont=0.550						
	PROJECT ID	ACCESSION ID	ORGANISMS	CLASS	PROTEIN FUNCTION	PROTEIN ID	%IDENTITY
Matched Family	28537	CP001084	Lactobacillus casei str. Zhang, complete genome.	Lactobacillales	Predicted permease	ADK19089	100.0

Input Sequence	ATG-E1_Chromosome_1427 # 1412395 # 1413126 # 1 # ID=1_1427;partial=00;start_type=GTG;rbs_motif=GGAG/GAGG;rbs_spacer=5-10bp;gc_cont=0.448

	PROJECT ID	ACCESSION ID	ORGANISMS	CLASS	PROTEIN FUNCTION	PROTEIN ID	%IDENTITY
**Matched Family**	28537	CP001084	Lactobacillus casei str. Zhang, complete genome.	Lactobacillales	Nicotinamide mononucleotide transporter	ADK18431	100.0

Input Sequence: ATG-E1_Chromosome_236 # 246974 # 247705 # -1 # ID=1_236;partial=00;start_type=ATG;rbs_motif=GGAGG;rbs_spacer=5-10bp;gc_cont=0.396

	PROJECT ID	ACCESSION ID	ORGANISMS	CLASS	PROTEIN FUNCTION	PROTEIN ID	%IDENTITY
**Matched Family**	28537	CP001084	Lactobacillus casei str. Zhang, complete genome.	Lactobacillales	putative taurine transport ATP-binding protein tauB	ADK17463	100.0

Input Sequence: ATG-E1_Chromosome_2882 # 2928449 # 2929177 # -1 # ID=1_2882;partial=00;start_type=ATG;rbs_motif=AGGA;rbs_spacer=5-10bp;gc_cont=0.458

	PROJECT ID	ACCESSION ID	ORGANISMS	CLASS	PROTEIN FUNCTION	PROTEIN ID	%IDENTITY
**Matched Family**	28537	CP001084	Lactobacillus casei str. Zhang, complete genome.	Lactobacillales	conserved hypothetical protein	ADK19833	100.0

Input Sequence: ATG-E1_Chromosome_645 # 667224 # 667952 # 1 # ID=1_645;partial=00;start_type=ATG;rbs_motif=GGAGG;rbs_spacer=5-10bp;gc_cont=0.492

	PROJECT ID	ACCESSION ID	ORGANISMS	CLASS	PROTEIN FUNCTION	PROTEIN ID	%IDENTITY
**Matched Family**	30359	FM177140	Lactobacillus casei BL23 complete genome, strain BL23.	Lactobacillales	ISSth1, transposase (Orf1), IS3 family	CAQ65705	100.0

Input Sequence: ATG-E1_Chromosome_1059 # 1073447 # 1074175 # 1 # ID=1_1059;partial=00;start_type=ATG;rbs_motif=GGAGG;rbs_spacer=5-10bp;gc_cont=0.464

	PROJECT ID	ACCESSION ID	ORGANISMS	CLASS	PROTEIN FUNCTION	PROTEIN ID	%IDENTITY
**Matched Family**	30359	FM177140	Lactobacillus casei BL23 complete genome, strain BL23.	Lactobacillales	3-oxoacyl-[acyl-carrier protein] reductase	CAQ66118	100.0

Input Sequence: ATG-E1_Chromosome_1428 # 1413202 # 1413930 # 1 # ID=1_1428;partial=00;start_type=GTG;rbs_motif=AGGAG;rbs_spacer=5-10bp;gc_cont=0.449

	PROJECT ID	ACCESSION ID	ORGANISMS	CLASS	PROTEIN FUNCTION	PROTEIN ID	%IDENTITY
**Matched Family**	28537	CP001084	Lactobacillus casei str. Zhang, complete genome.	Lactobacillales	Deoxynucleoside kinase	ADK18432	100.0

Input Sequence: ATG-E1_Chromosome_435 # 444519 # 445247 # -1 # ID=1_435;partial=00;start_type=ATG;rbs_motif=AGGAGG;rbs_spacer=5-10bp;gc_cont=0.432

	PROJECT ID	ACCESSION ID	ORGANISMS	CLASS	PROTEIN FUNCTION	PROTEIN ID	%IDENTITY
**Matched Family**	30359	FM177140	Lactobacillus casei BL23 complete genome, strain BL23.	Lactobacillales	Putative uncharacterized protein	CAQ65466	100.0

Input Sequence: ATG-E1_Chromosome_2881 # 2927335 # 2928060 # 1 # ID=1_2881;partial=00;start_type=TTG;rbs_motif=GGAG/GAGG;rbs_spacer=5-10bp;gc_cont=0.471

	PROJECT ID	ACCESSION ID	ORGANISMS	CLASS	PROTEIN FUNCTION	PROTEIN ID	%IDENTITY
**Matched Family**	28537	CP001084	Lactobacillus casei str. Zhang, complete genome.	Lactobacillales	Transcriptional regulator	ADK19832	100.0

Input Sequence: ATG-E1_Chromosome_1369 # 1358740 # 1359462 # 1 # ID=1_1369;partial=00;start_type=ATG;rbs_motif=AGGAG;rbs_spacer=5-10bp;gc_cont=0.528

	PROJECT ID	ACCESSION ID	ORGANISMS	CLASS	PROTEIN FUNCTION	PROTEIN ID	%IDENTITY
**Matched Family**	402	CP000423	Lactobacillus casei ATCC 334, complete genome.	Lactobacillales	Phosphoglycerate mutase family protein	ABJ69935	100.0

Input Sequence: ATG-E1_Chromosome_336 # 345975 # 346541 # 1 # ID=1_336;partial=00;start_type=ATG;rbs_motif=None;rbs_spacer=None;gc_cont=0.478

	PROJECT ID	ACCESSION ID	ORGANISMS	CLASS	PROTEIN FUNCTION	PROTEIN ID	%IDENTITY
**Matched Family**	30359	FM177140	Lactobacillus casei BL23 complete genome, strain BL23.	Lactobacillales	ABC transporter	CAQ65372	100.0

Input Sequence: ATG-E1_Chromosome_2073 # 2094244 # 2094954 # -1 # ID=1_2073;partial=00;start_type=GTG;rbs_motif=AGGAGG;rbs_spacer=5-10bp;gc_cont=0.461

	PROJECT ID	ACCESSION ID	ORGANISMS	CLASS	PROTEIN FUNCTION	PROTEIN ID	%IDENTITY
**Matched**	28537	CP001084	Lactobacillus casei str. Zhang,	Lactobacillales	Alpha-acetolactate decarboxylase	ADK19052	100.0

Family			complete genome.				

Input Sequence: ATG-E1_Chromosome_16 # 14390 # 15100 # -1 # ID=1_16;partial=00;start_type=ATG;rbs_motif=AGxAGG/AGGxGG;rbs_spacer=5-10bp;gc_cont=0.467

	PROJECT ID	ACCESSION ID	ORGANISMS	CLASS	PROTEIN FUNCTION	PROTEIN ID	%IDENTITY
Matched Family	30359	FM177140	Lactobacillus casei BL23 complete genome, strain BL23.	Lactobacillales	Putative uncharacterized protein	CAQ68094	100.0

Input Sequence: ATG-E1_Chromosome_3068 # 3117172 # 3117882 # -1 # ID=1_3068;partial=00;start_type=ATG;rbs_motif=GGAGG;rbs_spacer=5-10bp;gc_cont=0.471

	PROJECT ID	ACCESSION ID	ORGANISMS	CLASS	PROTEIN FUNCTION	PROTEIN ID	%IDENTITY
Matched Family	28537	CP001084	Lactobacillus casei str. Zhang, complete genome.	Lactobacillales	Transcriptional regulator	ADK20014	100.0

Input Sequence: ATG-E1_Chromosome_2119 # 2133264 # 2133971 # 1 # ID=1_2119;partial=00;start_type=ATG;rbs_motif=AGGAGG;rbs_spacer=5-10bp;gc_cont=0.438

	PROJECT ID	ACCESSION ID	ORGANISMS	CLASS	PROTEIN FUNCTION	PROTEIN ID	%IDENTITY
Matched Family	402	CP000423	Lactobacillus casei ATCC 334, complete genome.	Lactobacillales	ABC-type antimicrobial peptide transport system, ATPase component	ABJ70643	100.0

Input Sequence: ATG-E1_Chromosome_93 # 106366 # 107070 # -1 # ID=1_93;partial=00;start_type=ATG;rbs_motif=GGAGG;rbs_spacer=5-10bp;gc_cont=0.460

	PROJECT ID	ACCESSION ID	ORGANISMS	CLASS	PROTEIN FUNCTION	PROTEIN ID	%IDENTITY
Matched Family	28537	CP001084	Lactobacillus casei str. Zhang, complete genome.	Lactobacillales	Deoxyribose-phosphate aldolase	ADK17307	100.0

Input Sequence: ATG-E1_Chromosome_2767 # 2806603 # 2807307 # 1 # ID=1_2767;partial=00;start_type=GTG;rbs_motif=GGAGG;rbs_spacer=5-10bp;gc_cont=0.455

	PROJECT ID	ACCESSION ID	ORGANISMS	CLASS	PROTEIN FUNCTION	PROTEIN ID	%IDENTITY
Matched Family	28537	CP001084	Lactobacillus casei str. Zhang, complete genome.	Lactobacillales	conserved hypothetical protein	ADK19708	100.0

Input Sequence: ATG-E1_Chromosome_759 # 767594 # 768295 # 1 # ID=1_759;partial=00;start_type=ATG;rbs_motif=AGGAGG;rbs_spacer=5-10bp;gc_cont=0.484

	PROJECT ID	ACCESSION ID	ORGANISMS	CLASS	PROTEIN FUNCTION	PROTEIN ID	%IDENTITY
Matched Family	30359	FM177140	Lactobacillus casei BL23 complete genome, strain BL23.	Lactobacillales	Putative uncharacterized protein	CAQ65798	100.0

Input Sequence: ATG-E1_Chromosome_2330 # 2360330 # 2361031 # -1 # ID=1_2330;partial=00;start_type=ATG;rbs_motif=AGGAGG;rbs_spacer=5-10bp;gc_cont=0.423

	PROJECT ID	ACCESSION ID	ORGANISMS	CLASS	PROTEIN FUNCTION	PROTEIN ID	%IDENTITY
Matched Family	28537	CP001084	Lactobacillus casei str. Zhang, complete genome.	Lactobacillales	Sortase (surface protein transpeptidase)	ADK19309	100.0

Input Sequence: ATG-E1_Chromosome_343 # 354323 # 355009 # -1 # ID=1_343;partial=00;start_type=ATG;rbs_motif=None;rbs_spacer=None;gc_cont=0.477

	PROJECT ID	ACCESSION ID	ORGANISMS	CLASS	PROTEIN FUNCTION	PROTEIN ID	%IDENTITY
Matched Family	28537	CP001084	Lactobacillus casei str. Zhang, complete genome.	Lactobacillales	Cell wall-associated hydrolase	ADK17569	100.0

Input Sequence: ATG-E1_Chromosome_1424 # 1409381 # 1410064 # 1 # ID=1_1424;partial=00;start_type=GTG;rbs_motif=AGGAGG;rbs_spacer=5-10bp;gc_cont=0.484

	PROJECT ID	ACCESSION ID	ORGANISMS	CLASS	PROTEIN FUNCTION	PROTEIN ID	%IDENTITY
Matched Family	28537	CP001084	Lactobacillus casei str. Zhang, complete genome.	Lactobacillales	Phosphoglycerate mutase family protein	ADK18428	100.0

Input Sequence: ATG-E1_Chromosome_1899 # 1912159 # 1912842 # -1 # ID=1_1899;partial=00;start_type=ATG;rbs_motif=GGA/GAG/AGG;rbs_spacer=5-10bp;gc_cont=0.469

	PROJECT ID	ACCESSION ID	ORGANISMS	CLASS	PROTEIN FUNCTION	PROTEIN ID	%IDENTITY
Matched Family	28537	CP001084	Lactobacillus casei str. Zhang, complete genome.	Lactobacillales	Membrane-associated serine protease	ADK18879	100.0

Input Sequence	ATG-E1_Chromosome_301 # 309821 # 310492 # -1 # ID=1_301;partial=00;start_type=ATG;rbs_motif=GGAGG;rbs_spacer=5-10bp;gc_cont=0.440					
	PROJECT ID	ACCESSION ID	ORGANISMS	CLASS	PROTEIN FUNCTION	PROTEIN ID	%IDENTITY
Matched Family	402	CP000423	Lactobacillus casei ATCC 334, complete genome.	Lactobacillales	Predicted glutamine amidotransferase	ABJ69071	100.0

Input Sequence	ATG-E1_Chromosome_841 # 860399 # 861067 # 1 # ID=1_841;partial=00;start_type=GTG;rbs_motif=AGGA;rbs_spacer=5-10bp;gc_cont=0.471						
	PROJECT ID	ACCESSION ID	ORGANISMS	CLASS	PROTEIN FUNCTION	PROTEIN ID	%IDENTITY
Matched Family	28537	CP001084	Lactobacillus casei str. Zhang, complete genome.	Lactobacillales	Putative NADPH-quinone reductase (modulator of drug activity B)	ADK17913	100.0

Input Sequence	ATG-E1_Chromosome_923 # 934762 # 935430 # 1 # ID=1_923;partial=00;start_type=ATG;rbs_motif=AGxAGG/AGGxGG;rbs_spacer=5-10bp;gc_cont=0.453						
	PROJECT ID	ACCESSION ID	ORGANISMS	CLASS	PROTEIN FUNCTION	PROTEIN ID	%IDENTITY
Matched Family	28537	CP001084	Lactobacillus casei str. Zhang, complete genome.	Lactobacillales	Predicted phosphatase	ADK17982	100.0

Input Sequence	ATG-E1_Chromosome_1474 # 1461289 # 1461957 # 1 # ID=1_1474;partial=00;start_type=ATG;rbs_motif=AGxAGG/AGGxGG;rbs_spacer=5-10bp;gc_cont=0.490						
	PROJECT ID	ACCESSION ID	ORGANISMS	CLASS	PROTEIN FUNCTION	PROTEIN ID	%IDENTITY
Matched Family	28537	CP001084	Lactobacillus casei str. Zhang, complete genome.	Lactobacillales	DNA repair protein	ADK18478	100.0

Input Sequence	ATG-E1_Chromosome_1506 # 1492164 # 1492826 # -1 # ID=1_1506;partial=00;start_type=GTG;rbs_motif=GGAG/GAGG;rbs_spacer=5-10bp;gc_cont=0.465						
	PROJECT ID	ACCESSION ID	ORGANISMS	CLASS	PROTEIN FUNCTION	PROTEIN ID	%IDENTITY
Matched Family	402	CP000423	Lactobacillus casei ATCC 334, complete genome.	Lactobacillales	5-bromo-4-chloroindolyl phosphate hydrolysis protein	ABJ70068	100.0

Input Sequence	ATG-E1_Chromosome_157 # 165301 # 165960 # 1 # ID=1_157;partial=00;start_type=ATG;rbs_motif=AGGAGG;rbs_spacer=5-10bp;gc_cont=0.514						
	PROJECT ID	ACCESSION ID	ORGANISMS	CLASS	PROTEIN FUNCTION	PROTEIN ID	%IDENTITY
Matched Family	28537	CP001084	Lactobacillus casei str. Zhang, complete genome.	Lactobacillales	Probable transcriptional regulator	ADK17380	100.0

Input Sequence	ATG-E1_Chromosome_1479 # 1465006 # 1465248 # 1 # ID=1_1479;partial=00;start_type=ATG;rbs_motif=None;rbs_spacer=None;gc_cont=0.514						
	PROJECT ID	ACCESSION ID	ORGANISMS	CLASS	PROTEIN FUNCTION	PROTEIN ID	%IDENTITY
Matched Family	28537	CP001084	Lactobacillus casei str. Zhang, complete genome.	Lactobacillales	Septum formation inhibitor	ADK18482	100.0

Input Sequence	ATG-E1_Chromosome_1470 # 1455151 # 1455804 # 1 # ID=1_1470;partial=00;start_type=GTG;rbs_motif=GGAGG;rbs_spacer=5-10bp;gc_cont=0.492						
	PROJECT ID	ACCESSION ID	ORGANISMS	CLASS	PROTEIN FUNCTION	PROTEIN ID	%IDENTITY
Matched Family	30359	FM177140	Lactobacillus casei BL23 complete genome, strain BL23.	Lactobacillales	Redox-sensing transcriptional repressor rex	CAQ66561	100.0

Input Sequence	ATG-E1_Chromosome_152 # 161031 # 161684 # -1 # ID=1_152;partial=00;start_type=TTG;rbs_motif=GGAG/GAGG;rbs_spacer=5-10bp;gc_cont=0.390						
	PROJECT ID	ACCESSION ID	ORGANISMS	CLASS	PROTEIN FUNCTION	PROTEIN ID	%IDENTITY
Matched Family	402	CP000423	Lactobacillus casei ATCC 334, complete genome.	Lactobacillales	Predicted metal-dependent membrane protease	ABJ68931	100.0

Input Sequence	ATG-E1_Chromosome_1370 # 1359646 # 1360296 # 1 # ID=1_1370;partial=00;start_type=ATG;rbs_motif=AGGAGG;rbs_spacer=5-10bp;gc_cont=0.449						
	PROJECT ID	ACCESSION ID	ORGANISMS	CLASS	PROTEIN FUNCTION	PROTEIN ID	%IDENTITY
Matched Family	28537	CP001084	Lactobacillus casei str. Zhang, complete genome.	Lactobacillales	Transcriptional regulator	ADK18374	100.0

Input Sequence	ATG-E1_Chromosome_849 # 867362 # 868012 # 1 # ID=1_849;partial=00;start_type=ATG;rbs_motif=GGAG/GAGG;rbs_spacer=5-10bp;gc_cont=0.513

	PROJECT ID	ACCESSION ID	ORGANISMS	CLASS	PROTEIN FUNCTION	PROTEIN ID	%IDENTITY
**Matched Family**	30359	FM177140	Lactobacillus casei BL23 complete genome, strain BL23.	Lactobacillales	YwnB	CAQ65888	100.0

**Input Sequence**	ATG-E1_Chromosome_2846 # 2889815 # 2890459 # -1 # ID=1_2846;partial=00;start_type=ATG;rbs_motif=AGGA;rbs_spacer=5-10bp;gc_cont=0.490

	PROJECT ID	ACCESSION ID	ORGANISMS	CLASS	PROTEIN FUNCTION	PROTEIN ID	%IDENTITY
**Matched Family**	402	CP000423	Lactobacillus casei ATCC 334, complete genome.	Lactobacillales	Predicted metal-dependent membrane protease	ABJ71353	100.0

**Input Sequence**	ATG-E1_Chromosome_2604 # 2644256 # 2644894 # 1 # ID=1_2604;partial=00;start_type=ATG;rbs_motif=GGAG/GAGG;rbs_spacer=5-10bp;gc_cont=0.469

	PROJECT ID	ACCESSION ID	ORGANISMS	CLASS	PROTEIN FUNCTION	PROTEIN ID	%IDENTITY
**Matched Family**	28537	CP001084	Lactobacillus casei str. Zhang, complete genome.	Lactobacillales	integral membrane protein	ADK19592	100.0

**Input Sequence**	ATG-E1_Chromosome_121 # 129516 # 130154 # -1 # ID=1_121;partial=00;start_type=ATG;rbs_motif=AGxAGG/AGGxGG;rbs_spacer=11-12bp;gc_cont=0.473

	PROJECT ID	ACCESSION ID	ORGANISMS	CLASS	PROTEIN FUNCTION	PROTEIN ID	%IDENTITY
**Matched Family**	28537	CP001084	Lactobacillus casei str. Zhang, complete genome.	Lactobacillales	Transcriptional regulator	ADK17336	100.0

**Input Sequence**	ATG-E1_Chromosome_2257 # 2279765 # 2280403 # -1 # ID=1_2257;partial=00;start_type=ATG;rbs_motif=AGGAG;rbs_spacer=5-10bp;gc_cont=0.516

	PROJECT ID	ACCESSION ID	ORGANISMS	CLASS	PROTEIN FUNCTION	PROTEIN ID	%IDENTITY
**Matched Family**	28537	CP001084	Lactobacillus casei str. Zhang, complete genome.	Lactobacillales	Phosphoglycerate mutase family protein	ADK19231	100.0

**Input Sequence**	ATG-E1_Chromosome_1040 # 1054959 # 1055447 # -1 # ID=1_1040;partial=00;start_type=ATG;rbs_motif=GGA/GAG/AGG;rbs_spacer=5-10bp;gc_cont=0.462

	PROJECT ID	ACCESSION ID	ORGANISMS	CLASS	PROTEIN FUNCTION	PROTEIN ID	%IDENTITY
**Matched Family**	28537	CP001084	Lactobacillus casei str. Zhang, complete genome.	Lactobacillales	Dithiol-disulfide isomerase	ADK18089	100.0

**Input Sequence**	ATG-E1_Chromosome_1439 # 1422675 # 1423310 # 1 # ID=1_1439;partial=00;start_type=ATG;rbs_motif=AGxAG;rbs_spacer=5-10bp;gc_cont=0.478

	PROJECT ID	ACCESSION ID	ORGANISMS	CLASS	PROTEIN FUNCTION	PROTEIN ID	%IDENTITY
**Matched Family**	30359	FM177140	Lactobacillus casei BL23 complete genome, strain BL23.	Lactobacillales	NAD-dependent epimerase/dehydratase:3-beta hydroxysteroid dehydrogenase/isomerase:NmrA-like	CAQ66515	100.0

**Input Sequence**	ATG-E1_Chromosome_280 # 296098 # 296730 # 1 # ID=1_280;partial=00;start_type=ATG;rbs_motif=AGxAGG/AGGxGG;rbs_spacer=5-10bp;gc_cont=0.458

	PROJECT ID	ACCESSION ID	ORGANISMS	CLASS	PROTEIN FUNCTION	PROTEIN ID	%IDENTITY
**Matched Family**	28537	CP001084	Lactobacillus casei str. Zhang, complete genome.	Lactobacillales	conserved hypothetical protein	ADK17507	100.0

**Input Sequence**	ATG-E1_Chromosome_844 # 862079 # 862711 # 1 # ID=1_844;partial=00;start_type=ATG;rbs_motif=AGGAGG;rbs_spacer=5-10bp;gc_cont=0.483

	PROJECT ID	ACCESSION ID	ORGANISMS	CLASS	PROTEIN FUNCTION	PROTEIN ID	%IDENTITY
**Matched Family**	30359	FM177140	Lactobacillus casei BL23 complete genome, strain BL23.	Lactobacillales	Putative uncharacterized protein	CAQ65884	100.0

**Input Sequence**	ATG-E1_Chromosome_1611 # 1602584 # 1603213 # 1 # ID=1_1611;partial=00;start_type=GTG;rbs_motif=GGAG/GAGG;rbs_spacer=5-10bp;gc_cont=0.427

	PROJECT ID	ACCESSION ID	ORGANISMS	CLASS	PROTEIN FUNCTION	PROTEIN ID	%IDENTITY
**Matched Family**	28537	CP001084	Lactobacillus casei str. Zhang, complete genome.	Lactobacillales	conserved hypothetical protein	ADK18612	100.0

**Input Sequence**	ATG-E1_Chromosome_1754 # 1757298 # 1757924 # -1 # ID=1_1754;partial=00;start_type=ATG;rbs_motif=GGAGG;rbs_spacer=5-10bp;gc_cont=0.488

	PROJECT ID	ACCESSION ID	ORGANISMS	CLASS	PROTEIN FUNCTION	PROTEIN ID	%IDENTITY

	PROJECT ID	ACCESSION ID	ORGANISMS	CLASS	PROTEIN FUNCTION	PROTEIN ID	%IDENTITY
**Matched Family**	30359	FM177140	Lactobacillus casei BL23 complete genome, strain BL23.	Lactobacillales	Putative uncharacterized protein yjcF	CAQ66833	100.0

**Input Sequence**	ATG-E1_Chromosome_540 # 552801 # 553427 # 1 # ID=1_540;partial=00;start_type=ATG;rbs_motif=AGGA/GGAG/GAGG;rbs_spacer=11-12bp;gc_cont=0.415						
	PROJECT ID	ACCESSION ID	ORGANISMS	CLASS	PROTEIN FUNCTION	PROTEIN ID	%IDENTITY
**Matched Family**	30359	FM177140	Lactobacillus casei BL23 complete genome, strain BL23.	Lactobacillales	Putative flavodoxin	CAQ65589	100.0

**Input Sequence**	ATG-E1_Chromosome_833 # 851342 # 851968 # -1 # ID=1_833;partial=00;start_type=ATG;rbs_motif=GGAGG;rbs_spacer=5-10bp;gc_cont=0.504						
	PROJECT ID	ACCESSION ID	ORGANISMS	CLASS	PROTEIN FUNCTION	PROTEIN ID	%IDENTITY
**Matched Family**	28537	CP001084	Lactobacillus casei str. Zhang, complete genome.	Lactobacillales	Predicted phosphatase	ADK17902	100.0

**Input Sequence**	ATG-E1_Chromosome_1708 # 1708128 # 1708751 # -1 # ID=1_1708;partial=00;start_type=ATG;rbs_motif=GGAGG;rbs_spacer=5-10bp;gc_cont=0.458						
	PROJECT ID	ACCESSION ID	ORGANISMS	CLASS	PROTEIN FUNCTION	PROTEIN ID	%IDENTITY
**Matched Family**	28537	CP001084	Lactobacillus casei str. Zhang, complete genome.	Lactobacillales	Putative primosome component related protein	ADK18707	100.0

**Input Sequence**	ATG-E1_Chromosome_27 # 24766 # 25389 # -1 # ID=1_27;partial=00;start_type=ATG;rbs_motif=GGAG/GAGG;rbs_spacer=5-10bp;gc_cont=0.457						
	PROJECT ID	ACCESSION ID	ORGANISMS	CLASS	PROTEIN FUNCTION	PROTEIN ID	%IDENTITY
**Matched Family**	28537	CP001084	Lactobacillus casei str. Zhang, complete genome.	Lactobacillales	Uncharacterized membrane protein	ADK20051	100.0

**Input Sequence**	ATG-E1_Chromosome_1421 # 1406876 # 1407478 # 1 # ID=1_1421;partial=00;start_type=GTG;rbs_motif=GGA/GAG/AGG;rbs_spacer=5-10bp;gc_cont=0.511						
	PROJECT ID	ACCESSION ID	ORGANISMS	CLASS	PROTEIN FUNCTION	PROTEIN ID	%IDENTITY
**Matched Family**	402	CP000423	Lactobacillus casei ATCC 334, complete genome.	Lactobacillales	hypothetical protein	ABJ69984	100.0

**Input Sequence**	ATG-E1_Chromosome_1997 # 2008724 # 2009329 # -1 # ID=1_1997;partial=00;start_type=ATG;rbs_motif=AGGAGG;rbs_spacer=5-10bp;gc_cont=0.455						
	PROJECT ID	ACCESSION ID	ORGANISMS	CLASS	PROTEIN FUNCTION	PROTEIN ID	%IDENTITY
**Matched Family**	28537	CP001084	Lactobacillus casei str. Zhang, complete genome.	Lactobacillales	Predicted membrane protein	ADK18974	100.0

**Input Sequence**	ATG-E1_Chromosome_2032 # 2043197 # 2043802 # -1 # ID=1_2032;partial=00;start_type=ATG;rbs_motif=GGAGG;rbs_spacer=5-10bp;gc_cont=0.467						
	PROJECT ID	ACCESSION ID	ORGANISMS	CLASS	PROTEIN FUNCTION	PROTEIN ID	%IDENTITY
**Matched Family**	28537	CP001084	Lactobacillus casei str. Zhang, complete genome.	Lactobacillales	Predicted membrane protein	ADK19010	100.0

**Input Sequence**	ATG-E1_Chromosome_983 # 996384 # 996986 # 1 # ID=1_983;partial=00;start_type=ATG;rbs_motif=AGGA;rbs_spacer=5-10bp;gc_cont=0.448						
	PROJECT ID	ACCESSION ID	ORGANISMS	CLASS	PROTEIN FUNCTION	PROTEIN ID	%IDENTITY
**Matched Family**	402	CP000423	Lactobacillus casei ATCC 334, complete genome.	Lactobacillales	Predicted phosphoesterase or phosphohydrolase	ABJ69654	100.0

**Input Sequence**	ATG-E1_Chromosome_2212 # 2231817 # 2232419 # -1 # ID=1_2212;partial=00;start_type=ATG;rbs_motif=AGGAGG;rbs_spacer=5-10bp;gc_cont=0.464						
	PROJECT ID	ACCESSION ID	ORGANISMS	CLASS	PROTEIN FUNCTION	PROTEIN ID	%IDENTITY
**Matched Family**	28537	CP001084	Lactobacillus casei str. Zhang, complete genome.	Lactobacillales	Gmk, Guanylate kinase	ADK19177	100.0

**Input Sequence**	ATG-E1_Chromosome_146 # 155593 # 156195 # -1 # ID=1_146;partial=00;start_type=ATG;rbs_motif=GGAGG;rbs_spacer=5-10bp;gc_cont=0.466						
	PROJECT ID	ACCESSION ID	ORGANISMS	CLASS	PROTEIN FUNCTION	PROTEIN ID	%IDENTITY
**Matched Family**	30359	FM177140	Lactobacillus casei BL23 complete genome, strain BL23.	Lactobacillales	HTH-type transcriptional repressor Bm3R1	CAQ65193	100.0

**Input Sequence**	ATG-E1_Chromosome_1366 # 1355643 # 1356245 # -1 # ID=1_1366;partial=00;start_type=ATG;rbs_motif=AGGAGG;rbs_spacer=11-12bp;gc_cont=0.438						
	PROJECT ID	ACCESSION ID	ORGANISMS	CLASS	PROTEIN FUNCTION	PROTEIN ID	%IDENTITY
**Matched Family**	28537	CP001084	Lactobacillus casei str. Zhang, complete genome.	Lactobacillales	Predicted integral membrane protein	ADK18369	100.0

**Input Sequence**	ATG-E1_Chromosome_349 # 358568 # 359167 # -1 # ID=1_349;partial=00;start_type=GTG;rbs_motif=GGA/GAG/AGG;rbs_spacer=5-10bp;gc_cont=0.422						
	PROJECT ID	ACCESSION ID	ORGANISMS	CLASS	PROTEIN FUNCTION	PROTEIN ID	%IDENTITY
**Matched Family**	28537	CP001084	Lactobacillus casei str. Zhang, complete genome.	Lactobacillales	Signal peptidase I	ADK17574	100.0

**Input Sequence**	ATG-E1_Chromosome_1041 # 1055663 # 1056256 # -1 # ID=1_1041;partial=00;start_type=ATG;rbs_motif=GGAGG;rbs_spacer=5-10bp;gc_cont=0.446						
	PROJECT ID	ACCESSION ID	ORGANISMS	CLASS	PROTEIN FUNCTION	PROTEIN ID	%IDENTITY
**Matched Family**	28537	CP001084	Lactobacillus casei str. Zhang, complete genome.	Lactobacillales	conserved hypothetical protein	ADK18090	100.0

**Input Sequence**	ATG-E1_Chromosome_2322 # 2352335 # 2352922 # -1 # ID=1_2322;partial=00;start_type=ATG;rbs_motif=AGGAG;rbs_spacer=5-10bp;gc_cont=0.497						
	PROJECT ID	ACCESSION ID	ORGANISMS	CLASS	PROTEIN FUNCTION	PROTEIN ID	%IDENTITY
**Matched Family**	28537	CP001084	Lactobacillus casei str. Zhang, complete genome.	Lactobacillales	Transcriptional regulator	ADK19301	100.0

**Input Sequence**	ATG-E1_Chromosome_2510 # 2549853 # 2550440 # -1 # ID=1_2510;partial=00;start_type=ATG;rbs_motif=AGGAG;rbs_spacer=5-10bp;gc_cont=0.512						
	PROJECT ID	ACCESSION ID	ORGANISMS	CLASS	PROTEIN FUNCTION	PROTEIN ID	%IDENTITY
**Matched Family**	30359	FM177140	Lactobacillus casei BL23 complete genome, strain BL23.	Lactobacillales	Putative uncharacterized protein	CAQ67556	100.0

**Input Sequence**	ATG-E1_Chromosome_564 # 581420 # 582001 # -1 # ID=1_564;partial=00;start_type=ATG;rbs_motif=GGxGG;rbs_spacer=5-10bp;gc_cont=0.510						
	PROJECT ID	ACCESSION ID	ORGANISMS	CLASS	PROTEIN FUNCTION	PROTEIN ID	%IDENTITY
**Matched Family**	28537	CP001084	Lactobacillus casei str. Zhang, complete genome.	Lactobacillales	Dihydroxyacetone kinase	ADK17744	100.0

**Input Sequence**	ATG-E1_Chromosome_1592 # 1583517 # 1584095 # 1 # ID=1_1592;partial=00;start_type=ATG;rbs_motif=GGAGG;rbs_spacer=5-10bp;gc_cont=0.485						
	PROJECT ID	ACCESSION ID	ORGANISMS	CLASS	PROTEIN FUNCTION	PROTEIN ID	%IDENTITY
**Matched Family**	28537	CP001084	Lactobacillus casei str. Zhang, complete genome.	Lactobacillales	Predicted membrane protein	ADK18594	100.0

**Input Sequence**	ATG-E1_Chromosome_2078 # 2100131 # 2100706 # 1 # ID=1_2078;partial=00;start_type=ATG;rbs_motif=AGGAGG;rbs_spacer=5-10bp;gc_cont=0.443						
	PROJECT ID	ACCESSION ID	ORGANISMS	CLASS	PROTEIN FUNCTION	PROTEIN ID	%IDENTITY
**Matched Family**	28537	CP001084	Lactobacillus casei str. Zhang, complete genome.	Lactobacillales	conserved hypothetical protein	ADK19058	100.0

**Input Sequence**	ATG-E1_Chromosome_264 # 276694 # 277263 # -1 # ID=1_264;partial=00;start_type=ATG;rbs_motif=AGGAG;rbs_spacer=5-10bp;gc_cont=0.456						
	PROJECT ID	ACCESSION ID	ORGANISMS	CLASS	PROTEIN FUNCTION	PROTEIN ID	%IDENTITY
**Matched Family**	28537	CP001084	Lactobacillus casei str. Zhang, complete genome.	Lactobacillales	Transcriptional regulator	ADK17493	100.0

**Input Sequence**	ATG-E1_Chromosome_1278 # 1295847 # 1296416 # -1 # ID=1_1278;partial=00;start_type=ATG;rbs_motif=AGGAG;rbs_spacer=5-10bp;gc_cont=0.439						
	PROJECT ID	ACCESSION ID	ORGANISMS	CLASS	PROTEIN FUNCTION	PROTEIN ID	%IDENTITY
**Matched Family**	28537	CP001084	Lactobacillus casei str. Zhang, complete genome.	Lactobacillales	ABC family transporter	ADK18345	100.0

Input Sequence	ATG-E1_Chromosome_2270 # 2293381 # 2293947 # 1 # ID=1_2270;partial=00;start_type=ATG;rbs_motif=GGAGG;rbs_spacer=5-10bp;gc_cont=0.483					
	PROJECT ID	ACCESSION ID	ORGANISMS	CLASS	PROTEIN FUNCTION	PROTEIN ID	%IDENTITY
Matched Family	28537	CP001084	Lactobacillus casei str. Zhang, complete genome.	Lactobacillales	conserved hypothetical protein	ADK19244	100.0

Input Sequence	ATG-E1_Chromosome_905 # 917159 # 917719 # -1 # ID=1_905;partial=00;start_type=ATG;rbs_motif=GGAG/GAGG;rbs_spacer=5-10bp;gc_cont=0.528						
	PROJECT ID	ACCESSION ID	ORGANISMS	CLASS	PROTEIN FUNCTION	PROTEIN ID	%IDENTITY
Matched Family	30359	FM177140	Lactobacillus casei BL23 complete genome, strain BL23.	Lactobacillales	5-formyltetrahydrofolate cyclo-ligase	CAQ65925	100.0

Input Sequence	ATG-E1_Chromosome_2064 # 2080519 # 2081043 # -1 # ID=1_2064;partial=00;start_type=ATG;rbs_motif=AGxAG;rbs_spacer=5-10bp;gc_cont=0.451						
	PROJECT ID	ACCESSION ID	ORGANISMS	CLASS	PROTEIN FUNCTION	PROTEIN ID	%IDENTITY
Matched Family	28537	CP001084	Lactobacillus casei str. Zhang, complete genome.	Lactobacillales	conserved hypothetical protein	ADK19044	100.0

Input Sequence	ATG-E1_Chromosome_2413 # 2450955 # 2451515 # 1 # ID=1_2413;partial=00;start_type=ATG;rbs_motif=GGA/GAG/AGG;rbs_spacer=5-10bp;gc_cont=0.497						
	PROJECT ID	ACCESSION ID	ORGANISMS	CLASS	PROTEIN FUNCTION	PROTEIN ID	%IDENTITY
Matched Family	28537	CP001084	Lactobacillus casei str. Zhang, complete genome.	Lactobacillales	SAM-dependent methyltransferase	ADK19400	100.0

Input Sequence	ATG-E1_Chromosome_112 # 121314 # 121862 # 1 # ID=1_112;partial=00;start_type=ATG;rbs_motif=GGAGG;rbs_spacer=5-10bp;gc_cont=0.452						
	PROJECT ID	ACCESSION ID	ORGANISMS	CLASS	PROTEIN FUNCTION	PROTEIN ID	%IDENTITY
Matched Family	30359	FM177140	Lactobacillus casei BL23 complete genome, strain BL23.	Lactobacillales	Regulatory protein, TetR	CAQ65158	100.0

Input Sequence	ATG-E1_Chromosome_1578 # 1568329 # 1568880 # 1 # ID=1_1578;partial=00;start_type=ATG;rbs_motif=AGxAGG/AGGxGG;rbs_spacer=5-10bp;gc_cont=0.422						
	PROJECT ID	ACCESSION ID	ORGANISMS	CLASS	PROTEIN FUNCTION	PROTEIN ID	%IDENTITY
Matched Family	28537	CP001084	Lactobacillus casei str. Zhang, complete genome.	Lactobacillales	B	ADK18580	100.0

Input Sequence	ATG-E1_Chromosome_2075 # 2097001 # 2097552 # -1 # ID=1_2075;partial=00;start_type=ATG;rbs_motif=GGAGG;rbs_spacer=5-10bp;gc_cont=0.422						
	PROJECT ID	ACCESSION ID	ORGANISMS	CLASS	PROTEIN FUNCTION	PROTEIN ID	%IDENTITY
Matched Family	28537	CP001084	Lactobacillus casei str. Zhang, complete genome.	Lactobacillales	conserved hypothetical protein	ADK19054	100.0

Input Sequence	ATG-E1_Chromosome_26 # 24110 # 24658 # 1 # ID=1_26;partial=00;start_type=ATG;rbs_motif=None;rbs_spacer=None;gc_cont=0.483						
	PROJECT ID	ACCESSION ID	ORGANISMS	CLASS	PROTEIN FUNCTION	PROTEIN ID	%IDENTITY
Matched Family	30359	FM177140	Lactobacillus casei BL23 complete genome, strain BL23.	Lactobacillales	YdaF (GCN5-related N-acetyltransferase)	CAQ68105	100.0

Input Sequence	ATG-E1_Chromosome_766 # 775644 # 776192 # -1 # ID=1_766;partial=00;start_type=ATG;rbs_motif=GGAGG;rbs_spacer=5-10bp;gc_cont=0.472						
	PROJECT ID	ACCESSION ID	ORGANISMS	CLASS	PROTEIN FUNCTION	PROTEIN ID	%IDENTITY
Matched Family	30359	FM177140	Lactobacillus casei BL23 complete genome, strain BL23.	Lactobacillales	Hypothetical membrane spanning protein	CAQ65805	100.0

Input Sequence	ATG-E1_Chromosome_1386 # 1376882 # 1377427 # 1 # ID=1_1386;partial=00;start_type=ATG;rbs_motif=GGA/GAG/AGG;rbs_spacer=5-10bp;gc_cont=0.498						
	PROJECT ID	ACCESSION ID	ORGANISMS	CLASS	PROTEIN FUNCTION	PROTEIN ID	%IDENTITY
Matched Family	30359	FM177140	Lactobacillus casei BL23 complete genome, strain BL23.	Lactobacillales	H(+)-transporting two-sector ATPase (ATP synthase), delta subunit	CAQ66466	100.0

Input Sequence	ATG-E1_Chromosome_628 # 655558 # 656103 # -1 # ID=1_628;partial=00;start_type=ATG;rbs_motif=GGAG/GAGG;rbs_spacer=5-10bp;gc_cont=0.383

	PROJECT ID	ACCESSION ID	ORGANISMS	CLASS	PROTEIN FUNCTION	PROTEIN ID	%IDENTITY
**Matched Family**	30359	FM177140	Lactobacillus casei BL23 complete genome, strain BL23.	Lactobacillales	Putative uncharacterized protein	CAQ65688	100.0

Input Sequence: ATG-E1_Chromosome_2528 # 2568636 # 2569178 # -1 # ID=1_2528;partial=00;start_type=ATG;rbs_motif=GGAGG;rbs_spacer=5-10bp;gc_cont=0.460

	PROJECT ID	ACCESSION ID	ORGANISMS	CLASS	PROTEIN FUNCTION	PROTEIN ID	%IDENTITY
**Matched Family**	28537	CP001084	Lactobacillus casei str. Zhang, complete genome.	Lactobacillales	dUTPase	ADK19508	100.0

Input Sequence: ATG-E1_Chromosome_293 # 304686 # 305225 # 1 # ID=1_293;partial=00;start_type=ATG;rbs_motif=AGGAGG;rbs_spacer=5-10bp;gc_cont=0.500

	PROJECT ID	ACCESSION ID	ORGANISMS	CLASS	PROTEIN FUNCTION	PROTEIN ID	%IDENTITY
**Matched Family**	28537	CP001084	Lactobacillus casei str. Zhang, complete genome.	Lactobacillales	conserved hypothetical protein	ADK17520	100.0

Input Sequence: ATG-E1_Chromosome_954 # 966720 # 967253 # 1 # ID=1_954;partial=00;start_type=TTG;rbs_motif=GGxGG;rbs_spacer=5-10bp;gc_cont=0.457

	PROJECT ID	ACCESSION ID	ORGANISMS	CLASS	PROTEIN FUNCTION	PROTEIN ID	%IDENTITY
**Matched Family**	28537	CP001084	Lactobacillus casei str. Zhang, complete genome.	Lactobacillales	Predicted membrane ancor connecting MutS2 with cell-division Z-ring	ADK18018	100.0

Input Sequence: ATG-E1_Chromosome_1477 # 1464049 # 1464579 # 1 # ID=1_1477;partial=00;start_type=ATG;rbs_motif=GGA/GAG/AGG;rbs_spacer=5-10bp;gc_cont=0.427

	PROJECT ID	ACCESSION ID	ORGANISMS	CLASS	PROTEIN FUNCTION	PROTEIN ID	%IDENTITY
**Matched Family**	402	CP000423	Lactobacillus casei ATCC 334, complete genome.	Lactobacillales	rod shape-determining protein MreD	ABJ70041	100.0

Input Sequence: ATG-E1_Chromosome_2076 # 2097672 # 2098196 # -1 # ID=1_2076;partial=00;start_type=ATG;rbs_motif=AGGAG;rbs_spacer=5-10bp;gc_cont=0.499

	PROJECT ID	ACCESSION ID	ORGANISMS	CLASS	PROTEIN FUNCTION	PROTEIN ID	%IDENTITY
**Matched Family**	30359	FM177140	Lactobacillus casei BL23 complete genome, strain BL23.	Lactobacillales	Ribosomal-protein-alanine acetyltransferase	CAQ67134	100.0

Input Sequence: ATG-E1_Chromosome_843 # 861321 # 861845 # 1 # ID=1_843;partial=00;start_type=ATG;rbs_motif=AGGAG;rbs_spacer=5-10bp;gc_cont=0.472

	PROJECT ID	ACCESSION ID	ORGANISMS	CLASS	PROTEIN FUNCTION	PROTEIN ID	%IDENTITY
**Matched Family**	30359	FM177140	Lactobacillus casei BL23 complete genome, strain BL23.	Lactobacillales	Putative uncharacterized protein	CAQ65883	100.0

Input Sequence: ATG-E1_Chromosome_433 # 443356 # 443880 # -1 # ID=1_433;partial=00;start_type=ATG;rbs_motif=AGxAGG/AGGxGG;rbs_spacer=11-12bp;gc_cont=0.392

	PROJECT ID	ACCESSION ID	ORGANISMS	CLASS	PROTEIN FUNCTION	PROTEIN ID	%IDENTITY
**Matched Family**	30359	FM177140	Lactobacillus casei BL23 complete genome, strain BL23.	Lactobacillales	Putative uncharacterized protein	CAQ65462	100.0

Input Sequence: ATG-E1_Chromosome_2009 # 2021477 # 2021995 # -1 # ID=1_2009;partial=00;start_type=ATG;rbs_motif=GGAGG;rbs_spacer=5-10bp;gc_cont=0.445

	PROJECT ID	ACCESSION ID	ORGANISMS	CLASS	PROTEIN FUNCTION	PROTEIN ID	%IDENTITY
**Matched Family**	28537	CP001084	Lactobacillus casei str. Zhang, complete genome.	Lactobacillales	conserved hypothetical protein	ADK18985	100.0

Input Sequence: ATG-E1_Chromosome_633 # 658175 # 658693 # 1 # ID=1_633;partial=00;start_type=ATG;rbs_motif=GGAG/GAGG;rbs_spacer=5-10bp;gc_cont=0.451

	PROJECT ID	ACCESSION ID	ORGANISMS	CLASS	PROTEIN FUNCTION	PROTEIN ID	%IDENTITY
**Matched Family**	30359	FM177140	Lactobacillus casei BL23 complete genome, strain BL23.	Lactobacillales	DNA polymerase III, alpha subunit	CAQ65692	100.0

Input Sequence: ATG-E1_Chromosome_897 # 910600 # 911079 # -1 # ID=1_897;partial=00;start_type=ATG;rbs_motif=AGGAG;rbs_spacer=5-10bp;gc_cont=0.463

	PROJECT ID	ACCESSION ID	ORGANISMS	CLASS	PROTEIN FUNCTION	PROTEIN ID	%IDENTITY
**Matched**	30359	FM177140	Lactobacillus casei BL23	Lactobacillales	Putative uncharacterized protein	CAQ65915	100.0

Family			complete genome, strain BL23.				

Input Sequence	ATG-E1_Chromosome_2905 # 2956878 # 2957390 # -1 # ID=1_2905;partial=00;start_type=ATG;rbs_motif=GGA/GAG/AGG;rbs_spacer=11-12bp;gc_cont=0.394						
	PROJECT ID	ACCESSION ID	ORGANISMS	CLASS	PROTEIN FUNCTION	PROTEIN ID	%IDENTITY
Matched Family	30359	FM177140	Lactobacillus casei BL23 complete genome, strain BL23.	Lactobacillales	Putative uncharacterized protein	CAQ67932	100.0

Input Sequence	ATG-E1_Chromosome_2946 # 2995300 # 2995809 # -1 # ID=1_2946;partial=00;start_type=ATG;rbs_motif=GGAGG;rbs_spacer=5-10bp;gc_cont=0.422						
	PROJECT ID	ACCESSION ID	ORGANISMS	CLASS	PROTEIN FUNCTION	PROTEIN ID	%IDENTITY
Matched Family	28537	CP001084	Lactobacillus casei str. Zhang, complete genome.	Lactobacillales	Glucitol operon activator	ADK19915	100.0

Input Sequence	ATG-E1_Chromosome_285 # 300113 # 300619 # -1 # ID=1_285;partial=00;start_type=ATG;rbs_motif=GGxGG;rbs_spacer=3-4bp;gc_cont=0.460						
	PROJECT ID	ACCESSION ID	ORGANISMS	CLASS	PROTEIN FUNCTION	PROTEIN ID	%IDENTITY
Matched Family	30359	FM177140	Lactobacillus casei BL23 complete genome, strain BL23.	Lactobacillales	Membrane protein	CAQ65313	100.0

Input Sequence	ATG-E1_Chromosome_1134 # 1155873 # 1156379 # -1 # ID=1_1134;partial=00;start_type=ATG;rbs_motif=GGAGG;rbs_spacer=5-10bp;gc_cont=0.460						
	PROJECT ID	ACCESSION ID	ORGANISMS	CLASS	PROTEIN FUNCTION	PROTEIN ID	%IDENTITY
Matched Family	28537	CP001084	Lactobacillus casei str. Zhang, complete genome.	Lactobacillales	conserved hypothetical protein	ADK18185	100.0

Input Sequence	ATG-E1_Chromosome_1976 # 1986176 # 1986682 # 1 # ID=1_1976;partial=00;start_type=ATG;rbs_motif=GGAGG;rbs_spacer=5-10bp;gc_cont=0.462						
	PROJECT ID	ACCESSION ID	ORGANISMS	CLASS	PROTEIN FUNCTION	PROTEIN ID	%IDENTITY
Matched Family	28537	CP001084	Lactobacillus casei str. Zhang, complete genome.	Lactobacillales	Monooxygenase	ADK18955	100.0

Input Sequence	ATG-E1_Chromosome_1209 # 1220291 # 1220794 # 1 # ID=1_1209;partial=00;start_type=ATG;rbs_motif=GGAG/GAGG;rbs_spacer=5-10bp;gc_cont=0.474						
	PROJECT ID	ACCESSION ID	ORGANISMS	CLASS	PROTEIN FUNCTION	PROTEIN ID	%IDENTITY
Matched Family	28537	CP001084	Lactobacillus casei str. Zhang, complete genome.	Lactobacillales	conserved hypothetical protein	ADK18268	100.0

Input Sequence	ATG-E1_Chromosome_491 # 503914 # 504417 # 1 # ID=1_491;partial=00;start_type=ATG;rbs_motif=AGGAGG;rbs_spacer=5-10bp;gc_cont=0.425						
	PROJECT ID	ACCESSION ID	ORGANISMS	CLASS	PROTEIN FUNCTION	PROTEIN ID	%IDENTITY
Matched Family	30359	FM177140	Lactobacillus casei BL23 complete genome, strain BL23.	Lactobacillales	Putative phosphotransferase system enzyme IIB	CAQ65539	100.0

Input Sequence	ATG-E1_Chromosome_962 # 975236 # 975730 # -1 # ID=1_962;partial=00;start_type=ATG;rbs_motif=AGGAGG;rbs_spacer=5-10bp;gc_cont=0.499						
	PROJECT ID	ACCESSION ID	ORGANISMS	CLASS	PROTEIN FUNCTION	PROTEIN ID	%IDENTITY
Matched Family	28537	CP001084	Lactobacillus casei str. Zhang, complete genome.	Lactobacillales	Predicted hydrocarbon binding protein, V4R domain	ADK18026	100.0

Input Sequence	ATG-E1_Chromosome_1760 # 1763242 # 1763736 # -1 # ID=1_1760;partial=00;start_type=ATG;rbs_motif=GGAG/GAGG;rbs_spacer=5-10bp;gc_cont=0.426						
	PROJECT ID	ACCESSION ID	ORGANISMS	CLASS	PROTEIN FUNCTION	PROTEIN ID	%IDENTITY
Matched Family	402	CP000423	Lactobacillus casei ATCC 334, complete genome.	Lactobacillales	hypothetical protein	ABJ70318	100.0

Input Sequence	ATG-E1_Chromosome_912 # 924122 # 924613 # 1 # ID=1_912;partial=00;start_type=ATG;rbs_motif=AGGAGG;rbs_spacer=5-10bp;gc_cont=0.455						
	PROJECT ID	ACCESSION ID	ORGANISMS	CLASS	PROTEIN FUNCTION	PROTEIN ID	%IDENTITY
Matched Family	402	CP000423	Lactobacillus casei ATCC 334, complete genome.	Lactobacillales	hypothetical protein	ABJ69580	100.0

Input Sequence	ATG-E1_Chromosome_2060 # 2077123 # 2077614 # -1 # ID=1_2060;partial=00;start_type=ATG;rbs_motif=GGA/GAG/AGG;rbs_spacer=5-10bp;gc_cont=0.530						
	PROJECT ID	ACCESSION ID	ORGANISMS	CLASS	PROTEIN FUNCTION	PROTEIN ID	%IDENTITY
Matched Family	28537	CP001084	Lactobacillus casei str. Zhang, complete genome.	Lactobacillales	Fe2+ transport system protein A	ADK19040	100.0

Input Sequence	ATG-E1_Chromosome_1711 # 1711365 # 1711853 # -1 # ID=1_1711;partial=00;start_type=ATG;rbs_motif=AGGAGG;rbs_spacer=5-10bp;gc_cont=0.419						
	PROJECT ID	ACCESSION ID	ORGANISMS	CLASS	PROTEIN FUNCTION	PROTEIN ID	%IDENTITY
Matched Family	30359	FM177140	Lactobacillus casei BL23 complete genome, strain BL23.	Lactobacillales	Putative uncharacterized protein	CAQ66790	100.0

Input Sequence	ATG-E1_Chromosome_76 # 87278 # 87766 # -1 # ID=1_76;partial=00;start_type=ATG;rbs_motif=GGAGG;rbs_spacer=5-10bp;gc_cont=0.497						
	PROJECT ID	ACCESSION ID	ORGANISMS	CLASS	PROTEIN FUNCTION	PROTEIN ID	%IDENTITY
Matched Family	28537	CP001084	Lactobacillus casei str. Zhang, complete genome.	Lactobacillales	conserved hypothetical protein	ADK20091	100.0

Input Sequence	ATG-E1_Chromosome_2818 # 2859231 # 2859710 # -1 # ID=1_2818;partial=00;start_type=ATG;rbs_motif=AGGAG;rbs_spacer=5-10bp;gc_cont=0.481						
	PROJECT ID	ACCESSION ID	ORGANISMS	CLASS	PROTEIN FUNCTION	PROTEIN ID	%IDENTITY
Matched Family	30359	FM177140	Lactobacillus casei BL23 complete genome, strain BL23.	Lactobacillales	NUDIX hydrolase	CAQ67823	100.0

Input Sequence	ATG-E1_Chromosome_837 # 857264 # 857740 # 1 # ID=1_837;partial=00;start_type=ATG;rbs_motif=AGGA/GGAG/GAGG;rbs_spacer=11-12bp;gc_cont=0.457						
	PROJECT ID	ACCESSION ID	ORGANISMS	CLASS	PROTEIN FUNCTION	PROTEIN ID	%IDENTITY
Matched Family	28537	CP001084	Lactobacillus casei str. Zhang, complete genome.	Lactobacillales	Molecular chaperone (small heat shock protein)	ADK17908	100.0

Input Sequence	ATG-E1_Chromosome_19 # 16174 # 16647 # 1 # ID=1_19;partial=00;start_type=ATG;rbs_motif=GGAGG;rbs_spacer=5-10bp;gc_cont=0.504						
	PROJECT ID	ACCESSION ID	ORGANISMS	CLASS	PROTEIN FUNCTION	PROTEIN ID	%IDENTITY
Matched Family	28537	CP001084	Lactobacillus casei str. Zhang, complete genome.	Lactobacillales	conserved hypothetical protein	ADK20042	100.0

Input Sequence	ATG-E1_Chromosome_2862 # 2906310 # 2906783 # -1 # ID=1_2862;partial=00;start_type=ATG;rbs_motif=GGxGG;rbs_spacer=5-10bp;gc_cont=0.462						
	PROJECT ID	ACCESSION ID	ORGANISMS	CLASS	PROTEIN FUNCTION	PROTEIN ID	%IDENTITY
Matched Family	28537	CP001084	Lactobacillus casei str. Zhang, complete genome.	Lactobacillales	conserved hypothetical protein	ADK19810	100.0

Input Sequence	ATG-E1_Chromosome_1514 # 1497252 # 1497722 # 1 # ID=1_1514;partial=00;start_type=GTG;rbs_motif=None;rbs_spacer=None;gc_cont=0.431						
	PROJECT ID	ACCESSION ID	ORGANISMS	CLASS	PROTEIN FUNCTION	PROTEIN ID	%IDENTITY
Matched Family	317	CP000411	Oenococcus oeni PSU-1, complete genome.	Lactobacillales	Transposase	ABJ56310	100.0

Input Sequence	ATG-E1_Chromosome_882 # 899344 # 899814 # 1 # ID=1_882;partial=00;start_type=ATG;rbs_motif=AGGA;rbs_spacer=5-10bp;gc_cont=0.463						
	PROJECT ID	ACCESSION ID	ORGANISMS	CLASS	PROTEIN FUNCTION	PROTEIN ID	%IDENTITY
Matched Family	32195	FM179322	Lactobacillus rhamnosus GG whole genome sequence, strain GG (ATCC 53103).	Lactobacillales	Phage-related terminase-small subunit	CAR88792	100.0

Input Sequence	ATG-E1_Chromosome_315 # 322961 # 323428 # 1 # ID=1_315;partial=00;start_type=ATG;rbs_motif=AGxAGG/AGGxGG;rbs_spacer=5-10bp;gc_cont=0.519						
	PROJECT ID	ACCESSION ID	ORGANISMS	CLASS	PROTEIN FUNCTION	PROTEIN ID	%IDENTITY
Matched Family	28537	CP001084	Lactobacillus casei str. Zhang, complete genome.	Lactobacillales	Transcription elongation factor	ADK17542	100.0

Input	ATG-E1_Chromosome_287 # 301249 # 301716 # 1 # ID=1_287;partial=00;start_type=ATG;rbs_motif=GGAG/GAGG;rbs_spacer=5-

Sequence 10bp;gc_cont=0.468

	PROJECT ID	ACCESSION ID	ORGANISMS	CLASS	PROTEIN FUNCTION	PROTEIN ID	%IDENTITY
**Matched Family**	30359	FM177140	Lactobacillus casei BL23 complete genome, strain BL23.	Lactobacillales	Flavodoxin	CAQ65315	100.0

Input Sequence ATG-E1_Chromosome_1248 # 1261780 # 1262241 # -1 # ID=1_1248;partial=00;start_type=ATG;rbs_motif=AGGAGG;rbs_spacer=11-12bp;gc_cont=0.476

	PROJECT ID	ACCESSION ID	ORGANISMS	CLASS	PROTEIN FUNCTION	PROTEIN ID	%IDENTITY
**Matched Family**	30359	FM177140	Lactobacillus casei BL23 complete genome, strain BL23.	Lactobacillales	Putative flavodoxin	CAQ66334	100.0

Input Sequence ATG-E1_Chromosome_2338 # 2369503 # 2369964 # -1 # ID=1_2338;partial=00;start_type=TTG;rbs_motif=AGxAGG/AGGxGG;rbs_spacer=5-10bp;gc_cont=0.494

	PROJECT ID	ACCESSION ID	ORGANISMS	CLASS	PROTEIN FUNCTION	PROTEIN ID	%IDENTITY
**Matched Family**	28537	CP001084	Lactobacillus casei str. Zhang, complete genome.	Lactobacillales	Universal stress protein UspA related nucleotide-binding protein	ADK19317	100.0

Input Sequence ATG-E1_Chromosome_359 # 369492 # 369950 # 1 # ID=1_359;partial=00;start_type=TTG;rbs_motif=AGGAGG;rbs_spacer=5-10bp;gc_cont=0.488

	PROJECT ID	ACCESSION ID	ORGANISMS	CLASS	PROTEIN FUNCTION	PROTEIN ID	%IDENTITY
**Matched Family**	28537	CP001084	Lactobacillus casei str. Zhang, complete genome.	Lactobacillales	conserved hypothetical protein	ADK17580	100.0

Input Sequence ATG-E1_Chromosome_924 # 935518 # 935976 # -1 # ID=1_924;partial=00;start_type=ATG;rbs_motif=GGAGG;rbs_spacer=5-10bp;gc_cont=0.460

	PROJECT ID	ACCESSION ID	ORGANISMS	CLASS	PROTEIN FUNCTION	PROTEIN ID	%IDENTITY
**Matched Family**	28537	CP001084	Lactobacillus casei str. Zhang, complete genome.	Lactobacillales	Transcriptional regulator	ADK17983	100.0

Input Sequence ATG-E1_Chromosome_1737 # 1742073 # 1742531 # -1 # ID=1_1737;partial=00;start_type=ATG;rbs_motif=AGGAG;rbs_spacer=5-10bp;gc_cont=0.481

	PROJECT ID	ACCESSION ID	ORGANISMS	CLASS	PROTEIN FUNCTION	PROTEIN ID	%IDENTITY
**Matched Family**	28537	CP001084	Lactobacillus casei str. Zhang, complete genome.	Lactobacillales	Predicted metal-dependent hydrolase	ADK18736	100.0

Input Sequence ATG-E1_Chromosome_1604 # 1595514 # 1595969 # 1 # ID=1_1604;partial=00;start_type=ATG;rbs_motif=AGxAGG/AGGxGG;rbs_spacer=5-10bp;gc_cont=0.445

	PROJECT ID	ACCESSION ID	ORGANISMS	CLASS	PROTEIN FUNCTION	PROTEIN ID	%IDENTITY
**Matched Family**	28537	CP001084	Lactobacillus casei str. Zhang, complete genome.	Lactobacillales	conserved hypothetical protein	ADK18605	100.0

Input Sequence ATG-E1_Chromosome_1873 # 1890290 # 1890742 # -1 # ID=1_1873;partial=00;start_type=ATG;rbs_motif=GGAG/GAGG;rbs_spacer=5-10bp;gc_cont=0.479

	PROJECT ID	ACCESSION ID	ORGANISMS	CLASS	PROTEIN FUNCTION	PROTEIN ID	%IDENTITY
**Matched Family**	402	CP000423	Lactobacillus casei ATCC 334, complete genome.	Lactobacillales	transcriptional regulator, ArgR family	ABJ70408	100.0

Input Sequence ATG-E1_Chromosome_2311 # 2342070 # 2342519 # -1 # ID=1_2311;partial=00;start_type=ATG;rbs_motif=GGAGG;rbs_spacer=5-10bp;gc_cont=0.447

	PROJECT ID	ACCESSION ID	ORGANISMS	CLASS	PROTEIN FUNCTION	PROTEIN ID	%IDENTITY
**Matched Family**	28537	CP001084	Lactobacillus casei str. Zhang, complete genome.	Lactobacillales	Transcriptional regulator	ADK19289	100.0

Input Sequence ATG-E1_Chromosome_1526 # 1511074 # 1511523 # 1 # ID=1_1526;partial=00;start_type=GTG;rbs_motif=GGAG/GAGG;rbs_spacer=5-10bp;gc_cont=0.453

	PROJECT ID	ACCESSION ID	ORGANISMS	CLASS	PROTEIN FUNCTION	PROTEIN ID	%IDENTITY
**Matched Family**	28537	CP001084	Lactobacillus casei str. Zhang, complete genome.	Lactobacillales	conserved hypothetical protein	ADK18529	100.0

Input Sequence ATG-E1_Chromosome_2210 # 2231079 # 2231525 # -1 # ID=1_2210;partial=00;start_type=ATG;rbs_motif=AGGAG;rbs_spacer=5-10bp;gc_cont=0.427

PROJECT ID	ACCESSION ID	ORGANISMS	CLASS	PROTEIN FUNCTION	PROTEIN ID	%IDENTITY

	PROJECT ID	ACCESSION ID	ORGANISMS	CLASS	PROTEIN FUNCTION	PROTEIN ID	%IDENTITY
**Matched Family**	30359	FM177140	Lactobacillus casei BL23 complete genome, strain BL23.	Lactobacillales	Ferric uptake regulation protein	CAQ67259	100.0

Input Sequence ATG-E1_Chromosome_490 # 503468 # 503914 # 1 # ID=1_490;partial=00;start_type=ATG;rbs_motif=GGAGG;rbs_spacer=5-10bp;gc_cont=0.425

	PROJECT ID	ACCESSION ID	ORGANISMS	CLASS	PROTEIN FUNCTION	PROTEIN ID	%IDENTITY
**Matched Family**	30359	FM177140	Lactobacillus casei BL23 complete genome, strain BL23.	Lactobacillales	Mannose-specific PTS system, component IIA	CAQ65538	100.0

Input Sequence ATG-E1_Chromosome_2481 # 2525440 # 2525883 # -1 # ID=1_2481;partial=00;start_type=ATG;rbs_motif=GGAGG;rbs_spacer=5-10bp;gc_cont=0.473

	PROJECT ID	ACCESSION ID	ORGANISMS	CLASS	PROTEIN FUNCTION	PROTEIN ID	%IDENTITY
**Matched Family**	28537	CP001084	Lactobacillus casei str. Zhang, complete genome.	Lactobacillales	Nucleoside 2-deoxyribosyltransferase	ADK19468	100.0

Input Sequence ATG-E1_Chromosome_1983 # 1994859 # 1995299 # -1 # ID=1_1983;partial=00;start_type=ATG;rbs_motif=GGAGG;rbs_spacer=5-10bp;gc_cont=0.442

	PROJECT ID	ACCESSION ID	ORGANISMS	CLASS	PROTEIN FUNCTION	PROTEIN ID	%IDENTITY
**Matched Family**	28537	CP001084	Lactobacillus casei str. Zhang, complete genome.	Lactobacillales	Predicted transcriptional regulator	ADK18962	100.0

Input Sequence ATG-E1_Chromosome_2209 # 2230347 # 2230787 # -1 # ID=1_2209;partial=00;start_type=ATG;rbs_motif=AGGAG;rbs_spacer=5-10bp;gc_cont=0.481

	PROJECT ID	ACCESSION ID	ORGANISMS	CLASS	PROTEIN FUNCTION	PROTEIN ID	%IDENTITY
**Matched Family**	30359	FM177140	Lactobacillus casei BL23 complete genome, strain BL23.	Lactobacillales	Putative uncharacterized protein	CAQ67258	100.0

Input Sequence ATG-E1_Chromosome_782 # 798450 # 798890 # -1 # ID=1_782;partial=00;start_type=ATG;rbs_motif=AGGA;rbs_spacer=5-10bp;gc_cont=0.483

	PROJECT ID	ACCESSION ID	ORGANISMS	CLASS	PROTEIN FUNCTION	PROTEIN ID	%IDENTITY
**Matched Family**	28537	CP001084	Lactobacillus casei str. Zhang, complete genome.	Lactobacillales	Predicted redox protein, regulator of disulfide bond formation	ADK17849	100.0

Input Sequence ATG-E1_Chromosome_3053 # 3098870 # 3099307 # -1 # ID=1_3053;partial=00;start_type=ATG;rbs_motif=AGGA/GGAG/GAGG;rbs_spacer=11-12bp;gc_cont=0.447

	PROJECT ID	ACCESSION ID	ORGANISMS	CLASS	PROTEIN FUNCTION	PROTEIN ID	%IDENTITY
**Matched Family**	28537	CP001084	Lactobacillus casei str. Zhang, complete genome.	Lactobacillales	Molecular chaperone (small heat shock protein)	ADK19996	100.0

Input Sequence ATG-E1_Chromosome_2038 # 2048614 # 2049051 # -1 # ID=1_2038;partial=00;start_type=ATG;rbs_motif=AGGA;rbs_spacer=5-10bp;gc_cont=0.470

	PROJECT ID	ACCESSION ID	ORGANISMS	CLASS	PROTEIN FUNCTION	PROTEIN ID	%IDENTITY
**Matched Family**	28537	CP001084	Lactobacillus casei str. Zhang, complete genome.	Lactobacillales	conserved hypothetical protein	ADK19016	100.0

Input Sequence ATG-E1_Chromosome_1941 # 1952467 # 1952898 # -1 # ID=1_1941;partial=00;start_type=ATG;rbs_motif=None;rbs_spacer=None;gc_cont=0.505

	PROJECT ID	ACCESSION ID	ORGANISMS	CLASS	PROTEIN FUNCTION	PROTEIN ID	%IDENTITY
**Matched Family**	30359	FM177140	Lactobacillus casei BL23 complete genome, strain BL23.	Lactobacillales	Putative uncharacterized protein	CAQ67001	100.0

Input Sequence ATG-E1_Chromosome_1390 # 1381593 # 1382024 # 1 # ID=1_1390;partial=00;start_type=TTG;rbs_motif=AGGAGG;rbs_spacer=5-10bp;gc_cont=0.498

	PROJECT ID	ACCESSION ID	ORGANISMS	CLASS	PROTEIN FUNCTION	PROTEIN ID	%IDENTITY
**Matched Family**	28537	CP001084	Lactobacillus casei str. Zhang, complete genome.	Lactobacillales	F0F1-type ATP synthase, epsilon subunit	ADK18395	100.0

Input Sequence ATG-E1_Chromosome_2759 # 2795110 # 2795541 # -1 # ID=1_2759;partial=00;start_type=ATG;rbs_motif=GGA/GAG/AGG;rbs_spacer=5-10bp;gc_cont=0.454

	PROJECT ID	ACCESSION ID	ORGANISMS	CLASS	PROTEIN FUNCTION	PROTEIN ID	%IDENTITY
**Matched Family**	28537	CP001084	Lactobacillus casei str. Zhang, complete genome.	Lactobacillales	Septum formation initiator	ADK19698	100.0

Input Sequence: ATG-E1_Chromosome_581 # 600269 # 600697 # -1 # ID=1_581;partial=00;start_type=ATG;rbs_motif=GGAGG;rbs_spacer=5-10bp;gc_cont=0.466

	PROJECT ID	ACCESSION ID	ORGANISMS	CLASS	PROTEIN FUNCTION	PROTEIN ID	%IDENTITY
**Matched Family**	28537	CP001084	Lactobacillus casei str. Zhang, complete genome.	Lactobacillales	Predicted transcriptional regulator	ADK17764	100.0

Input Sequence: ATG-E1_Chromosome_566 # 583398 # 583826 # -1 # ID=1_566;partial=00;start_type=ATG;rbs_motif=AGGAGG;rbs_spacer=5-10bp;gc_cont=0.497

	PROJECT ID	ACCESSION ID	ORGANISMS	CLASS	PROTEIN FUNCTION	PROTEIN ID	%IDENTITY
**Matched Family**	28537	CP001084	Lactobacillus casei str. Zhang, complete genome.	Lactobacillales	Predicted redox protein, regulator of disulfide bond formation	ADK17747	100.0

Input Sequence: ATG-E1_Chromosome_2782 # 2822648 # 2823076 # 1 # ID=1_2782;partial=00;start_type=ATG;rbs_motif=AGGAGG;rbs_spacer=5-10bp;gc_cont=0.422

	PROJECT ID	ACCESSION ID	ORGANISMS	CLASS	PROTEIN FUNCTION	PROTEIN ID	%IDENTITY
**Matched Family**	28537	CP001084	Lactobacillus casei str. Zhang, complete genome.	Lactobacillales	conserved hypothetical protein	ADK19723	100.0

Input Sequence: ATG-E1_Chromosome_296 # 305982 # 306407 # 1 # ID=1_296;partial=00;start_type=ATG;rbs_motif=AGGAG;rbs_spacer=5-10bp;gc_cont=0.437

	PROJECT ID	ACCESSION ID	ORGANISMS	CLASS	PROTEIN FUNCTION	PROTEIN ID	%IDENTITY
**Matched Family**	28537	CP001084	Lactobacillus casei str. Zhang, complete genome.	Lactobacillales	conserved hypothetical protein	ADK17523	100.0

Input Sequence: ATG-E1_Chromosome_2084 # 2104637 # 2105062 # 1 # ID=1_2084;partial=00;start_type=ATG;rbs_motif=GGAG/GAGG;rbs_spacer=5-10bp;gc_cont=0.472

	PROJECT ID	ACCESSION ID	ORGANISMS	CLASS	PROTEIN FUNCTION	PROTEIN ID	%IDENTITY
**Matched Family**	28537	CP001084	Lactobacillus casei str. Zhang, complete genome.	Lactobacillales	hypothetical protein of possible phage origin	ADK19064	100.0

Input Sequence: ATG-E1_Chromosome_1240 # 1253156 # 1253578 # 1 # ID=1_1240;partial=00;start_type=ATG;rbs_motif=GGAG/GAGG;rbs_spacer=5-10bp;gc_cont=0.489

	PROJECT ID	ACCESSION ID	ORGANISMS	CLASS	PROTEIN FUNCTION	PROTEIN ID	%IDENTITY
**Matched Family**	28537	CP001084	Lactobacillus casei str. Zhang, complete genome.	Lactobacillales	conserved hypothetical protein	ADK18300	100.0

Input Sequence: ATG-E1_Chromosome_2053 # 2068697 # 2069119 # -1 # ID=1_2053;partial=00;start_type=ATG;rbs_motif=AGxAGG/AGGxGG;rbs_spacer=5-10bp;gc_cont=0.430

	PROJECT ID	ACCESSION ID	ORGANISMS	CLASS	PROTEIN FUNCTION	PROTEIN ID	%IDENTITY
**Matched Family**	28537	CP001084	Lactobacillus casei str. Zhang, complete genome.	Lactobacillales	conserved hypothetical protein	ADK19032	100.0

Input Sequence: ATG-E1_Chromosome_3066 # 3115170 # 3115589 # -1 # ID=1_3066;partial=00;start_type=TTG;rbs_motif=AGxAGG/AGGxGG;rbs_spacer=5-10bp;gc_cont=0.479

	PROJECT ID	ACCESSION ID	ORGANISMS	CLASS	PROTEIN FUNCTION	PROTEIN ID	%IDENTITY
**Matched Family**	28537	CP001084	Lactobacillus casei str. Zhang, complete genome.	Lactobacillales	conserved hypothetical protein	ADK20012	100.0

Input Sequence: ATG-E1_Chromosome_2848 # 2891784 # 2892200 # 1 # ID=1_2848;partial=00;start_type=ATG;rbs_motif=AGxAGG/AGGxGG;rbs_spacer=5-10bp;gc_cont=0.475

	PROJECT ID	ACCESSION ID	ORGANISMS	CLASS	PROTEIN FUNCTION	PROTEIN ID	%IDENTITY
**Matched Family**	28537	CP001084	Lactobacillus casei str. Zhang, complete genome.	Lactobacillales	Transcriptional regulator	ADK19793	100.0

Input Sequence: ATG-E1_Chromosome_2317 # 2348124 # 2348537 # -1 # ID=1_2317;partial=00;start_type=ATG;rbs_motif=GGAGG;rbs_spacer=5-10bp;gc_cont=0.459

	PROJECT ID	ACCESSION ID	ORGANISMS	CLASS	PROTEIN FUNCTION	PROTEIN ID	%IDENTITY
**Matched Family**	30359	FM177140	Lactobacillus casei BL23 complete genome, strain BL23.	Lactobacillales	Transcriptional regulator	CAQ67375	100.0

Input Sequence	ATG-E1_Chromosome_185 # 193787 # 194200 # 1 # ID=1_185;partial=00;start_type=GTG;rbs_motif=AGxAGG/AGGxGG;rbs_spacer=5-10bp;gc_cont=0.469					
	PROJECT ID	ACCESSION ID	ORGANISMS	CLASS	PROTEIN FUNCTION	PROTEIN ID	%IDENTITY
Matched Family	30359	FM177140	Lactobacillus casei BL23 complete genome, strain BL23.	Lactobacillales	NUDIX hydrolase	CAQ65237	100.0

Input Sequence	ATG-E1_Chromosome_295 # 305534 # 305947 # 1 # ID=1_295;partial=00;start_type=ATG;rbs_motif=AGxAGG/AGGxGG;rbs_spacer=5-10bp;gc_cont=0.444					
	PROJECT ID	ACCESSION ID	ORGANISMS	CLASS	PROTEIN FUNCTION	PROTEIN ID	%IDENTITY
Matched Family	28537	CP001084	Lactobacillus casei str. Zhang, complete genome.	Lactobacillales	conserved hypothetical protein	ADK17522	100.0

Input Sequence	ATG-E1_Chromosome_2460 # 2497969 # 2498382 # 1 # ID=1_2460;partial=00;start_type=ATG;rbs_motif=AGGAGG;rbs_spacer=5-10bp;gc_cont=0.449					
	PROJECT ID	ACCESSION ID	ORGANISMS	CLASS	PROTEIN FUNCTION	PROTEIN ID	%IDENTITY
Matched Family	28537	CP001084	Lactobacillus casei str. Zhang, complete genome.	Lactobacillales	conserved hypothetical protein	ADK19448	100.0

Input Sequence	ATG-E1_Chromosome_2989 # 3040417 # 3040830 # -1 # ID=1_2989;partial=00;start_type=ATG;rbs_motif=AGGAG;rbs_spacer=5-10bp;gc_cont=0.430					
	PROJECT ID	ACCESSION ID	ORGANISMS	CLASS	PROTEIN FUNCTION	PROTEIN ID	%IDENTITY
Matched Family	30359	FM177140	Lactobacillus casei BL23 complete genome, strain BL23.	Lactobacillales	AhaA protein (Fragment)	CAQ67991	100.0

Input Sequence	ATG-E1_Chromosome_1055 # 1067983 # 1068393 # 1 # ID=1_1055;partial=00;start_type=ATG;rbs_motif=AGGAGG;rbs_spacer=5-10bp;gc_cont=0.440					
	PROJECT ID	ACCESSION ID	ORGANISMS	CLASS	PROTEIN FUNCTION	PROTEIN ID	%IDENTITY
Matched Family	28537	CP001084	Lactobacillus casei str. Zhang, complete genome.	Lactobacillales	conserved hypothetical protein	ADK18104	100.0

Input Sequence	ATG-E1_Chromosome_790 # 809466 # 809873 # -1 # ID=1_790;partial=00;start_type=ATG;rbs_motif=AGxAGG/AGGxGG;rbs_spacer=5-10bp;gc_cont=0.473					
	PROJECT ID	ACCESSION ID	ORGANISMS	CLASS	PROTEIN FUNCTION	PROTEIN ID	%IDENTITY
Matched Family	28537	CP001084	Lactobacillus casei str. Zhang, complete genome.	Lactobacillales	conserved hypothetical protein	ADK17857	100.0

Input Sequence	ATG-E1_Chromosome_860 # 883358 # 883765 # -1 # ID=1_860;partial=00;start_type=ATG;rbs_motif=AGxAGG/AGGxGG;rbs_spacer=5-10bp;gc_cont=0.439					
	PROJECT ID	ACCESSION ID	ORGANISMS	CLASS	PROTEIN FUNCTION	PROTEIN ID	%IDENTITY
Matched Family	28537	CP001084	Lactobacillus casei str. Zhang, complete genome.	Lactobacillales	Conserved membrane protein, GtcA family	ADK17937	100.0

Input Sequence	ATG-E1_Chromosome_3054 # 3099467 # 3099871 # -1 # ID=1_3054;partial=00;start_type=ATG;rbs_motif=GGAG/GAGG;rbs_spacer=5-10bp;gc_cont=0.462					
	PROJECT ID	ACCESSION ID	ORGANISMS	CLASS	PROTEIN FUNCTION	PROTEIN ID	%IDENTITY
Matched Family	28537	CP001084	Lactobacillus casei str. Zhang, complete genome.	Lactobacillales	conserved hypothetical protein	ADK19997	100.0

Input Sequence	ATG-E1_Chromosome_1687 # 1686155 # 1686553 # 1 # ID=1_1687;partial=00;start_type=ATG;rbs_motif=AGGAG;rbs_spacer=5-10bp;gc_cont=0.491					
	PROJECT ID	ACCESSION ID	ORGANISMS	CLASS	PROTEIN FUNCTION	PROTEIN ID	%IDENTITY
Matched Family	28537	CP001084	Lactobacillus casei str. Zhang, complete genome.	Lactobacillales	Ribonuclease HI	ADK18684	100.0

Input Sequence	ATG-E1_Chromosome_454 # 463941 # 464339 # -1 # ID=1_454;partial=00;start_type=ATG;rbs_motif=AGGAG;rbs_spacer=5-10bp;gc_cont=0.411					
	PROJECT ID	ACCESSION ID	ORGANISMS	CLASS	PROTEIN FUNCTION	PROTEIN ID	%IDENTITY
Matched Family	30359	FM177140	Lactobacillus casei BL23 complete genome, strain BL23.	Lactobacillales	Putative uncharacterized protein	CAQ65487	100.0

Input Sequence	ATG-E1_Chromosome_1736 # 1741691 # 1742089 # -1 # ID=1_1736;partial=00;start_type=ATG;rbs_motif=None;rbs_spacer=None;gc_cont=0.454

	PROJECT ID	ACCESSION ID	ORGANISMS	CLASS	PROTEIN FUNCTION	PROTEIN ID	%IDENTITY
**Matched Family**	402	CP000423	Lactobacillus casei ATCC 334, complete genome.	Lactobacillales	diacylglycerol kinase	ABJ70292	100.0

**Input Sequence**	ATG-E1_Chromosome_1588 # 1580781 # 1581167 # 1 # ID=1_1588;partial=00;start_type=ATG;rbs_motif=GGAGG;rbs_spacer=5-10bp;gc_cont=0.421

	PROJECT ID	ACCESSION ID	ORGANISMS	CLASS	PROTEIN FUNCTION	PROTEIN ID	%IDENTITY
**Matched Family**	30359	FM177140	Lactobacillus casei BL23 complete genome, strain BL23.	Lactobacillales	Putative reductase	CAQ66670	100.0

**Input Sequence**	ATG-E1_Chromosome_2132 # 2146597 # 2146995 # -1 # ID=1_2132;partial=00;start_type=ATG;rbs_motif=GGA/GAG/AGG;rbs_spacer=5-10bp;gc_cont=0.451

	PROJECT ID	ACCESSION ID	ORGANISMS	CLASS	PROTEIN FUNCTION	PROTEIN ID	%IDENTITY
**Matched Family**	28537	CP001084	Lactobacillus casei str. Zhang, complete genome.	Lactobacillales	conserved hypothetical protein	ADK19110	100.0

**Input Sequence**	ATG-E1_Chromosome_2403 # 2443137 # 2443526 # 1 # ID=1_2403;partial=00;start_type=ATG;rbs_motif=GGxGG;rbs_spacer=3-4bp;gc_cont=0.469

	PROJECT ID	ACCESSION ID	ORGANISMS	CLASS	PROTEIN FUNCTION	PROTEIN ID	%IDENTITY
**Matched Family**	28537	CP001084	Lactobacillus casei str. Zhang, complete genome.	Lactobacillales	Transcriptional regulator, xre family	ADK19390	100.0

**Input Sequence**	ATG-E1_Chromosome_2479 # 2522594 # 2522980 # -1 # ID=1_2479;partial=00;start_type=TTG;rbs_motif=GGAGG;rbs_spacer=5-10bp;gc_cont=0.421

	PROJECT ID	ACCESSION ID	ORGANISMS	CLASS	PROTEIN FUNCTION	PROTEIN ID	%IDENTITY
**Matched Family**	30359	FM177140	Lactobacillus casei BL23 complete genome, strain BL23.	Lactobacillales	Putative uncharacterized protein	CAQ67534	100.0

**Input Sequence**	ATG-E1_Chromosome_1766 # 1766847 # 1767233 # -1 # ID=1_1766;partial=00;start_type=TTG;rbs_motif=GGA/GAG/AGG;rbs_spacer=5-10bp;gc_cont=0.452

	PROJECT ID	ACCESSION ID	ORGANISMS	CLASS	PROTEIN FUNCTION	PROTEIN ID	%IDENTITY
**Matched Family**	28537	CP001084	Lactobacillus casei str. Zhang, complete genome.	Lactobacillales	conserved hypothetical protein	ADK18764	100.0

**Input Sequence**	ATG-E1_Chromosome_1969 # 1980028 # 1980414 # 1 # ID=1_1969;partial=00;start_type=ATG;rbs_motif=AGGAG;rbs_spacer=5-10bp;gc_cont=0.447

	PROJECT ID	ACCESSION ID	ORGANISMS	CLASS	PROTEIN FUNCTION	PROTEIN ID	%IDENTITY
**Matched Family**	28537	CP001084	Lactobacillus casei str. Zhang, complete genome.	Lactobacillales	conserved hypothetical protein	ADK18947	100.0

**Input Sequence**	ATG-E1_Chromosome_302 # 310625 # 311005 # -1 # ID=1_302;partial=00;start_type=ATG;rbs_motif=GGAG/GAGG;rbs_spacer=5-10bp;gc_cont=0.402

	PROJECT ID	ACCESSION ID	ORGANISMS	CLASS	PROTEIN FUNCTION	PROTEIN ID	%IDENTITY
**Matched Family**	28537	CP001084	Lactobacillus casei str. Zhang, complete genome.	Lactobacillales	conserved hypothetical protein	ADK17529	100.0

**Input Sequence**	ATG-E1_Chromosome_863 # 886026 # 886406 # -1 # ID=1_863;partial=00;start_type=ATG;rbs_motif=AGGAG;rbs_spacer=5-10bp;gc_cont=0.438

	PROJECT ID	ACCESSION ID	ORGANISMS	CLASS	PROTEIN FUNCTION	PROTEIN ID	%IDENTITY
**Matched Family**	28537	CP001084	Lactobacillus casei str. Zhang, complete genome.	Lactobacillales	conserved hypothetical protein	ADK17940	100.0

**Input Sequence**	ATG-E1_Chromosome_1412 # 1397834 # 1398214 # 1 # ID=1_1412;partial=00;start_type=ATG;rbs_motif=AGGAGG;rbs_spacer=5-10bp;gc_cont=0.470

	PROJECT ID	ACCESSION ID	ORGANISMS	CLASS	PROTEIN FUNCTION	PROTEIN ID	%IDENTITY
**Matched Family**	28537	CP001084	Lactobacillus casei str. Zhang, complete genome.	Lactobacillales	conserved hypothetical protein	ADK18415	100.0

**Input Sequence**	ATG-E1_Chromosome_2530 # 2569805 # 2570182 # -1 # ID=1_2530;partial=00;start_type=ATG;rbs_motif=AGxAGG/AGGxGG;rbs_spacer=5-10bp;gc_cont=0.418

	PROJECT ID	ACCESSION ID	ORGANISMS	CLASS	PROTEIN FUNCTION	PROTEIN ID	%IDENTITY
**Matched**	28537	CP001084	Lactobacillus casei str. Zhang,	Lactobacillales	Predicted membrane protein	ADK19510	100.0

Family			complete genome.				

Input Sequence	ATG-E1_Chromosome_2518 # 2557550 # 2557924 # -1 # ID=1_2518;partial=00;start_type=GTG;rbs_motif=AGxAGG/AGGxGG;rbs_spacer=5-10bp;gc_cont=0.517						
	PROJECT ID	ACCESSION ID	ORGANISMS	CLASS	PROTEIN FUNCTION	PROTEIN ID	%IDENTITY
Matched Family	402	CP000423	Lactobacillus casei ATCC 334, complete genome.	Lactobacillales	transcriptional regulator, GntR family	ABJ71063	100.0

Input Sequence	ATG-E1_Chromosome_2774 # 2816283 # 2816657 # -1 # ID=1_2774;partial=00;start_type=ATG;rbs_motif=AGGAGG;rbs_spacer=5-10bp;gc_cont=0.456						
	PROJECT ID	ACCESSION ID	ORGANISMS	CLASS	PROTEIN FUNCTION	PROTEIN ID	%IDENTITY
Matched Family	28537	CP001084	Lactobacillus casei str. Zhang, complete genome.	Lactobacillales	Phosphopantetheinyl transferase (holo-ACP synthase)	ADK19715	100.0

Input Sequence	ATG-E1_Chromosome_1912 # 1927926 # 1928300 # -1 # ID=1_1912;partial=00;start_type=GTG;rbs_motif=GGAG/GAGG;rbs_spacer=5-10bp;gc_cont=0.483						
	PROJECT ID	ACCESSION ID	ORGANISMS	CLASS	PROTEIN FUNCTION	PROTEIN ID	%IDENTITY
Matched Family	28537	CP001084	Lactobacillus casei str. Zhang, complete genome.	Lactobacillales	Predicted transcriptional regulator	ADK18891	100.0

Input Sequence	ATG-E1_Chromosome_2052 # 2068183 # 2068557 # -1 # ID=1_2052;partial=00;start_type=ATG;rbs_motif=AGxAGG/AGGxGG;rbs_spacer=5-10bp;gc_cont=0.464						
	PROJECT ID	ACCESSION ID	ORGANISMS	CLASS	PROTEIN FUNCTION	PROTEIN ID	%IDENTITY
Matched Family	28537	CP001084	Lactobacillus casei str. Zhang, complete genome.	Lactobacillales	conserved hypothetical protein	ADK19031	100.0

Input Sequence	ATG-E1_Chromosome_1794 # 1807601 # 1807972 # 1 # ID=1_1794;partial=00;start_type=ATG;rbs_motif=AGGAGG;rbs_spacer=5-10bp;gc_cont=0.427						
	PROJECT ID	ACCESSION ID	ORGANISMS	CLASS	PROTEIN FUNCTION	PROTEIN ID	%IDENTITY
Matched Family	28537	CP001084	Lactobacillus casei str. Zhang, complete genome.	Lactobacillales	conserved hypothetical protein	ADK18776	100.0

Input Sequence	ATG-E1_Chromosome_1961 # 1970855 # 1971226 # 1 # ID=1_1961;partial=00;start_type=ATG;rbs_motif=GGAGG;rbs_spacer=5-10bp;gc_cont=0.452						
	PROJECT ID	ACCESSION ID	ORGANISMS	CLASS	PROTEIN FUNCTION	PROTEIN ID	%IDENTITY
Matched Family	402	CP000423	Lactobacillus casei ATCC 334, complete genome.	Lactobacillales	hypothetical protein	ABJ70494	100.0

Input Sequence	ATG-E1_Chromosome_717 # 726658 # 727026 # -1 # ID=1_717;partial=00;start_type=ATG;rbs_motif=GGAG/GAGG;rbs_spacer=5-10bp;gc_cont=0.363						
	PROJECT ID	ACCESSION ID	ORGANISMS	CLASS	PROTEIN FUNCTION	PROTEIN ID	%IDENTITY
Matched Family	402	CP000424	Lactobacillus casei ATCC 334 plasmid 1, complete sequence.	Lactobacillales	hypothetical protein	ABJ71636	100.0

Input Sequence	ATG-E1_Chromosome_2431 # 2472604 # 2472969 # -1 # ID=1_2431;partial=00;start_type=ATG;rbs_motif=AGxAGG/AGGxGG;rbs_spacer=5-10bp;gc_cont=0.503						
	PROJECT ID	ACCESSION ID	ORGANISMS	CLASS	PROTEIN FUNCTION	PROTEIN ID	%IDENTITY
Matched Family	28537	CP001084	Lactobacillus casei str. Zhang, complete genome.	Lactobacillales	Predicted transcriptional regulator	ADK19422	100.0

Input Sequence	ATG-E1_Chromosome_2 # 1301 # 1666 # -1 # ID=1_2;partial=00;start_type=ATG;rbs_motif=AGGA;rbs_spacer=5-10bp;gc_cont=0.437						
	PROJECT ID	ACCESSION ID	ORGANISMS	CLASS	PROTEIN FUNCTION	PROTEIN ID	%IDENTITY
Matched Family	28537	CP001084	Lactobacillus casei str. Zhang, complete genome.	Lactobacillales	conserved hypothetical protein	ADK20025	100.0

Input Sequence	ATG-E1_Chromosome_1488 # 1472942 # 1473307 # 1 # ID=1_1488;partial=00;start_type=ATG;rbs_motif=GGA/GAG/AGG;rbs_spacer=5-10bp;gc_cont=0.448						
	PROJECT ID	ACCESSION ID	ORGANISMS	CLASS	PROTEIN FUNCTION	PROTEIN ID	%IDENTITY
Matched Family	28537	CP001084	Lactobacillus casei str. Zhang, complete genome.	Lactobacillales	Protein required for the initiation of cell division	ADK18493	100.0

Input Sequence: ATG-E1_Chromosome_2406 # 2446009 # 2446374 # 1 # ID=1_2406;partial=00;start_type=TTG;rbs_motif=GGAG/GAGG;rbs_spacer=5-10bp;gc_cont=0.418

	PROJECT ID	ACCESSION ID	ORGANISMS	CLASS	PROTEIN FUNCTION	PROTEIN ID	%IDENTITY
**Matched Family**	28537	CP001084	Lactobacillus casei str. Zhang, complete genome.	Lactobacillales	conserved hypothetical protein	ADK19393	100.0

Input Sequence: ATG-E1_Chromosome_2600 # 2640348 # 2640713 # 1 # ID=1_2600;partial=00;start_type=TTG;rbs_motif=AGGAG;rbs_spacer=5-10bp;gc_cont=0.462

	PROJECT ID	ACCESSION ID	ORGANISMS	CLASS	PROTEIN FUNCTION	PROTEIN ID	%IDENTITY
**Matched Family**	30359	FM177140	Lactobacillus casei BL23 complete genome, strain BL23.	Lactobacillales	Putative uncharacterized protein	CAQ67651	100.0

Input Sequence: ATG-E1_Chromosome_231 # 243298 # 243660 # -1 # ID=1_231;partial=00;start_type=GTG;rbs_motif=GGAGG;rbs_spacer=5-10bp;gc_cont=0.471

	PROJECT ID	ACCESSION ID	ORGANISMS	CLASS	PROTEIN FUNCTION	PROTEIN ID	%IDENTITY
**Matched Family**	28537	CP001084	Lactobacillus casei str. Zhang, complete genome.	Lactobacillales	Predicted membrane protein	ADK17458	100.0

Input Sequence: ATG-E1_Chromosome_1891 # 1904982 # 1905344 # -1 # ID=1_1891;partial=00;start_type=ATG;rbs_motif=AGGAGG;rbs_spacer=5-10bp;gc_cont=0.501

	PROJECT ID	ACCESSION ID	ORGANISMS	CLASS	PROTEIN FUNCTION	PROTEIN ID	%IDENTITY
**Matched Family**	28537	CP001084	Lactobacillus casei str. Zhang, complete genome.	Lactobacillales	glutamine synthetase repressor	ADK18871	100.0

Input Sequence: ATG-E1_Chromosome_2367 # 2402444 # 2402800 # 1 # ID=1_2367;partial=00;start_type=ATG;rbs_motif=GGAG/GAGG;rbs_spacer=5-10bp;gc_cont=0.429

	PROJECT ID	ACCESSION ID	ORGANISMS	CLASS	PROTEIN FUNCTION	PROTEIN ID	%IDENTITY
**Matched Family**	402	CP000423	Lactobacillus casei ATCC 334, complete genome.	Lactobacillales	hypothetical protein	ABJ70932	100.0

Input Sequence: ATG-E1_Chromosome_1236 # 1249758 # 1250114 # -1 # ID=1_1236;partial=00;start_type=ATG;rbs_motif=GGAGG;rbs_spacer=5-10bp;gc_cont=0.507

	PROJECT ID	ACCESSION ID	ORGANISMS	CLASS	PROTEIN FUNCTION	PROTEIN ID	%IDENTITY
**Matched Family**	28537	CP001084	Lactobacillus casei str. Zhang, complete genome.	Lactobacillales	conserved hypothetical protein	ADK18296	100.0

Input Sequence: ATG-E1_Chromosome_2624 # 2666368 # 2666724 # -1 # ID=1_2624;partial=00;start_type=ATG;rbs_motif=AGxAGG/AGGxGG;rbs_spacer=11-12bp;gc_cont=0.510

	PROJECT ID	ACCESSION ID	ORGANISMS	CLASS	PROTEIN FUNCTION	PROTEIN ID	%IDENTITY
**Matched Family**	28537	CP001084	Lactobacillus casei str. Zhang, complete genome.	Lactobacillales	Integral membrane protein for chromosome condensation	ADK19614	100.0

Input Sequence: ATG-E1_Chromosome_625 # 654931 # 655287 # 1 # ID=1_625;partial=00;start_type=ATG;rbs_motif=AGGAG;rbs_spacer=5-10bp;gc_cont=0.448

	PROJECT ID	ACCESSION ID	ORGANISMS	CLASS	PROTEIN FUNCTION	PROTEIN ID	%IDENTITY
**Matched Family**	30359	FM177140	Lactobacillus casei BL23 complete genome, strain BL23.	Lactobacillales	Putative uncharacterized protein	CAQ65686	100.0

Input Sequence: ATG-E1_Chromosome_1004 # 1018567 # 1018920 # 1 # ID=1_1004;partial=00;start_type=ATG;rbs_motif=GGAGG;rbs_spacer=5-10bp;gc_cont=0.477

	PROJECT ID	ACCESSION ID	ORGANISMS	CLASS	PROTEIN FUNCTION	PROTEIN ID	%IDENTITY
**Matched Family**	28537	CP001084	Lactobacillus casei str. Zhang, complete genome.	Lactobacillales	conserved hypothetical protein	ADK18062	100.0

Input Sequence: ATG-E1_Chromosome_1642 # 1639807 # 1640160 # 1 # ID=1_1642;partial=00;start_type=GTG;rbs_motif=AGxAGG/AGGxGG;rbs_spacer=5-10bp;gc_cont=0.477

	PROJECT ID	ACCESSION ID	ORGANISMS	CLASS	PROTEIN FUNCTION	PROTEIN ID	%IDENTITY
**Matched Family**	28537	CP001084	Lactobacillus casei str. Zhang, complete genome.	Lactobacillales	conserved hypothetical protein	ADK18637	100.0

Input Sequence	ATG-E1_Chromosome_618 # 650676 # 651029 # -1 # ID=1_618;partial=00;start_type=ATG;rbs_motif=GGAG/GAGG;rbs_spacer=5-10bp;gc_cont=0.407					
	PROJECT ID	ACCESSION ID	ORGANISMS	CLASS	PROTEIN FUNCTION	PROTEIN ID	%IDENTITY
Matched Family	30359	FM177140	Lactobacillus casei BL23 complete genome, strain BL23.	Lactobacillales	Rep protein	CAQ65680	100.0

Input Sequence	ATG-E1_Chromosome_914 # 926974 # 927324 # 1 # ID=1_914;partial=00;start_type=ATG;rbs_motif=AGGA/GGAG/GAGG;rbs_spacer=11-12bp;gc_cont=0.456						
	PROJECT ID	ACCESSION ID	ORGANISMS	CLASS	PROTEIN FUNCTION	PROTEIN ID	%IDENTITY
Matched Family	28537	CP001084	Lactobacillus casei str. Zhang, complete genome.	Lactobacillales	conserved hypothetical protein	ADK17974	100.0

Input Sequence	ATG-E1_Chromosome_2569 # 2612025 # 2612336 # -1 # ID=1_2569;partial=00;start_type=ATG;rbs_motif=AGGA;rbs_spacer=5-10bp;gc_cont=0.478						
	PROJECT ID	ACCESSION ID	ORGANISMS	CLASS	PROTEIN FUNCTION	PROTEIN ID	%IDENTITY
Matched Family	30359	FM177140	Lactobacillus casei BL23 complete genome, strain BL23.	Lactobacillales	Putative uncharacterized protein	CAQ67622	100.0

Input Sequence	ATG-E1_Chromosome_920 # 932324 # 932671 # 1 # ID=1_920;partial=00;start_type=ATG;rbs_motif=GGAGG;rbs_spacer=5-10bp;gc_cont=0.480						
	PROJECT ID	ACCESSION ID	ORGANISMS	CLASS	PROTEIN FUNCTION	PROTEIN ID	%IDENTITY
Matched Family	28537	CP001084	Lactobacillus casei str. Zhang, complete genome.	Lactobacillales	conserved hypothetical protein	ADK17979	100.0

Input Sequence	ATG-E1_Chromosome_1964 # 1973775 # 1974119 # -1 # ID=1_1964;partial=00;start_type=ATG;rbs_motif=GGAG/GAGG;rbs_spacer=5-10bp;gc_cont=0.420						
	PROJECT ID	ACCESSION ID	ORGANISMS	CLASS	PROTEIN FUNCTION	PROTEIN ID	%IDENTITY
Matched Family	28537	CP001084	Lactobacillus casei str. Zhang, complete genome.	Lactobacillales	conserved hypothetical protein	ADK18942	100.0

Input Sequence	ATG-E1_Chromosome_334 # 345405 # 345749 # 1 # ID=1_334;partial=00;start_type=ATG;rbs_motif=AGGA/GGAG/GAGG;rbs_spacer=11-12bp;gc_cont=0.432						
	PROJECT ID	ACCESSION ID	ORGANISMS	CLASS	PROTEIN FUNCTION	PROTEIN ID	%IDENTITY
Matched Family	28537	CP001084	Lactobacillus casei str. Zhang, complete genome.	Lactobacillales	conserved hypothetical protein	ADK17561	100.0

Input Sequence	ATG-E1_Chromosome_2424 # 2465782 # 2466126 # -1 # ID=1_2424;partial=00;start_type=ATG;rbs_motif=GGA/GAG/AGG;rbs_spacer=5-10bp;gc_cont=0.406						
	PROJECT ID	ACCESSION ID	ORGANISMS	CLASS	PROTEIN FUNCTION	PROTEIN ID	%IDENTITY
Matched Family	28537	CP001084	Lactobacillus casei str. Zhang, complete genome.	Lactobacillales	conserved hypothetical protein	ADK19415	100.0

Input Sequence	ATG-E1_Chromosome_2852 # 2897186 # 2897530 # -1 # ID=1_2852;partial=00;start_type=ATG;rbs_motif=AGGA/GGAG/GAGG;rbs_spacer=11-12bp;gc_cont=0.432						
	PROJECT ID	ACCESSION ID	ORGANISMS	CLASS	PROTEIN FUNCTION	PROTEIN ID	%IDENTITY
Matched Family	28537	CP001084	Lactobacillus casei str. Zhang, complete genome.	Lactobacillales	conserved hypothetical protein	ADK19798	100.0

Input Sequence	ATG-E1_Chromosome_881 # 898421 # 898762 # 1 # ID=1_881;partial=00;start_type=ATG;rbs_motif=AGGAG;rbs_spacer=5-10bp;gc_cont=0.392						
	PROJECT ID	ACCESSION ID	ORGANISMS	CLASS	PROTEIN FUNCTION	PROTEIN ID	%IDENTITY
Matched Family	28537	CP001084	Lactobacillus casei str. Zhang, complete genome.	Lactobacillales	hypothetical protein	ADK18249	100.0

Input Sequence	ATG-E1_Chromosome_1511 # 1496238 # 1496579 # 1 # ID=1_1511;partial=00;start_type=ATG;rbs_motif=GGAGG;rbs_spacer=5-10bp;gc_cont=0.418						
	PROJECT ID	ACCESSION ID	ORGANISMS	CLASS	PROTEIN FUNCTION	PROTEIN ID	%IDENTITY
Matched Family	28537	CP001084	Lactobacillus casei str. Zhang, complete genome.	Lactobacillales	conserved hypothetical protein	ADK18516	100.0

Input Sequence	ATG-E1_Chromosome_2102 # 2118813 # 2119151 # 1 # ID=1_2102;partial=00;start_type=ATG;rbs_motif=GGAGG;rbs_spacer=5-10bp;gc_cont=0.496

	PROJECT ID	ACCESSION ID	ORGANISMS	CLASS	PROTEIN FUNCTION	PROTEIN ID	%IDENTITY
**Matched Family**	28537	CP001084	Lactobacillus casei str. Zhang, complete genome.	Lactobacillales	Predicted transcriptional regulator	ADK19082	100.0

Input Sequence: ATG-E1_Chromosome_2795 # 2835128 # 2835466 # -1 # ID=1_2795;partial=00;start_type=ATG;rbs_motif=AGxAGG/AGGxGG;rbs_spacer=11-12bp;gc_cont=0.431

	PROJECT ID	ACCESSION ID	ORGANISMS	CLASS	PROTEIN FUNCTION	PROTEIN ID	%IDENTITY
**Matched Family**	402	CP000423	Lactobacillus casei ATCC 334, complete genome.	Lactobacillales	hypothetical protein	ABJ71305	100.0

Input Sequence: ATG-E1_Chromosome_17 # 15173 # 15511 # -1 # ID=1_17;partial=00;start_type=ATG;rbs_motif=GGxGG;rbs_spacer=5-10bp;gc_cont=0.448

	PROJECT ID	ACCESSION ID	ORGANISMS	CLASS	PROTEIN FUNCTION	PROTEIN ID	%IDENTITY
**Matched Family**	28537	CP001084	Lactobacillus casei str. Zhang, complete genome.	Lactobacillales	conserved hypothetical protein	ADK20040	100.0

Input Sequence: ATG-E1_Chromosome_1210 # 1220875 # 1221210 # -1 # ID=1_1210;partial=00;start_type=ATG;rbs_motif=AGGAGG;rbs_spacer=5-10bp;gc_cont=0.473

	PROJECT ID	ACCESSION ID	ORGANISMS	CLASS	PROTEIN FUNCTION	PROTEIN ID	%IDENTITY
**Matched Family**	28537	CP001084	Lactobacillus casei str. Zhang, complete genome.	Lactobacillales	acetyl-CoA carboxylase, carboxyltransferase component-like protein	ADK18269	100.0

Input Sequence: ATG-E1_Chromosome_2586 # 2626953 # 2627288 # -1 # ID=1_2586;partial=00;start_type=ATG;rbs_motif=AGGA;rbs_spacer=5-10bp;gc_cont=0.485

	PROJECT ID	ACCESSION ID	ORGANISMS	CLASS	PROTEIN FUNCTION	PROTEIN ID	%IDENTITY
**Matched Family**	30359	FM177140	Lactobacillus casei BL23 complete genome, strain BL23.	Lactobacillales	Prebacteriocin	CAQ67638	100.0

Input Sequence: ATG-E1_Chromosome_1368 # 1358253 # 1358588 # -1 # ID=1_1368;partial=00;start_type=GTG;rbs_motif=GGAGG;rbs_spacer=5-10bp;gc_cont=0.461

	PROJECT ID	ACCESSION ID	ORGANISMS	CLASS	PROTEIN FUNCTION	PROTEIN ID	%IDENTITY
**Matched Family**	28537	CP001084	Lactobacillus casei str. Zhang, complete genome.	Lactobacillales	conserved hypothetical protein	ADK18372	100.0

Input Sequence: ATG-E1_Chromosome_1574 # 1564302 # 1564637 # 1 # ID=1_1574;partial=00;start_type=ATG;rbs_motif=GGA/GAG/AGG;rbs_spacer=5-10bp;gc_cont=0.435

	PROJECT ID	ACCESSION ID	ORGANISMS	CLASS	PROTEIN FUNCTION	PROTEIN ID	%IDENTITY
**Matched Family**	28537	CP001084	Lactobacillus casei str. Zhang, complete genome.	Lactobacillales	Uncharacterized integral membrane protein	ADK18576	100.0

Input Sequence: ATG-E1_Chromosome_2217 # 2237497 # 2237832 # -1 # ID=1_2217;partial=00;start_type=ATG;rbs_motif=AGGAGG;rbs_spacer=5-10bp;gc_cont=0.435

	PROJECT ID	ACCESSION ID	ORGANISMS	CLASS	PROTEIN FUNCTION	PROTEIN ID	%IDENTITY
**Matched Family**	28537	CP001084	Lactobacillus casei str. Zhang, complete genome.	Lactobacillales	Predicted transcriptional regulator	ADK19182	100.0

Input Sequence: ATG-E1_Chromosome_644 # 666408 # 666743 # 1 # ID=1_644;partial=00;start_type=ATG;rbs_motif=GGA/GAG/AGG;rbs_spacer=5-10bp;gc_cont=0.473

	PROJECT ID	ACCESSION ID	ORGANISMS	CLASS	PROTEIN FUNCTION	PROTEIN ID	%IDENTITY
**Matched Family**	402	CP000423	Lactobacillus casei ATCC 334, complete genome.	Lactobacillales	hypothetical protein	ABJ69370	100.0

Input Sequence: ATG-E1_Chromosome_659 # 679726 # 680061 # 1 # ID=1_659;partial=00;start_type=ATG;rbs_motif=AGGAGG;rbs_spacer=5-10bp;gc_cont=0.440

	PROJECT ID	ACCESSION ID	ORGANISMS	CLASS	PROTEIN FUNCTION	PROTEIN ID	%IDENTITY
**Matched Family**	30359	FM177140	Lactobacillus casei BL23 complete genome, strain BL23.	Lactobacillales	Putative uncharacterized protein	CAQ65718	100.0

Input Sequence: ATG-E1_Chromosome_2605 # 2644915 # 2645247 # 1 # ID=1_2605;partial=00;start_type=ATG;rbs_motif=GGAGG;rbs_spacer=5-10bp;gc_cont=0.502

	PROJECT ID	ACCESSION ID	ORGANISMS	CLASS	PROTEIN FUNCTION	PROTEIN ID	%IDENTITY
**Matched**	28537	CP001084	Lactobacillus casei str. Zhang,	Lactobacillales	Predicted transcriptional regulator	ADK19593	100.0

Family			complete genome.				

Input Sequence	ATG-E1_Chromosome_347 # 357950 # 358282 # 1 # ID=1_347;partial=00;start_type=ATG;rbs_motif=GGAG/GAGG;rbs_spacer=5-10bp;gc_cont=0.468						
	PROJECT ID	ACCESSION ID	ORGANISMS	CLASS	PROTEIN FUNCTION	PROTEIN ID	%IDENTITY
Matched Family	28537	CP001084	Lactobacillus casei str. Zhang, complete genome.	Lactobacillales	conserved hypothetical protein	ADK17573	100.0

Input Sequence	ATG-E1_Chromosome_522 # 532688 # 533020 # 1 # ID=1_522;partial=00;start_type=ATG;rbs_motif=AGxAGG/AGGxGG;rbs_spacer=5-10bp;gc_cont=0.444						
	PROJECT ID	ACCESSION ID	ORGANISMS	CLASS	PROTEIN FUNCTION	PROTEIN ID	%IDENTITY
Matched Family	28537	CP001084	Lactobacillus casei str. Zhang, complete genome.	Lactobacillales	conserved hypothetical protein	ADK17698	100.0

Input Sequence	ATG-E1_Chromosome_1485 # 1471047 # 1471379 # 1 # ID=1_1485;partial=00;start_type=ATG;rbs_motif=GGAGG;rbs_spacer=5-10bp;gc_cont=0.432						
	PROJECT ID	ACCESSION ID	ORGANISMS	CLASS	PROTEIN FUNCTION	PROTEIN ID	%IDENTITY
Matched Family	28537	CP001084	Lactobacillus casei str. Zhang, complete genome.	Lactobacillales	conserved hypothetical protein	ADK18490	100.0

Input Sequence	ATG-E1_Chromosome_2583 # 2624805 # 2625131 # -1 # ID=1_2583;partial=00;start_type=ATG;rbs_motif=GGA/GAG/AGG;rbs_spacer=5-10bp;gc_cont=0.410						
	PROJECT ID	ACCESSION ID	ORGANISMS	CLASS	PROTEIN FUNCTION	PROTEIN ID	%IDENTITY
Matched Family	402	CP000423	Lactobacillus casei ATCC 334, complete genome.	Lactobacillales	hypothetical protein	ABJ71137	100.0

Input Sequence	ATG-E1_Chromosome_257 # 268368 # 268697 # -1 # ID=1_257;partial=00;start_type=ATG;rbs_motif=AGGAGG;rbs_spacer=5-10bp;gc_cont=0.427						
	PROJECT ID	ACCESSION ID	ORGANISMS	CLASS	PROTEIN FUNCTION	PROTEIN ID	%IDENTITY
Matched Family	30359	FM177140	Lactobacillus casei BL23 complete genome, strain BL23.	Lactobacillales	Putative uncharacterized protein	CAQ65290	100.0

Input Sequence	ATG-E1_Chromosome_2457 # 2493995 # 2494324 # -1 # ID=1_2457;partial=00;start_type=ATG;rbs_motif=AGGAG;rbs_spacer=5-10bp;gc_cont=0.503						
	PROJECT ID	ACCESSION ID	ORGANISMS	CLASS	PROTEIN FUNCTION	PROTEIN ID	%IDENTITY
Matched Family	28537	CP001084	Lactobacillus casei str. Zhang, complete genome.	Lactobacillales	conserved hypothetical protein	ADK19445	100.0

Input Sequence	ATG-E1_Chromosome_1167 # 1189332 # 1189655 # 1 # ID=1_1167;partial=00;start_type=ATG;rbs_motif=GGAGG;rbs_spacer=5-10bp;gc_cont=0.423						
	PROJECT ID	ACCESSION ID	ORGANISMS	CLASS	PROTEIN FUNCTION	PROTEIN ID	%IDENTITY
Matched Family	30359	FM177140	Lactobacillus casei BL23 complete genome, strain BL23.	Lactobacillales	Competence protein ComGC	CAQ66274	100.0

Input Sequence	ATG-E1_Chromosome_1690 # 1689244 # 1689570 # -1 # ID=1_1690;partial=00;start_type=ATG;rbs_motif=GGAG/GAGG;rbs_spacer=5-10bp;gc_cont=0.477						
	PROJECT ID	ACCESSION ID	ORGANISMS	CLASS	PROTEIN FUNCTION	PROTEIN ID	%IDENTITY
Matched Family	30359	FM177140	Lactobacillus casei BL23 complete genome, strain BL23.	Lactobacillales	Putative uncharacterized protein	CAQ66770	100.0

Input Sequence	ATG-E1_Chromosome_2389 # 2430067 # 2430390 # 1 # ID=1_2389;partial=00;start_type=ATG;rbs_motif=AGGAG;rbs_spacer=5-10bp;gc_cont=0.435						
	PROJECT ID	ACCESSION ID	ORGANISMS	CLASS	PROTEIN FUNCTION	PROTEIN ID	%IDENTITY
Matched Family	28537	CP001084	Lactobacillus casei str. Zhang, complete genome.	Lactobacillales	conserved hypothetical protein	ADK19376	100.0

Input Sequence	ATG-E1_Chromosome_234 # 245975 # 246298 # 1 # ID=1_234;partial=00;start_type=ATG;rbs_motif=GGAG/GAGG;rbs_spacer=5-10bp;gc_cont=0.497						
	PROJECT ID	ACCESSION ID	ORGANISMS	CLASS	PROTEIN FUNCTION	PROTEIN ID	%IDENTITY
Matched Family	402	CP000423	Lactobacillus casei ATCC 334, complete genome.	Lactobacillales	hypothetical protein	ABJ69016	100.0

Input Sequence	ATG-E1_Chromosome_1757 # 1760955 # 1761278 # -1 # ID=1_1757;partial=00;start_type=ATG;rbs_motif=AGGAG/GGAGG;rbs_spacer=11-12bp;gc_cont=0.423					
	PROJECT ID	ACCESSION ID	ORGANISMS	CLASS	PROTEIN FUNCTION	PROTEIN ID	%IDENTITY
Matched Family	28537	CP001084	Lactobacillus casei str. Zhang, complete genome.	Lactobacillales	conserved hypothetical protein	ADK18755	100.0

Input Sequence	ATG-E1_Chromosome_18 # 15669 # 15992 # 1 # ID=1_18;partial=00;start_type=ATG;rbs_motif=GGAG/GAGG;rbs_spacer=5-10bp;gc_cont=0.441						
	PROJECT ID	ACCESSION ID	ORGANISMS	CLASS	PROTEIN FUNCTION	PROTEIN ID	%IDENTITY
Matched Family	28537	CP001084	Lactobacillus casei str. Zhang, complete genome.	Lactobacillales	conserved hypothetical protein	ADK20041	100.0

Input Sequence	ATG-E1_Chromosome_952 # 965780 # 966100 # 1 # ID=1_952;partial=00;start_type=ATG;rbs_motif=AGGAG;rbs_spacer=5-10bp;gc_cont=0.442						
	PROJECT ID	ACCESSION ID	ORGANISMS	CLASS	PROTEIN FUNCTION	PROTEIN ID	%IDENTITY
Matched Family	28537	CP001084	Lactobacillus casei str. Zhang, complete genome.	Lactobacillales	conserved hypothetical protein	ADK18016	100.0

Input Sequence	ATG-E1_Chromosome_1954 # 1965668 # 1965985 # -1 # ID=1_1954;partial=00;start_type=ATG;rbs_motif=AGGA;rbs_spacer=5-10bp;gc_cont=0.390						
	PROJECT ID	ACCESSION ID	ORGANISMS	CLASS	PROTEIN FUNCTION	PROTEIN ID	%IDENTITY
Matched Family	28537	CP001084	Lactobacillus casei str. Zhang, complete genome.	Lactobacillales	Thiol-disulfide isomerase and thioredoxin	ADK18932	100.0

Input Sequence	ATG-E1_Chromosome_2056 # 2072452 # 2072769 # -1 # ID=1_2056;partial=00;start_type=ATG;rbs_motif=AGGAG;rbs_spacer=5-10bp;gc_cont=0.478						
	PROJECT ID	ACCESSION ID	ORGANISMS	CLASS	PROTEIN FUNCTION	PROTEIN ID	%IDENTITY
Matched Family	28537	CP001084	Lactobacillus casei str. Zhang, complete genome.	Lactobacillales	Glutaredoxin related protein	ADK19036	100.0

Input Sequence	ATG-E1_Chromosome_979 # 993962 # 994276 # 1 # ID=1_979;partial=00;start_type=ATG;rbs_motif=AGGAG;rbs_spacer=5-10bp;gc_cont=0.441						
	PROJECT ID	ACCESSION ID	ORGANISMS	CLASS	PROTEIN FUNCTION	PROTEIN ID	%IDENTITY
Matched Family	28537	CP001084	Lactobacillus casei str. Zhang, complete genome.	Lactobacillales	conserved hypothetical protein	ADK18043	100.0

Input Sequence	ATG-E1_Chromosome_1926 # 1940387 # 1940698 # -1 # ID=1_1926;partial=00;start_type=ATG;rbs_motif=AGGAG;rbs_spacer=5-10bp;gc_cont=0.474						
	PROJECT ID	ACCESSION ID	ORGANISMS	CLASS	PROTEIN FUNCTION	PROTEIN ID	%IDENTITY
Matched Family	28537	CP001084	Lactobacillus casei str. Zhang, complete genome.	Lactobacillales	RNA-binding protein, KH domain	ADK18906	100.0

Input Sequence	ATG-E1_Chromosome_442 # 452253 # 452564 # 1 # ID=1_442;partial=00;start_type=ATG;rbs_motif=AGGAG;rbs_spacer=5-10bp;gc_cont=0.442						
	PROJECT ID	ACCESSION ID	ORGANISMS	CLASS	PROTEIN FUNCTION	PROTEIN ID	%IDENTITY
Matched Family	402	CP000423	Lactobacillus casei ATCC 334, complete genome.	Lactobacillales	Phosphotransferase system, galactitol-specific IIB component	ABJ69185	100.0

Input Sequence	ATG-E1_Chromosome_1732 # 1739059 # 1739370 # -1 # ID=1_1732;partial=00;start_type=ATG;rbs_motif=AGGAG;rbs_spacer=5-10bp;gc_cont=0.429						
	PROJECT ID	ACCESSION ID	ORGANISMS	CLASS	PROTEIN FUNCTION	PROTEIN ID	%IDENTITY
Matched Family	28537	CP001084	Lactobacillus casei str. Zhang, complete genome.	Lactobacillales	conserved hypothetical protein	ADK18731	100.0

Input Sequence	ATG-E1_Chromosome_2256 # 2279194 # 2279505 # -1 # ID=1_2256;partial=00;start_type=ATG;rbs_motif=AGxAGG/AGGxGG;rbs_spacer=5-10bp;gc_cont=0.478						
	PROJECT ID	ACCESSION ID	ORGANISMS	CLASS	PROTEIN FUNCTION	PROTEIN ID	%IDENTITY
Matched Family	28537	CP001084	Lactobacillus casei str. Zhang, complete genome.	Lactobacillales	Transcriptional regulator, xre family	ADK19230	100.0

Input	ATG-E1_Chromosome_1180 # 1200890 # 1201198 # -1 # ID=1_1180;partial=00;start_type=ATG;rbs_motif=AGGAGG;rbs_spacer=5-

Sequence	10bp;gc_cont=0.437						
	PROJECT ID	ACCESSION ID	ORGANISMS	CLASS	PROTEIN FUNCTION	PROTEIN ID	%IDENTITY
Matched Family	28537	CP001084	Lactobacillus casei str. Zhang, complete genome.	Lactobacillales	conserved hypothetical protein	ADK18237	100.0

Input Sequence	ATG-E1_Chromosome_2588 # 2628274 # 2628582 # -1 # ID=1_2588;partial=00;start_type=ATG;rbs_motif=GGAG/GAGG;rbs_spacer=5-10bp;gc_cont=0.350						
	PROJECT ID	ACCESSION ID	ORGANISMS	CLASS	PROTEIN FUNCTION	PROTEIN ID	%IDENTITY
Matched Family	28537	CP001084	Lactobacillus casei str. Zhang, complete genome.	Lactobacillales	conserved hypothetical protein	ADK19577	100.0

Input Sequence	ATG-E1_Chromosome_892 # 908005 # 908310 # -1 # ID=1_892;partial=00;start_type=ATG;rbs_motif=AGGAGG;rbs_spacer=5-10bp;gc_cont=0.441						
	PROJECT ID	ACCESSION ID	ORGANISMS	CLASS	PROTEIN FUNCTION	PROTEIN ID	%IDENTITY
Matched Family	28537	CP001084	Lactobacillus casei str. Zhang, complete genome.	Lactobacillales	Small conserved membrane protein	ADK17948	100.0

Input Sequence	ATG-E1_Chromosome_286 # 300776 # 301081 # 1 # ID=1_286;partial=00;start_type=ATG;rbs_motif=GGA/GAG/AGG;rbs_spacer=5-10bp;gc_cont=0.422						
	PROJECT ID	ACCESSION ID	ORGANISMS	CLASS	PROTEIN FUNCTION	PROTEIN ID	%IDENTITY
Matched Family	28537	CP001084	Lactobacillus casei str. Zhang, complete genome.	Lactobacillales	conserved hypothetical protein	ADK17513	100.0

Input Sequence	ATG-E1_Chromosome_1126 # 1148263 # 1148565 # 1 # ID=1_1126;partial=00;start_type=ATG;rbs_motif=GGAG/GAGG;rbs_spacer=5-10bp;gc_cont=0.363						
	PROJECT ID	ACCESSION ID	ORGANISMS	CLASS	PROTEIN FUNCTION	PROTEIN ID	%IDENTITY
Matched Family	402	CP000423	Lactobacillus casei ATCC 334, complete genome.	Lactobacillales	hypothetical protein	ABJ69781	100.0

Input Sequence	ATG-E1_Chromosome_2027 # 2039147 # 2039446 # 1 # ID=1_2027;partial=00;start_type=ATG;rbs_motif=AGxAGG/AGGxGG;rbs_spacer=5-10bp;gc_cont=0.443						
	PROJECT ID	ACCESSION ID	ORGANISMS	CLASS	PROTEIN FUNCTION	PROTEIN ID	%IDENTITY
Matched Family	28537	CP001084	Lactobacillus casei str. Zhang, complete genome.	Lactobacillales	conserved hypothetical protein	ADK19005	100.0

Input Sequence	ATG-E1_Chromosome_643 # 666143 # 666421 # 1 # ID=1_643;partial=00;start_type=ATG;rbs_motif=3Base/5BMM;rbs_spacer=13-15bp;gc_cont=0.430						
	PROJECT ID	ACCESSION ID	ORGANISMS	CLASS	PROTEIN FUNCTION	PROTEIN ID	%IDENTITY
Matched Family	30359	FM177140	Lactobacillus casei BL23 complete genome, strain BL23.	Lactobacillales	Putative uncharacterized protein	CAQ65702	100.0

Input Sequence	ATG-E1_Chromosome_1220 # 1231300 # 1231596 # 1 # ID=1_1220;partial=00;start_type=ATG;rbs_motif=GGA/GAG/AGG;rbs_spacer=3-4bp;gc_cont=0.481						
	PROJECT ID	ACCESSION ID	ORGANISMS	CLASS	PROTEIN FUNCTION	PROTEIN ID	%IDENTITY
Matched Family	28537	CP001084	Lactobacillus casei str. Zhang, complete genome.	Lactobacillales	Asp-tRNA-Asn/Glu-tRNA-Gln amidotransferase C subunit	ADK18278	100.0

Input Sequence	ATG-E1_Chromosome_262 # 273396 # 273692 # 1 # ID=1_262;partial=00;start_type=ATG;rbs_motif=GGAG/GAGG;rbs_spacer=5-10bp;gc_cont=0.421						
	PROJECT ID	ACCESSION ID	ORGANISMS	CLASS	PROTEIN FUNCTION	PROTEIN ID	%IDENTITY
Matched Family	28537	CP001084	Lactobacillus casei str. Zhang, complete genome.	Lactobacillales	conserved hypothetical protein	ADK17491	100.0

Input Sequence	ATG-E1_Chromosome_247 # 258478 # 258774 # 1 # ID=1_247;partial=00;start_type=ATG;rbs_motif=AGGAG/GGAGG;rbs_spacer=11-12bp;gc_cont=0.475						
	PROJECT ID	ACCESSION ID	ORGANISMS	CLASS	PROTEIN FUNCTION	PROTEIN ID	%IDENTITY
Matched Family	28537	CP001084	Lactobacillus casei str. Zhang, complete genome.	Lactobacillales	conserved hypothetical protein	ADK17472	100.0

Input Sequence	ATG-E1_Chromosome_2568 # 2611641 # 2611901 # -1 # ID=1_2568;partial=00;start_type=ATG;rbs_motif=GGA/GAG/AGG;rbs_spacer=5-10bp;gc_cont=0.421						
	PROJECT ID	ACCESSION ID	ORGANISMS	CLASS	PROTEIN FUNCTION	PROTEIN ID	%IDENTITY

Matched Family	30359	FM177140	Lactobacillus casei BL23 complete genome, strain BL23.	Lactobacillales	Putative uncharacterized protein	CAQ67621	100.0

Input Sequence: ATG-E1_Chromosome_1199 # 1213833 # 1214126 # 1 # ID=1_1199;partial=00;start_type=ATG;rbs_motif=AGGAG;rbs_spacer=5-10bp;gc_cont=0.446

	PROJECT ID	ACCESSION ID	ORGANISMS	CLASS	PROTEIN FUNCTION	PROTEIN ID	%IDENTITY
Matched Family	30359	FM177140	Lactobacillus casei BL23 complete genome, strain BL23.	Lactobacillales	Prophage Lp3 protein 19, head-to-tail joining	CAQ68146	100.0

Input Sequence: ATG-E1_Chromosome_2133 # 2147087 # 2147380 # -1 # ID=1_2133;partial=00;start_type=GTG;rbs_motif=GGA/GAG/AGG;rbs_spacer=5-10bp;gc_cont=0.435

	PROJECT ID	ACCESSION ID	ORGANISMS	CLASS	PROTEIN FUNCTION	PROTEIN ID	%IDENTITY
Matched Family	402	CP000423	Lactobacillus casei ATCC 334, complete genome.	Lactobacillales	hypothetical protein	ABJ70658	100.0

Input Sequence: ATG-E1_Chromosome_2529 # 2569400 # 2569690 # 1 # ID=1_2529;partial=00;start_type=ATG;rbs_motif=AGGAGG;rbs_spacer=5-10bp;gc_cont=0.512

	PROJECT ID	ACCESSION ID	ORGANISMS	CLASS	PROTEIN FUNCTION	PROTEIN ID	%IDENTITY
Matched Family	30359	FM177140	Lactobacillus casei BL23 complete genome, strain BL23.	Lactobacillales	Acetyltransferase	CAQ67577	100.0

Input Sequence: ATG-E1_Chromosome_138 # 145311 # 145601 # -1 # ID=1_138;partial=00;start_type=ATG;rbs_motif=GGAG/GAGG;rbs_spacer=5-10bp;gc_cont=0.412

	PROJECT ID	ACCESSION ID	ORGANISMS	CLASS	PROTEIN FUNCTION	PROTEIN ID	%IDENTITY
Matched Family	28537	CP001084	Lactobacillus casei str. Zhang, complete genome.	Lactobacillales	conserved hypothetical protein	ADK17352	100.0

Input Sequence: ATG-E1_Chromosome_1562 # 1549857 # 1550147 # 1 # ID=1_1562;partial=00;start_type=ATG;rbs_motif=AGGAG/GGAGG;rbs_spacer=11-12bp;gc_cont=0.405

	PROJECT ID	ACCESSION ID	ORGANISMS	CLASS	PROTEIN FUNCTION	PROTEIN ID	%IDENTITY
Matched Family	28537	CP001084	Lactobacillus casei str. Zhang, complete genome.	Lactobacillales	Predicted pyrophosphatase	ADK18563	100.0

Input Sequence: ATG-E1_Chromosome_1356 # 1346480 # 1346767 # -1 # ID=1_1356;partial=00;start_type=ATG;rbs_motif=GGAGG;rbs_spacer=5-10bp;gc_cont=0.420

	PROJECT ID	ACCESSION ID	ORGANISMS	CLASS	PROTEIN FUNCTION	PROTEIN ID	%IDENTITY
Matched Family	30359	FM177140	Lactobacillus casei BL23 complete genome, strain BL23.	Lactobacillales	Putative uncharacterized protein ycnE	CAQ66433	100.0

Input Sequence: ATG-E1_Chromosome_2627 # 2667719 # 2668006 # -1 # ID=1_2627;partial=00;start_type=TTG;rbs_motif=GGAG/GAGG;rbs_spacer=5-10bp;gc_cont=0.455

	PROJECT ID	ACCESSION ID	ORGANISMS	CLASS	PROTEIN FUNCTION	PROTEIN ID	%IDENTITY
Matched Family	28537	CP001084	Lactobacillus casei str. Zhang, complete genome.	Lactobacillales	conserved hypothetical protein	ADK19616	100.0

Input Sequence: ATG-E1_Chromosome_1440 # 1423474 # 1423731 # 1 # ID=1_1440;partial=00;start_type=ATG;rbs_motif=AGxAG;rbs_spacer=5-10bp;gc_cont=0.422

	PROJECT ID	ACCESSION ID	ORGANISMS	CLASS	PROTEIN FUNCTION	PROTEIN ID	%IDENTITY
Matched Family	30359	FM177140	Lactobacillus casei BL23 complete genome, strain BL23.	Lactobacillales	YceK	CAQ66516	100.0

Input Sequence: ATG-E1_Chromosome_2565 # 2609105 # 2609389 # -1 # ID=1_2565;partial=00;start_type=TTG;rbs_motif=AGGAG;rbs_spacer=5-10bp;gc_cont=0.495

	PROJECT ID	ACCESSION ID	ORGANISMS	CLASS	PROTEIN FUNCTION	PROTEIN ID	%IDENTITY
Matched Family	28537	CP001084	Lactobacillus casei str. Zhang, complete genome.	Lactobacillales	Prebacteriocin	ADK19549	100.0

Input Sequence: ATG-E1_Chromosome_715 # 725642 # 725926 # -1 # ID=1_715;partial=00;start_type=ATG;rbs_motif=GGAG/GAGG;rbs_spacer=5-10bp;gc_cont=0.396

	PROJECT ID	ACCESSION ID	ORGANISMS	CLASS	PROTEIN FUNCTION	PROTEIN ID	%IDENTITY
Matched Family	32197	FM179324	Lactobacillus rhamnosus Lc 705 plasmid sequence, strain	Lactobacillales	Conserved cytosolic protein	CAR91822	100.0

		Lc 705.				

Input Sequence: ATG-E1_Chromosome_2564 # 2608803 # 2609081 # -1 # ID=1_2564;partial=00;start_type=ATG;rbs_motif=AGGAGG;rbs_spacer=5-10bp;gc_cont=0.427

	PROJECT ID	ACCESSION ID	ORGANISMS	CLASS	PROTEIN FUNCTION	PROTEIN ID	%IDENTITY
**Matched Family**	28537	CP001084	Lactobacillus casei str. Zhang, complete genome.	Lactobacillales	Prebacteriocin	ADK19548	100.0

Input Sequence: ATG-E1_Chromosome_1417 # 1401779 # 1402057 # -1 # ID=1_1417;partial=00;start_type=GTG;rbs_motif=AGGAGG;rbs_spacer=5-10bp;gc_cont=0.423

	PROJECT ID	ACCESSION ID	ORGANISMS	CLASS	PROTEIN FUNCTION	PROTEIN ID	%IDENTITY
**Matched Family**	402	CP000423	Lactobacillus casei ATCC 334, complete genome.	Lactobacillales	hypothetical protein	ABJ69979	100.0

Input Sequence: ATG-E1_Chromosome_546 # 558972 # 559241 # -1 # ID=1_546;partial=00;start_type=ATG;rbs_motif=GGA/GAG/AGG;rbs_spacer=5-10bp;gc_cont=0.496

	PROJECT ID	ACCESSION ID	ORGANISMS	CLASS	PROTEIN FUNCTION	PROTEIN ID	%IDENTITY
**Matched Family**	28537	CP001084	Lactobacillus casei str. Zhang, complete genome.	Lactobacillales	conserved hypothetical protein	ADK17724	100.0

Input Sequence: ATG-E1_Chromosome_1084 # 1100191 # 1100460 # 1 # ID=1_1084;partial=00;start_type=ATG;rbs_motif=GGAG/GAGG;rbs_spacer=5-10bp;gc_cont=0.489

	PROJECT ID	ACCESSION ID	ORGANISMS	CLASS	PROTEIN FUNCTION	PROTEIN ID	%IDENTITY
**Matched Family**	30359	FM177140	Lactobacillus casei BL23 complete genome, strain BL23.	Lactobacillales	Putative uncharacterized protein	CAQ66143	100.0

Input Sequence: ATG-E1_Chromosome_1036 # 1051947 # 1052213 # 1 # ID=1_1036;partial=00;start_type=ATG;rbs_motif=AGGA/GGAG/GAGG;rbs_spacer=11-12bp;gc_cont=0.401

	PROJECT ID	ACCESSION ID	ORGANISMS	CLASS	PROTEIN FUNCTION	PROTEIN ID	%IDENTITY
**Matched Family**	28537	CP001084	Lactobacillus casei str. Zhang, complete genome.	Lactobacillales	ACT domain-containing protein	ADK18086	100.0

Input Sequence: ATG-E1_Chromosome_2452 # 2489674 # 2489937 # -1 # ID=1_2452;partial=00;start_type=ATG;rbs_motif=AGGA;rbs_spacer=5-10bp;gc_cont=0.508

	PROJECT ID	ACCESSION ID	ORGANISMS	CLASS	PROTEIN FUNCTION	PROTEIN ID	%IDENTITY
**Matched Family**	30359	FM177140	Lactobacillus casei BL23 complete genome, strain BL23.	Lactobacillales	Putative uncharacterized protein	CAQ67508	100.0

Input Sequence: ATG-E1_Chromosome_2853 # 2897838 # 2898101 # 1 # ID=1_2853;partial=00;start_type=ATG;rbs_motif=GGAG/GAGG;rbs_spacer=5-10bp;gc_cont=0.455

	PROJECT ID	ACCESSION ID	ORGANISMS	CLASS	PROTEIN FUNCTION	PROTEIN ID	%IDENTITY
**Matched Family**	28537	CP001084	Lactobacillus casei str. Zhang, complete genome.	Lactobacillales	conserved hypothetical protein	ADK19799	100.0

Input Sequence: ATG-E1_Chromosome_1993 # 2005866 # 2006126 # -1 # ID=1_1993;partial=00;start_type=ATG;rbs_motif=AGGAG;rbs_spacer=5-10bp;gc_cont=0.444

	PROJECT ID	ACCESSION ID	ORGANISMS	CLASS	PROTEIN FUNCTION	PROTEIN ID	%IDENTITY
**Matched Family**	28537	CP001084	Lactobacillus casei str. Zhang, complete genome.	Lactobacillales	Phosphoribosylformylglycinamidine (FGAM) synthase, PurS component	ADK18970	100.0

Input Sequence: ATG-E1_Chromosome_61 # 67285 # 67545 # 1 # ID=1_61;partial=00;start_type=ATG;rbs_motif=GGAGG;rbs_spacer=5-10bp;gc_cont=0.402

	PROJECT ID	ACCESSION ID	ORGANISMS	CLASS	PROTEIN FUNCTION	PROTEIN ID	%IDENTITY
**Matched Family**	28537	CP001084	Lactobacillus casei str. Zhang, complete genome.	Lactobacillales	conserved hypothetical protein	ADK20082	100.0

Input Sequence: ATG-E1_Chromosome_611 # 646957 # 647217 # -1 # ID=1_611;partial=00;start_type=ATG;rbs_motif=AGxAGG/AGGxGG;rbs_spacer=5-10bp;gc_cont=0.360

	PROJECT ID	ACCESSION ID	ORGANISMS	CLASS	PROTEIN FUNCTION	PROTEIN ID	%IDENTITY
**Matched Family**	30359	FM177140	Lactobacillus casei BL23 complete genome, strain BL23.	Lactobacillales	Putative uncharacterized protein	CAQ65674	100.0

**Input Sequence**	ATG-E1_Chromosome_1251 # 1264875 # 1265132 # -1 # ID=1_1251;partial=00;start_type=ATG;rbs_motif=GGAGG;rbs_spacer=5-10bp;gc_cont=0.469						
	PROJECT ID	ACCESSION ID	ORGANISMS	CLASS	PROTEIN FUNCTION	PROTEIN ID	%IDENTITY
**Matched Family**	28537	CP001084	Lactobacillus casei str. Zhang, complete genome.	Lactobacillales	Small conserved protein	ADK18312	100.0

**Input Sequence**	ATG-E1_Chromosome_2098 # 2116478 # 2116735 # -1 # ID=1_2098;partial=00;start_type=ATG;rbs_motif=AGGAGG;rbs_spacer=5-10bp;gc_cont=0.457						
	PROJECT ID	ACCESSION ID	ORGANISMS	CLASS	PROTEIN FUNCTION	PROTEIN ID	%IDENTITY
**Matched Family**	28537	CP001084	Lactobacillus casei str. Zhang, complete genome.	Lactobacillales	conserved hypothetical protein	ADK19078	100.0

**Input Sequence**	ATG-E1_Chromosome_2544 # 2587511 # 2587768 # -1 # ID=1_2544;partial=00;start_type=GTG;rbs_motif=AGGAGG;rbs_spacer=5-10bp;gc_cont=0.453						
	PROJECT ID	ACCESSION ID	ORGANISMS	CLASS	PROTEIN FUNCTION	PROTEIN ID	%IDENTITY
**Matched Family**	28537	CP001084	Lactobacillus casei str. Zhang, complete genome.	Lactobacillales	conserved hypothetical protein	ADK19525	100.0

**Input Sequence**	ATG-E1_Chromosome_14 # 12656 # 12910 # -1 # ID=1_14;partial=00;start_type=ATG;rbs_motif=AGxAGG/AGGxGG;rbs_spacer=5-10bp;gc_cont=0.471						
	PROJECT ID	ACCESSION ID	ORGANISMS	CLASS	PROTEIN FUNCTION	PROTEIN ID	%IDENTITY
**Matched Family**	28537	CP001084	Lactobacillus casei str. Zhang, complete genome.	Lactobacillales	hypothetical protein	ADK20037	100.0

**Input Sequence**	ATG-E1_Chromosome_1837 # 1853413 # 1853667 # -1 # ID=1_1837;partial=00;start_type=ATG;rbs_motif=AGxAGG/AGGxGG;rbs_spacer=5-10bp;gc_cont=0.510						
	PROJECT ID	ACCESSION ID	ORGANISMS	CLASS	PROTEIN FUNCTION	PROTEIN ID	%IDENTITY
**Matched Family**	28537	CP001084	Lactobacillus casei str. Zhang, complete genome.	Lactobacillales	RNA-binding protein, KH domain	ADK18819	100.0

**Input Sequence**	ATG-E1_Chromosome_668 # 686257 # 686511 # 1 # ID=1_668;partial=00;start_type=ATG;rbs_motif=AGGAGG;rbs_spacer=5-10bp;gc_cont=0.431						
	PROJECT ID	ACCESSION ID	ORGANISMS	CLASS	PROTEIN FUNCTION	PROTEIN ID	%IDENTITY
**Matched Family**	402	CP000423	Lactobacillus casei ATCC 334, complete genome.	Lactobacillales	hypothetical protein	ABJ69393	100.0

**Input Sequence**	ATG-E1_Chromosome_224 # 237874 # 238125 # 1 # ID=1_224;partial=00;start_type=ATG;rbs_motif=GGA/GAG/AGG;rbs_spacer=5-10bp;gc_cont=0.556						
	PROJECT ID	ACCESSION ID	ORGANISMS	CLASS	PROTEIN FUNCTION	PROTEIN ID	%IDENTITY
**Matched Family**	30359	FM177140	Lactobacillus casei BL23 complete genome, strain BL23.	Lactobacillales	Putative uncharacterized protein	CAQ65266	100.0

**Input Sequence**	ATG-E1_Chromosome_624 # 654614 # 654865 # -1 # ID=1_624;partial=00;start_type=ATG;rbs_motif=AGGAGG;rbs_spacer=5-10bp;gc_cont=0.492						
	PROJECT ID	ACCESSION ID	ORGANISMS	CLASS	PROTEIN FUNCTION	PROTEIN ID	%IDENTITY
**Matched Family**	30359	FM177140	Lactobacillus casei BL23 complete genome, strain BL23.	Lactobacillales	Putative uncharacterized protein	CAQ65685	100.0

**Input Sequence**	ATG-E1_Chromosome_1868 # 1886175 # 1886423 # -1 # ID=1_1868;partial=00;start_type=ATG;rbs_motif=AGGAG;rbs_spacer=5-10bp;gc_cont=0.410						
	PROJECT ID	ACCESSION ID	ORGANISMS	CLASS	PROTEIN FUNCTION	PROTEIN ID	%IDENTITY
**Matched Family**	28537	CP001084	Lactobacillus casei str. Zhang, complete genome.	Lactobacillales	DNA-directed RNA polymerase, subunit K/omega	ADK18847	100.0

**Input Sequence**	ATG-E1_Chromosome_1762 # 1764444 # 1764692 # 1 # ID=1_1762;partial=00;start_type=ATG;rbs_motif=AGGA;rbs_spacer=5-10bp;gc_cont=0.482						
	PROJECT ID	ACCESSION ID	ORGANISMS	CLASS	PROTEIN FUNCTION	PROTEIN ID	%IDENTITY
**Matched Family**	28537	CP001084	Lactobacillus casei str. Zhang, complete genome.	Lactobacillales	conserved hypothetical protein	ADK18760	100.0

Input

Sequence ATG-E1_Chromosome_2772 # 2814844 # 2815092 # -1 # ID=1_2772;partial=00;start_type=ATG;rbs_motif=GGAGG;rbs_spacer=5-10bp;gc_cont=0.474

	PROJECT ID	ACCESSION ID	ORGANISMS	CLASS	PROTEIN FUNCTION	PROTEIN ID	%IDENTITY
**Matched Family**	28537	CP001084	Lactobacillus casei str. Zhang, complete genome.	Lactobacillales	conserved hypothetical protein	ADK19713	100.0

Input Sequence ATG-E1_Chromosome_619 # 651273 # 651521 # 1 # ID=1_619;partial=00;start_type=ATG;rbs_motif=GGAGG;rbs_spacer=5-10bp;gc_cont=0.402

	PROJECT ID	ACCESSION ID	ORGANISMS	CLASS	PROTEIN FUNCTION	PROTEIN ID	%IDENTITY
**Matched Family**	30359	FM177140	Lactobacillus casei BL23 complete genome, strain BL23.	Lactobacillales	Putative uncharacterized protein	CAQ65681	100.0

Input Sequence ATG-E1_Chromosome_953 # 966462 # 966707 # 1 # ID=1_953;partial=00;start_type=ATG;rbs_motif=GGAGG;rbs_spacer=5-10bp;gc_cont=0.472

	PROJECT ID	ACCESSION ID	ORGANISMS	CLASS	PROTEIN FUNCTION	PROTEIN ID	%IDENTITY
**Matched Family**	30359	FM177140	Lactobacillus casei BL23 complete genome, strain BL23.	Lactobacillales	Stimulator of FtsZ polymerization and component of cell-division Z-ring	CAQ65973	100.0

Input Sequence ATG-E1_Chromosome_1832 # 1850823 # 1851068 # -1 # ID=1_1832;partial=00;start_type=ATG;rbs_motif=AGGAGG;rbs_spacer=5-10bp;gc_cont=0.431

	PROJECT ID	ACCESSION ID	ORGANISMS	CLASS	PROTEIN FUNCTION	PROTEIN ID	%IDENTITY
**Matched Family**	28537	CP001084	Lactobacillus casei str. Zhang, complete genome.	Lactobacillales	conserved hypothetical protein	ADK18814	100.0

Input Sequence ATG-E1_Chromosome_1850 # 1868464 # 1868706 # -1 # ID=1_1850;partial=00;start_type=ATG;rbs_motif=GGAGG;rbs_spacer=5-10bp;gc_cont=0.412

	PROJECT ID	ACCESSION ID	ORGANISMS	CLASS	PROTEIN FUNCTION	PROTEIN ID	%IDENTITY
**Matched Family**	28537	CP001084	Lactobacillus casei str. Zhang, complete genome.	Lactobacillales	Acyl carrier protein	ADK18831	100.0

Input Sequence ATG-E1_Chromosome_2397 # 2439399 # 2439641 # 1 # ID=1_2397;partial=00;start_type=ATG;rbs_motif=AGxAGG/AGGxGG;rbs_spacer=5-10bp;gc_cont=0.457

	PROJECT ID	ACCESSION ID	ORGANISMS	CLASS	PROTEIN FUNCTION	PROTEIN ID	%IDENTITY
**Matched Family**	28537	CP001084	Lactobacillus casei str. Zhang, complete genome.	Lactobacillales	conserved hypothetical protein	ADK19384	100.0

Input Sequence ATG-E1_Chromosome_288 # 301764 # 302006 # 1 # ID=1_288;partial=00;start_type=ATG;rbs_motif=GGAG/GAGG;rbs_spacer=5-10bp;gc_cont=0.457

	PROJECT ID	ACCESSION ID	ORGANISMS	CLASS	PROTEIN FUNCTION	PROTEIN ID	%IDENTITY
**Matched Family**	30359	FM177140	Lactobacillus casei BL23 complete genome, strain BL23.	Lactobacillales	Putative uncharacterized protein	CAQ65316	100.0

Input Sequence ATG-E1_Chromosome_1391 # 1382329 # 1382565 # 1 # ID=1_1391;partial=00;start_type=ATG;rbs_motif=GGAG/GAGG;rbs_spacer=5-10bp;gc_cont=0.430

	PROJECT ID	ACCESSION ID	ORGANISMS	CLASS	PROTEIN FUNCTION	PROTEIN ID	%IDENTITY
**Matched Family**	28537	CP001084	Lactobacillus casei str. Zhang, complete genome.	Lactobacillales	Predicted membrane protein	ADK18396	100.0

Input Sequence ATG-E1_Chromosome_613 # 647911 # 648147 # -1 # ID=1_613;partial=00;start_type=GTG;rbs_motif=AGxAG;rbs_spacer=5-10bp;gc_cont=0.414

	PROJECT ID	ACCESSION ID	ORGANISMS	CLASS	PROTEIN FUNCTION	PROTEIN ID	%IDENTITY
**Matched Family**	402	CP000423	Lactobacillus casei ATCC 334, complete genome.	Lactobacillales	hypothetical protein	ABJ69339	100.0

Input Sequence ATG-E1_Chromosome_2590 # 2628930 # 2629163 # -1 # ID=1_2590;partial=00;start_type=ATG;rbs_motif=AGGA;rbs_spacer=5-10bp;gc_cont=0.466

	PROJECT ID	ACCESSION ID	ORGANISMS	CLASS	PROTEIN FUNCTION	PROTEIN ID	%IDENTITY
**Matched Family**	30359	FM177140	Lactobacillus casei BL23 complete genome, strain BL23.	Lactobacillales	Putative uncharacterized protein	CAQ67642	100.0

Input Sequence ATG-E1_Chromosome_155 # 164193 # 164420 # 1 # ID=1_155;partial=00;start_type=ATG;rbs_motif=AGGA;rbs_spacer=5-10bp;gc_cont=0.439

PROJECT	ACCESSION ID	ORGANISMS	CLASS	PROTEIN FUNCTION	PROTEIN ID	%IDENTITY

	ID						
**Matched Family**	28537	CP001084	Lactobacillus casei str. Zhang, complete genome.	Lactobacillales	Copper chaperone	ADK17378	100.0

**Input Sequence**	ATG-E1_Chromosome_1698 # 1699507 # 1699734 # -1 # ID=1_1698;partial=00;start_type=ATG;rbs_motif=AGGAGG;rbs_spacer=5-10bp;gc_cont=0.491

	PROJECT ID	ACCESSION ID	ORGANISMS	CLASS	PROTEIN FUNCTION	PROTEIN ID	%IDENTITY
**Matched Family**	28537	CP001084	Lactobacillus casei str. Zhang, complete genome.	Lactobacillales	conserved hypothetical protein	ADK18697	100.0

**Input Sequence**	ATG-E1_Chromosome_651 # 671171 # 671398 # 1 # ID=1_651;partial=00;start_type=ATG;rbs_motif=AGxAGG/AGGxGG;rbs_spacer=5-10bp;gc_cont=0.456

	PROJECT ID	ACCESSION ID	ORGANISMS	CLASS	PROTEIN FUNCTION	PROTEIN ID	%IDENTITY
**Matched Family**	30359	FM177140	Lactobacillus casei BL23 complete genome, strain BL23.	Lactobacillales	Putative uncharacterized protein	CAQ65711	100.0

**Input Sequence**	ATG-E1_Chromosome_2580 # 2622410 # 2622634 # 1 # ID=1_2580;partial=00;start_type=ATG;rbs_motif=GGAG/GAGG;rbs_spacer=5-10bp;gc_cont=0.387

	PROJECT ID	ACCESSION ID	ORGANISMS	CLASS	PROTEIN FUNCTION	PROTEIN ID	%IDENTITY
**Matched Family**	402	CP000423	Lactobacillus casei ATCC 334, complete genome.	Lactobacillales	hypothetical protein	ABJ71133	100.0

**Input Sequence**	ATG-E1_Chromosome_2689 # 2724685 # 2724906 # 1 # ID=1_2689;partial=00;start_type=ATG;rbs_motif=GGAGG;rbs_spacer=5-10bp;gc_cont=0.446

	PROJECT ID	ACCESSION ID	ORGANISMS	CLASS	PROTEIN FUNCTION	PROTEIN ID	%IDENTITY
**Matched Family**	28537	CP001084	Lactobacillus casei str. Zhang, complete genome.	Lactobacillales	conserved hypothetical protein	ADK19642	100.0

**Input Sequence**	ATG-E1_Chromosome_153 # 161750 # 161971 # -1 # ID=1_153;partial=00;start_type=ATG;rbs_motif=AGGA/GGAG/GAGG;rbs_spacer=11-12bp;gc_cont=0.410

	PROJECT ID	ACCESSION ID	ORGANISMS	CLASS	PROTEIN FUNCTION	PROTEIN ID	%IDENTITY
**Matched Family**	402	CP000423	Lactobacillus casei ATCC 334, complete genome.	Lactobacillales	hypothetical protein	ABJ68932	100.0

**Input Sequence**	ATG-E1_Chromosome_1395 # 1384107 # 1384328 # 1 # ID=1_1395;partial=00;start_type=ATG;rbs_motif=AGGAGG;rbs_spacer=5-10bp;gc_cont=0.455

	PROJECT ID	ACCESSION ID	ORGANISMS	CLASS	PROTEIN FUNCTION	PROTEIN ID	%IDENTITY
**Matched Family**	28537	CP001084	Lactobacillus casei str. Zhang, complete genome.	Lactobacillales	conserved hypothetical protein	ADK18400	100.0

**Input Sequence**	ATG-E1_Chromosome_640 # 664657 # 664875 # -1 # ID=1_640;partial=00;start_type=ATG;rbs_motif=AGGAGG;rbs_spacer=5-10bp;gc_cont=0.416

	PROJECT ID	ACCESSION ID	ORGANISMS	CLASS	PROTEIN FUNCTION	PROTEIN ID	%IDENTITY
**Matched Family**	30359	FM177140	Lactobacillus casei BL23 complete genome, strain BL23.	Lactobacillales	Putative uncharacterized protein	CAQ65700	100.0

**Input Sequence**	ATG-E1_Chromosome_3010 # 3057141 # 3057359 # -1 # ID=1_3010;partial=00;start_type=ATG;rbs_motif=AGxAGG/AGGxGG;rbs_spacer=5-10bp;gc_cont=0.452

	PROJECT ID	ACCESSION ID	ORGANISMS	CLASS	PROTEIN FUNCTION	PROTEIN ID	%IDENTITY
**Matched Family**	28537	CP001084	Lactobacillus casei str. Zhang, complete genome.	Lactobacillales	conserved hypothetical protein	ADK19955	100.0

**Input Sequence**	ATG-E1_Chromosome_429 # 440383 # 440598 # 1 # ID=1_429;partial=00;start_type=ATG;rbs_motif=GGAG/GAGG;rbs_spacer=5-10bp;gc_cont=0.481

	PROJECT ID	ACCESSION ID	ORGANISMS	CLASS	PROTEIN FUNCTION	PROTEIN ID	%IDENTITY
**Matched Family**	30359	FM177140	Lactobacillus casei BL23 complete genome, strain BL23.	Lactobacillales	Putative uncharacterized protein	CAQ65458	100.0

**Input Sequence**	ATG-E1_Chromosome_2589 # 2628718 # 2628933 # -1 # ID=1_2589;partial=00;start_type=ATG;rbs_motif=AGGAG;rbs_spacer=5-10bp;gc_cont=0.449

	PROJECT ID	ACCESSION ID	ORGANISMS	CLASS	PROTEIN FUNCTION	PROTEIN ID	%IDENTITY
**Matched**	30359	FM177140	Lactobacillus casei BL23	Lactobacillales	Putative uncharacterized protein	CAQ67641	100.0

**Family**			complete genome, strain BL23.				

**Input Sequence**	ATG-E1_Chromosome_1207 # 1219174 # 1219383 # -1 # ID=1_1207;partial=00;start_type=ATG;rbs_motif=AGxAGG/AGGxGG;rbs_spacer=5-10bp;gc_cont=0.448						
	PROJECT ID	ACCESSION ID	ORGANISMS	CLASS	PROTEIN FUNCTION	PROTEIN ID	%IDENTITY
**Matched Family**	28537	CP001084	Lactobacillus casei str. Zhang, complete genome.	Lactobacillales	conserved hypothetical protein	ADK18266	100.0

**Input Sequence**	ATG-E1_Chromosome_1252 # 1265148 # 1265357 # -1 # ID=1_1252;partial=00;start_type=ATG;rbs_motif=AGGAGG;rbs_spacer=5-10bp;gc_cont=0.510						
	PROJECT ID	ACCESSION ID	ORGANISMS	CLASS	PROTEIN FUNCTION	PROTEIN ID	%IDENTITY
**Matched Family**	28537	CP001084	Lactobacillus casei str. Zhang, complete genome.	Lactobacillales	conserved hypothetical protein	ADK18313	100.0

**Input Sequence**	ATG-E1_Chromosome_78 # 88694 # 88903 # -1 # ID=1_78;partial=00;start_type=ATG;rbs_motif=AGGAG;rbs_spacer=5-10bp;gc_cont=0.357						
	PROJECT ID	ACCESSION ID	ORGANISMS	CLASS	PROTEIN FUNCTION	PROTEIN ID	%IDENTITY
**Matched Family**	28537	CP001084	Lactobacillus casei str. Zhang, complete genome.	Lactobacillales	hypothetical protein	ADK20094	100.0

**Input Sequence**	ATG-E1_Chromosome_284 # 299658 # 299864 # -1 # ID=1_284;partial=00;start_type=TTG;rbs_motif=AGGAGG;rbs_spacer=5-10bp;gc_cont=0.430						
	PROJECT ID	ACCESSION ID	ORGANISMS	CLASS	PROTEIN FUNCTION	PROTEIN ID	%IDENTITY
**Matched Family**	28537	CP001084	Lactobacillus casei str. Zhang, complete genome.	Lactobacillales	conserved hypothetical protein	ADK17511	100.0

**Input Sequence**	ATG-E1_Chromosome_2326 # 2356609 # 2356815 # 1 # ID=1_2326;partial=00;start_type=GTG;rbs_motif=GGAGG;rbs_spacer=5-10bp;gc_cont=0.449						
	PROJECT ID	ACCESSION ID	ORGANISMS	CLASS	PROTEIN FUNCTION	PROTEIN ID	%IDENTITY
**Matched Family**	402	CP000423	Lactobacillus casei ATCC 334, complete genome.	Lactobacillales	hypothetical protein	ABJ70885	100.0

**Input Sequence**	ATG-E1_Chromosome_2545 # 2587839 # 2588045 # -1 # ID=1_2545;partial=00;start_type=ATG;rbs_motif=AGxAGG/AGGxGG;rbs_spacer=11-12bp;gc_cont=0.478						
	PROJECT ID	ACCESSION ID	ORGANISMS	CLASS	PROTEIN FUNCTION	PROTEIN ID	%IDENTITY
**Matched Family**	28537	CP001084	Lactobacillus casei str. Zhang, complete genome.	Lactobacillales	conserved hypothetical protein	ADK19526	100.0

**Input Sequence**	ATG-E1_Chromosome_627 # 655372 # 655575 # 1 # ID=1_627;partial=00;start_type=TTG;rbs_motif=GGAGG;rbs_spacer=5-10bp;gc_cont=0.373						
	PROJECT ID	ACCESSION ID	ORGANISMS	CLASS	PROTEIN FUNCTION	PROTEIN ID	%IDENTITY
**Matched Family**	30359	FM177140	Lactobacillus casei BL23 complete genome, strain BL23.	Lactobacillales	Putative uncharacterized protein	CAQ65687	100.0

**Input Sequence**	ATG-E1_Chromosome_294 # 305328 # 305525 # 1 # ID=1_294;partial=00;start_type=ATG;rbs_motif=AGGAG;rbs_spacer=5-10bp;gc_cont=0.525						
	PROJECT ID	ACCESSION ID	ORGANISMS	CLASS	PROTEIN FUNCTION	PROTEIN ID	%IDENTITY
**Matched Family**	28537	CP001084	Lactobacillus casei str. Zhang, complete genome.	Lactobacillales	conserved hypothetical protein	ADK17521	100.0

**Input Sequence**	ATG-E1_Chromosome_2779 # 2820913 # 2821110 # -1 # ID=1_2779;partial=00;start_type=ATG;rbs_motif=AGGA/GGAG/GAGG;rbs_spacer=11-12bp;gc_cont=0.561						
	PROJECT ID	ACCESSION ID	ORGANISMS	CLASS	PROTEIN FUNCTION	PROTEIN ID	%IDENTITY
**Matched Family**	28537	CP001084	Lactobacillus casei str. Zhang, complete genome.	Lactobacillales	hypothetical protein	ADK19720	100.0

**Input Sequence**	ATG-E1_Chromosome_289 # 302003 # 302197 # 1 # ID=1_289;partial=00;start_type=ATG;rbs_motif=None;rbs_spacer=None;gc_cont=0.472						
	PROJECT ID	ACCESSION ID	ORGANISMS	CLASS	PROTEIN FUNCTION	PROTEIN ID	%IDENTITY
**Matched Family**	28537	CP001084	Lactobacillus casei str. Zhang, complete genome.	Lactobacillales	conserved hypothetical protein	ADK17516	100.0

**Input Sequence**	ATG-E1_Chromosome_899 # 912740 # 912934 # 1 # ID=1_899;partial=00;start_type=ATG;rbs_motif=AGGAG;rbs_spacer=5-10bp;gc_cont=0.431

	PROJECT ID	ACCESSION ID	ORGANISMS	CLASS	PROTEIN FUNCTION	PROTEIN ID	%IDENTITY
**Matched Family**	28537	CP001084	Lactobacillus casei str. Zhang, complete genome.	Lactobacillales	conserved hypothetical protein	ADK17955	100.0

**Input Sequence**	ATG-E1_Chromosome_1233 # 1247375 # 1247569 # -1 # ID=1_1233;partial=00;start_type=ATG;rbs_motif=AGGAG;rbs_spacer=5-10bp;gc_cont=0.467

	PROJECT ID	ACCESSION ID	ORGANISMS	CLASS	PROTEIN FUNCTION	PROTEIN ID	%IDENTITY
**Matched Family**	402	CP000423	Lactobacillus casei ATCC 334, complete genome.	Lactobacillales	hypothetical protein	ABJ69865	100.0

**Input Sequence**	ATG-E1_Chromosome_2550 # 2592655 # 2592849 # -1 # ID=1_2550;partial=00;start_type=ATG;rbs_motif=GGAGG;rbs_spacer=5-10bp;gc_cont=0.431

	PROJECT ID	ACCESSION ID	ORGANISMS	CLASS	PROTEIN FUNCTION	PROTEIN ID	%IDENTITY
**Matched Family**	28537	CP001084	Lactobacillus casei str. Zhang, complete genome.	Lactobacillales	conserved hypothetical protein	ADK19532	100.0

**Input Sequence**	ATG-E1_Chromosome_2603 # 2643936 # 2644127 # 1 # ID=1_2603;partial=00;start_type=ATG;rbs_motif=AGGAGG;rbs_spacer=5-10bp;gc_cont=0.490

	PROJECT ID	ACCESSION ID	ORGANISMS	CLASS	PROTEIN FUNCTION	PROTEIN ID	%IDENTITY
**Matched Family**	30359	FM177140	Lactobacillus casei BL23 complete genome, strain BL23.	Lactobacillales	Putative uncharacterized protein	CAQ67654	100.0

**Input Sequence**	ATG-E1_Chromosome_373 # 384167 # 384355 # -1 # ID=1_373;partial=00;start_type=ATG;rbs_motif=AGGAG;rbs_spacer=5-10bp;gc_cont=0.434

	PROJECT ID	ACCESSION ID	ORGANISMS	CLASS	PROTEIN FUNCTION	PROTEIN ID	%IDENTITY
**Matched Family**	28537	CP001084	Lactobacillus casei str. Zhang, complete genome.	Lactobacillales	conserved hypothetical protein	ADK17596	100.0

**Input Sequence**	ATG-E1_Chromosome_2579 # 2621900 # 2622088 # -1 # ID=1_2579;partial=00;start_type=ATG;rbs_motif=GGAGG;rbs_spacer=5-10bp;gc_cont=0.444

	PROJECT ID	ACCESSION ID	ORGANISMS	CLASS	PROTEIN FUNCTION	PROTEIN ID	%IDENTITY
**Matched Family**	402	CP000423	Lactobacillus casei ATCC 334, complete genome.	Lactobacillales	hypothetical protein	ABJ71132	100.0

**Input Sequence**	ATG-E1_Chromosome_106 # 115399 # 115581 # -1 # ID=1_106;partial=00;start_type=ATG;rbs_motif=GGAGG;rbs_spacer=5-10bp;gc_cont=0.459

	PROJECT ID	ACCESSION ID	ORGANISMS	CLASS	PROTEIN FUNCTION	PROTEIN ID	%IDENTITY
**Matched Family**	28537	CP001084	Lactobacillus casei str. Zhang, complete genome.	Lactobacillales	hypothetical protein	ADK17320	100.0

**Input Sequence**	ATG-E1_Chromosome_1411 # 1397454 # 1397636 # 1 # ID=1_1411;partial=00;start_type=ATG;rbs_motif=AGGAGG;rbs_spacer=5-10bp;gc_cont=0.415

	PROJECT ID	ACCESSION ID	ORGANISMS	CLASS	PROTEIN FUNCTION	PROTEIN ID	%IDENTITY
**Matched Family**	28537	CP001084	Lactobacillus casei str. Zhang, complete genome.	Lactobacillales	conserved hypothetical protein	ADK18414	100.0

**Input Sequence**	ATG-E1_Chromosome_1970 # 1980428 # 1980610 # 1 # ID=1_1970;partial=00;start_type=ATG;rbs_motif=GGA/GAG/AGG;rbs_spacer=5-10bp;gc_cont=0.486

	PROJECT ID	ACCESSION ID	ORGANISMS	CLASS	PROTEIN FUNCTION	PROTEIN ID	%IDENTITY
**Matched Family**	402	CP000423	Lactobacillus casei ATCC 334, complete genome.	Lactobacillales	hypothetical protein	ABJ70503	100.0

**Input Sequence**	ATG-E1_Chromosome_842 # 861064 # 861243 # 1 # ID=1_842;partial=00;start_type=ATG;rbs_motif=GGA/GAG/AGG;rbs_spacer=5-10bp;gc_cont=0.467

	PROJECT ID	ACCESSION ID	ORGANISMS	CLASS	PROTEIN FUNCTION	PROTEIN ID	%IDENTITY
**Matched Family**	28537	CP001084	Lactobacillus casei str. Zhang, complete genome.	Lactobacillales	conserved hypothetical protein	ADK17914	100.0

**Input Sequence**	ATG-E1_Chromosome_1212 # 1222630 # 1222806 # -1 # ID=1_1212;partial=00;start_type=ATG;rbs_motif=AGGAGG;rbs_spacer=5-10bp;gc_cont=0.486

	PROJECT ID	ACCESSION ID	ORGANISMS	CLASS	PROTEIN FUNCTION	PROTEIN ID	%IDENTITY
**Matched Family**	28537	CP001084	Lactobacillus casei str. Zhang, complete genome.	Lactobacillales	conserved hypothetical protein	ADK18271	100.0

**Input Sequence**	ATG-E1_Chromosome_1833 # 1851101 # 1851277 # -1 # ID=1_1833;partial=00;start_type=ATG;rbs_motif=GGAG/GAGG;rbs_spacer=5-10bp;gc_cont=0.492						
	PROJECT ID	ACCESSION ID	ORGANISMS	CLASS	PROTEIN FUNCTION	PROTEIN ID	%IDENTITY
**Matched Family**	28537	CP001084	Lactobacillus casei str. Zhang, complete genome.	Lactobacillales	conserved hypothetical protein	ADK18815	100.0

**Input Sequence**	ATG-E1_Chromosome_62 # 67556 # 67732 # 1 # ID=1_62;partial=00;start_type=ATG;rbs_motif=GGAG/GAGG;rbs_spacer=5-10bp;gc_cont=0.452						
	PROJECT ID	ACCESSION ID	ORGANISMS	CLASS	PROTEIN FUNCTION	PROTEIN ID	%IDENTITY
**Matched Family**	30359	FM177140	Lactobacillus casei BL23 complete genome, strain BL23.	Lactobacillales	Putative uncharacterized protein	CAQ68159	100.0

**Input Sequence**	ATG-E1_Chromosome_650 # 670993 # 671169 # 1 # ID=1_650;partial=00;start_type=ATG;rbs_motif=AGGAGG;rbs_spacer=5-10bp;gc_cont=0.362						
	PROJECT ID	ACCESSION ID	ORGANISMS	CLASS	PROTEIN FUNCTION	PROTEIN ID	%IDENTITY
**Matched Family**	30359	FM177140	Lactobacillus casei BL23 complete genome, strain BL23.	Lactobacillales	Putative uncharacterized protein	CAQ65710	100.0

**Input Sequence**	ATG-E1_Chromosome_2484 # 2527434 # 2527604 # -1 # ID=1_2484;partial=00;start_type=ATG;rbs_motif=AGGAG;rbs_spacer=5-10bp;gc_cont=0.433						
	PROJECT ID	ACCESSION ID	ORGANISMS	CLASS	PROTEIN FUNCTION	PROTEIN ID	%IDENTITY
**Matched Family**	28537	CP001084	Lactobacillus casei str. Zhang, complete genome.	Lactobacillales	Preprotein translocase subunit SecE	ADK19471	100.0

**Input Sequence**	ATG-E1_Chromosome_1393 # 1383629 # 1383799 # 1 # ID=1_1393;partial=00;start_type=ATG;rbs_motif=AGGAG;rbs_spacer=5-10bp;gc_cont=0.409						
	PROJECT ID	ACCESSION ID	ORGANISMS	CLASS	PROTEIN FUNCTION	PROTEIN ID	%IDENTITY
**Matched Family**	28537	CP001084	Lactobacillus casei str. Zhang, complete genome.	Lactobacillales	conserved hypothetical protein	ADK18398	100.0

**Input Sequence**	ATG-E1_Chromosome_2581 # 2622666 # 2622836 # -1 # ID=1_2581;partial=00;start_type=ATG;rbs_motif=GGA/GAG/AGG;rbs_spacer=5-10bp;gc_cont=0.322						
	PROJECT ID	ACCESSION ID	ORGANISMS	CLASS	PROTEIN FUNCTION	PROTEIN ID	%IDENTITY
**Matched Family**	28537	CP001084	Lactobacillus casei str. Zhang, complete genome.	Lactobacillales	conserved hypothetical protein	ADK19568	100.0

**Input Sequence**	ATG-E1_Chromosome_2058 # 2074822 # 2074989 # -1 # ID=1_2058;partial=00;start_type=ATG;rbs_motif=GGAG/GAGG;rbs_spacer=5-10bp;gc_cont=0.429						
	PROJECT ID	ACCESSION ID	ORGANISMS	CLASS	PROTEIN FUNCTION	PROTEIN ID	%IDENTITY
**Matched Family**	28537	CP001084	Lactobacillus casei str. Zhang, complete genome.	Lactobacillales	conserved hypothetical protein	ADK19038	100.0

**Input Sequence**	ATG-E1_Chromosome_372 # 383800 # 383964 # -1 # ID=1_372;partial=00;start_type=ATG;rbs_motif=GGAGG;rbs_spacer=5-10bp;gc_cont=0.418						
	PROJECT ID	ACCESSION ID	ORGANISMS	CLASS	PROTEIN FUNCTION	PROTEIN ID	%IDENTITY
**Matched Family**	28537	CP001084	Lactobacillus casei str. Zhang, complete genome.	Lactobacillales	conserved hypothetical protein	ADK17594	100.0

**Input Sequence**	ATG-E1_Chromosome_11 # 9673 # 9834 # 1 # ID=1_11;partial=00;start_type=GTG;rbs_motif=GGAG/GAGG;rbs_spacer=5-10bp;gc_cont=0.531						
	PROJECT ID	ACCESSION ID	ORGANISMS	CLASS	PROTEIN FUNCTION	PROTEIN ID	%IDENTITY
**Matched Family**	28537	CP001084	Lactobacillus casei str. Zhang, complete genome.	Lactobacillales	hypothetical protein	ADK20034	100.0

**Input Sequence**	ATG-E1_Chromosome_537 # 550479 # 550631 # 1 # ID=1_537;partial=00;start_type=ATG;rbs_motif=AGGAGG;rbs_spacer=5-10bp;gc_cont=0.451						
	PROJECT ID	ACCESSION ID	ORGANISMS	CLASS	PROTEIN FUNCTION	PROTEIN ID	%IDENTITY
**Matched**	402	CP000423	Lactobacillus casei ATCC 334,	Lactobacillales	hypothetical protein	ABJ69248	100.0

Matched Family			complete genome.				

Input Sequence	ATG-E1_Chromosome_2617 # 2657266 # 2657415 # 1 # ID=1_2617;partial=00;start_type=ATG;rbs_motif=GGAG/GAGG;rbs_spacer=5-10bp;gc_cont=0.447						
	PROJECT ID	ACCESSION ID	ORGANISMS	CLASS	PROTEIN FUNCTION	PROTEIN ID	%IDENTITY
Matched Family	28537	CP001084	Lactobacillus casei str. Zhang, complete genome.	Lactobacillales	conserved hypothetical protein	ADK19608	100.0

Input Sequence	ATG-E1_Chromosome_266 # 279232 # 279378 # -1 # ID=1_266;partial=00;start_type=ATG;rbs_motif=AGGAGG;rbs_spacer=5-10bp;gc_cont=0.469						
	PROJECT ID	ACCESSION ID	ORGANISMS	CLASS	PROTEIN FUNCTION	PROTEIN ID	%IDENTITY
Matched Family	28537	CP001084	Lactobacillus casei str. Zhang, complete genome.	Lactobacillales	conserved hypothetical protein	ADK17495	100.0

Input Sequence	ATG-E1_Chromosome_1364 # 1353916 # 1354062 # 1 # ID=1_1364;partial=00;start_type=ATG;rbs_motif=GGAG/GAGG;rbs_spacer=5-10bp;gc_cont=0.381						
	PROJECT ID	ACCESSION ID	ORGANISMS	CLASS	PROTEIN FUNCTION	PROTEIN ID	%IDENTITY
Matched Family	28537	CP001084	Lactobacillus casei str. Zhang, complete genome.	Lactobacillales	conserved hypothetical protein	ADK18366	100.0

Input Sequence	ATG-E1_Chromosome_836 # 856374 # 856517 # 1 # ID=1_836;partial=00;start_type=ATG;rbs_motif=GGAG/GAGG;rbs_spacer=5-10bp;gc_cont=0.424						
	PROJECT ID	ACCESSION ID	ORGANISMS	CLASS	PROTEIN FUNCTION	PROTEIN ID	%IDENTITY
Matched Family	28537	CP001084	Lactobacillus casei str. Zhang, complete genome.	Lactobacillales	conserved hypothetical protein	ADK17905	100.0

Input Sequence	ATG-E1_Chromosome_2596 # 2635233 # 2635352 # -1 # ID=1_2596;partial=00;start_type=ATG;rbs_motif=GGAG/GAGG;rbs_spacer=5-10bp;gc_cont=0.433						
	PROJECT ID	ACCESSION ID	ORGANISMS	CLASS	PROTEIN FUNCTION	PROTEIN ID	%IDENTITY
Matched Family	30359	FM177140	Lactobacillus casei BL23 complete genome, strain BL23.	Lactobacillales	Putative uncharacterized protein	CAQ67648	100.0

Input Sequence	ATG-E1_Chromosome_1201 # 1214583 # 1214723 # -1 # ID=1_1201;partial=00;start_type=ATG;rbs_motif=AGGAGG;rbs_spacer=5-10bp;gc_cont=0.397						
	PROJECT ID	ACCESSION ID	ORGANISMS	CLASS	PROTEIN FUNCTION	PROTEIN ID	%IDENTITY
Matched Family	28537	CP001084	Lactobacillus casei str. Zhang, complete genome.	Lactobacillales	conserved hypothetical protein	ADK18259	100.0

Input Sequence	ATG-E1_Chromosome_2572 # 2616597 # 2616734 # -1 # ID=1_2572;partial=00;start_type=ATG;rbs_motif=AGGAGG;rbs_spacer=5-10bp;gc_cont=0.362						
	PROJECT ID	ACCESSION ID	ORGANISMS	CLASS	PROTEIN FUNCTION	PROTEIN ID	%IDENTITY
Matched Family	402	CP000423	Lactobacillus casei ATCC 334, complete genome.	Lactobacillales	hypothetical protein	ABJ71126	100.0

Input Sequence	ATG-E1_Chromosome_561 # 580130 # 580258 # -1 # ID=1_561;partial=00;start_type=ATG;rbs_motif=AGGA/GGAG/GAGG;rbs_spacer=11-12bp;gc_cont=0.457						
	PROJECT ID	ACCESSION ID	ORGANISMS	CLASS	PROTEIN FUNCTION	PROTEIN ID	%IDENTITY
Matched Family	28537	CP001084	Lactobacillus casei str. Zhang, complete genome.	Lactobacillales	hypothetical protein	ADK17741	100.0

Input Sequence	ATG-E1_Chromosome_2511 # 2550766 # 2550873 # -1 # ID=1_2511;partial=00;start_type=ATG;rbs_motif=AGGA;rbs_spacer=5-10bp;gc_cont=0.398						
	PROJECT ID	ACCESSION ID	ORGANISMS	CLASS	PROTEIN FUNCTION	PROTEIN ID	%IDENTITY
Matched Family	28537	CP001084	Lactobacillus casei str. Zhang, complete genome.	Lactobacillales	conserved hypothetical protein	ADK19493	100.0

Input Sequence	ATG-E1_Chromosome_626 # 655287 # 655379 # 1 # ID=1_626;partial=00;start_type=ATG;rbs_motif=AGxAGG/AGGxGG;rbs_spacer=5-10bp;gc_cont=0.473						
	PROJECT ID	ACCESSION ID	ORGANISMS	CLASS	PROTEIN FUNCTION	PROTEIN ID	%IDENTITY
Matched Family	402	CP000423	Lactobacillus casei ATCC 334, complete genome.	Lactobacillales	hypothetical protein	ABJ69351	100.0

Input Files: *ATG-E1_Chromosome.fasta*

Downloadable files:

RAW MATCHES RESULTS

CITATIONS

For publication of results, please cite:

- PathogenFinder - Distinguishing Friend from Foe Using Bacterial Whole Genome Sequence Data.
  Cosentino S, Voldby Larsen M, Møller Aarestrup F, Lund O
  (2013) PLoS ONE 8(10): e77302.
  PMID: 24204795 doi: 10.1371/journal.pone.0077302

Support Scientific problems Technical problems

Copyright DTU 2011 / All rights reserved
Center for Genomic Epidemiology, DTU, Kemitorvet, Building 204, 2800 Kgs. Lyngby, Denmark
Contact: Vibeke Dybdahl Hammer, Telephone: +45 3588 6420, E-mail: vdha@food.dtu.dk
Funded by: The Danish Council for Strategic Research
Last modified May 22, 2012 11:08:01 GMT
