# Supplementary material for: Lactobacillus paracasei ATG-E1 improves particulate matter 10 plus diesel exhaust particles (PM10D)-induced airway inflammation by regulating immune responses
Source: Front Microbiol. 2023 Apr 27;14:1145546. doi: 10.3389/fmicb.2023.1145546 (PMC10174254; doi:10.3389/fmicb.2023.1145546)
Supplement: Supplementary file 2 [file Data_Sheet_2.pdf]

ResFinder-4.1 Server - Results

Input Files: *ATG-E1_chromosome.fasta*

complete

Antimicrobial	Class	WGS-predicted phenotype	Genetic background
vancomycin	glycopeptide	No resistance	
mupirocin	pseudomonic acid	No resistance	
tobramycin	aminoglycoside	No resistance	
virginiamycin m	streptogramin a	No resistance	
isepamicin	aminoglycoside	No resistance	
virginiamycin s	streptogramin b	No resistance	
hydrogen peroxide	peroxide	No resistance	
butirosin	aminoglycoside	No resistance	
ampicillin	beta-lactam	No resistance	
astromicin	aminoglycoside	No resistance	
lividomycin	aminoglycoside	No resistance	
sulfamethoxazole	folate pathway antagonist	No resistance	
temocillin	beta-lactam	No resistance	
trimethoprim	folate pathway antagonist	No resistance	
oleandomycin	macrolide	No resistance	
florfenicol	amphenicol	No resistance	
fluoroquinolone	quinolone	No resistance	
quinupristin	streptogramin b	No resistance	
fosfomycin	fosfomycin	No resistance	
cephalothin	beta-lactam	No resistance	
lincomycin	lincosamide	No resistance	
butiromycin	aminoglycoside	No resistance	
piperacillin+clavulanic acid	beta-lactam	No resistance	
paromomycin	aminoglycoside	No resistance	
clindamycin	lincosamide	No resistance	
amoxicillin+clavulanic acid	beta-lactam	No resistance	
teicoplanin	glycopeptide	No resistance	
tiamulin	pleuromutilin	No resistance	
ceftiofur	under_development	No resistance	
erythromycin	macrolide	No resistance	
kanamycin	aminoglycoside	No resistance	
gentamicin	aminoglycoside	No resistance	
amikacin	aminoglycoside	No resistance	
tigecycline	tetracycline	No resistance	
ticarcillin+clavulanic acid	beta-lactam	No resistance	
cephalotin	beta-lactam	No resistance	
cefoxitin	beta-lactam	No resistance	

Antimicrobial	Class	WGS-predicted phenotype	Genetic background
hygromycin	aminoglycoside	No resistance	
penicillin	beta-lactam	No resistance	
neomycin	aminoglycoside	No resistance	
ribostamycin	aminoglycoside	No resistance	
dalfopristin	streptogramin a	No resistance	
piperacillin	beta-lactam	No resistance	
telithromycin	macrolide	No resistance	
unknown quinolone	quinolone	No resistance	
amoxicillin	beta-lactam	No resistance	
meropenem	beta-lactam	No resistance	
ethidium bromide	quaternary ammonium compound	No resistance	
sisomicin	aminoglycoside	No resistance	
unknown aminoglycoside	aminoglycoside	No resistance	
cefepime	beta-lactam	No resistance	
spectinomycin	aminocyclitol	No resistance	
doxycycline	tetracycline	No resistance	
piperacillin+tazobactam	beta-lactam	No resistance	
fusidic acid	steroid antibacterial	No resistance	
ciprofloxacin	quinolone	No resistance	
colistin	polymyxin	No resistance	
temperature	heat	No resistance	
imipenem	beta-lactam	No resistance	
arbekacin	aminoglycoside	No resistance	
nalidixic acid	quinolone	No resistance	
metronidazole	nitroimidazole	No resistance	
cefixime	beta-lactam	No resistance	
bleomycin	aminoglycoside	No resistance	
pristinamycin ia	streptogramin b	No resistance	
formaldehyde	aldehyde	No resistance	
tylosin	macrolide	No resistance	
benzylkonium chloride	quaternary ammonium compound	No resistance	
cefotaxime+clavulanic acid	beta-lactam	No resistance	
rifampicin	rifamycin	No resistance	
ceftriaxone	beta-lactam	No resistance	
ceftazidime	beta-lactam	No resistance	
fortimicin	aminoglycoside	No resistance	
carbomycin	macrolide	No resistance	
ticarcillin	beta-lactam	No resistance	
azithromycin	macrolide	No resistance	
chlorhexidine	quaternary ammonium compound	No resistance	
kasugamycin	aminoglycoside	No resistance	
chloramphenicol	amphenicol	No resistance	
cetylpyridinium chloride	quaternary ammonium compound	No resistance	
ampicillin+clavulanic acid	beta-lactam	No resistance	

Antimicrobial	Class	WGS-predicted phenotype	Genetic background
cefotaxime	beta-lactam	No resistance	
quinupristin+dalfopristin	streptogramin a	No resistance	
ceftazidime+avibactam	beta-lactam	No resistance	
apramycin	aminoglycoside	No resistance	
spiramycin	macrolide	No resistance	
dibekacin	aminoglycoside	No resistance	
ertapenem	beta-lactam	No resistance	
tetracycline	tetracycline	No resistance	
linezolid	oxazolidinone	No resistance	
netilmicin	aminoglycoside	No resistance	
minocycline	tetracycline	No resistance	
aztreonam	beta-lactam	No resistance	
unknown beta-lactam	beta-lactam	No resistance	
pristinamycin iia	streptogramin a	No resistance	
streptomycin	aminoglycoside	No resistance	

Download phenotype table (txt) Download species specific phenotype table (txt)

Download acquired AMR gene results:

Results as text Hit in genome sequences Resistance gene sequences Results as tabseperated file

Selected %ID threshold for ResFinder: *90 %*

Selected minimum length for ResFinder: *60 %*

Support Scientific problems Technical problems

Copyright DTU 2011 / All rights reserved
Center for Genomic Epidemiology, DTU, Kemitorvet, Building 204, 2800 Kgs. Lyngby, Denmark
Contact: Vibeke Dybdahl Hammer, Telephone: +45 3588 6420, E-mail: vdha@food.dtu.dk
Funded by: The Danish Council for Strategic Research
Last modified May 22, 2012 11:08:01 GMT
