# Supplementary material for: Lactobacillus paracasei ATG-E1 improves particulate matter 10 plus diesel exhaust particles (PM10D)-induced airway inflammation by regulating immune responses
Source: Front Microbiol. 2023 Apr 27;14:1145546. doi: 10.3389/fmicb.2023.1145546 (PMC10174254; doi:10.3389/fmicb.2023.1145546)
Supplement: Supplementary file 3 [file Data_Sheet_3.docx]

Supplementary Material

*Lactobacillus paracasei* ATG-E1 improves particulate matter 10 plus diesel exhaust particles (PM_10_D)-induced airway inflammation by regulating immune responses

Young-Sil Lee^1, †, *^, Gun-Seok Park^1, †^, Seung-Hyun Ko^1^, Won-Kyung Yang^2^, Hye-Jin Seo^2^, Seung-Hyung Kim^2^, Jihee Kang^1^

*** Correspondence:** Young-Sil Lee: [rheeys04@atogen.co.kr](mailto:rheeys04@atogen.co.kr)

# Supplementary Table 1. Sequences of the primers

#
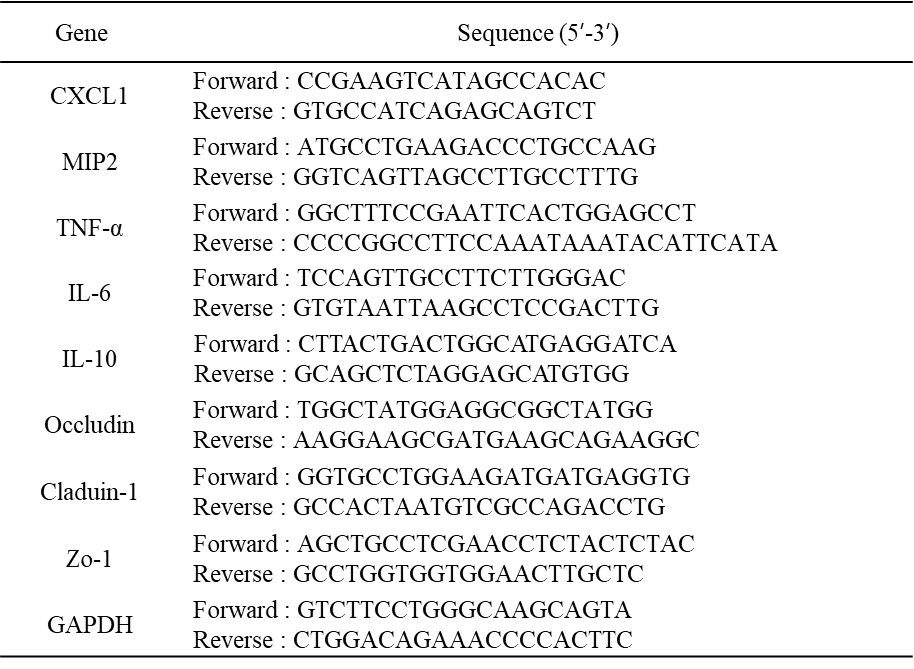


# Supplementary Table 2. Characterization of carbohydrate utilization of *L. paracasei* ATG-E1


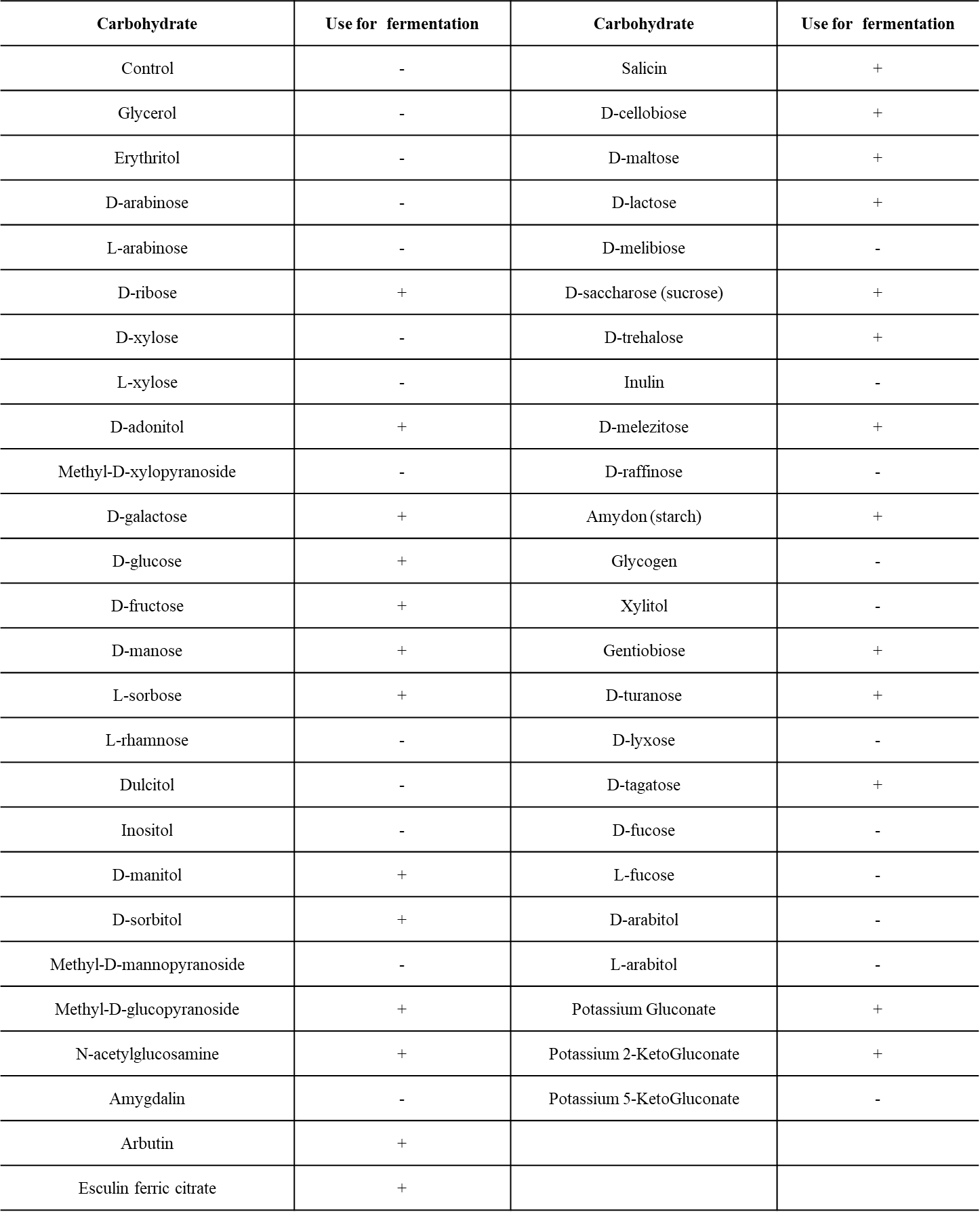


# Supplementary Table 3. Response of *L. paracasei* ATG-E1 to in vitro conditions simulating stomach and duodenum passage (log CFU/ml)


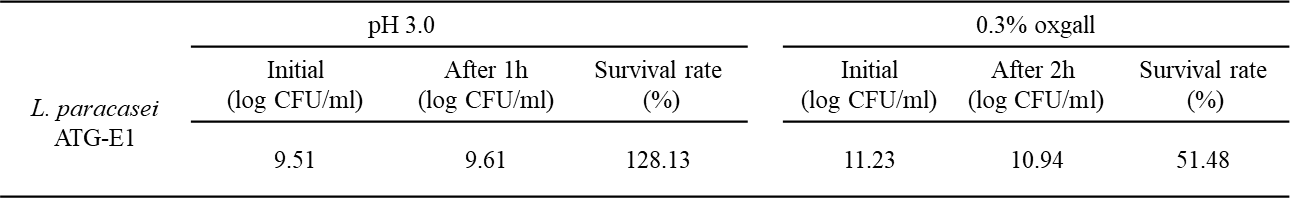


# Supplementary Figure 1. Bile salt hydrolase (BSH) activities


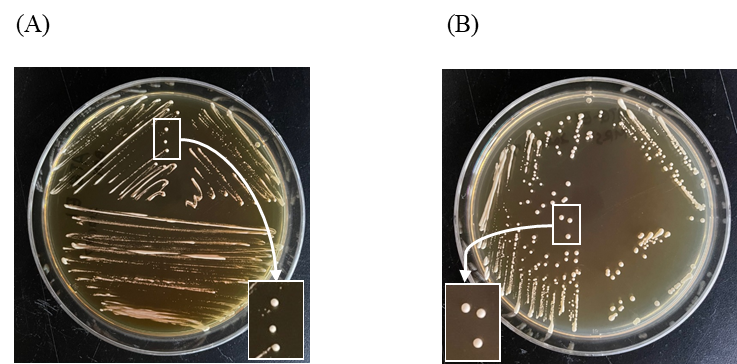


**Supplementary Figure 1.** Bile salt hydrolase (BSH) activities. *L. paracasei* ATG-E1 was streaked on (A) MRS agar plate or (B) MRS agar plates containing 0.5% taurodeoxycholic acid (MRS+TDCA).

# Supplementary Figure 2. Biogenic amine production of *L. paracasei* ATG-E1

#
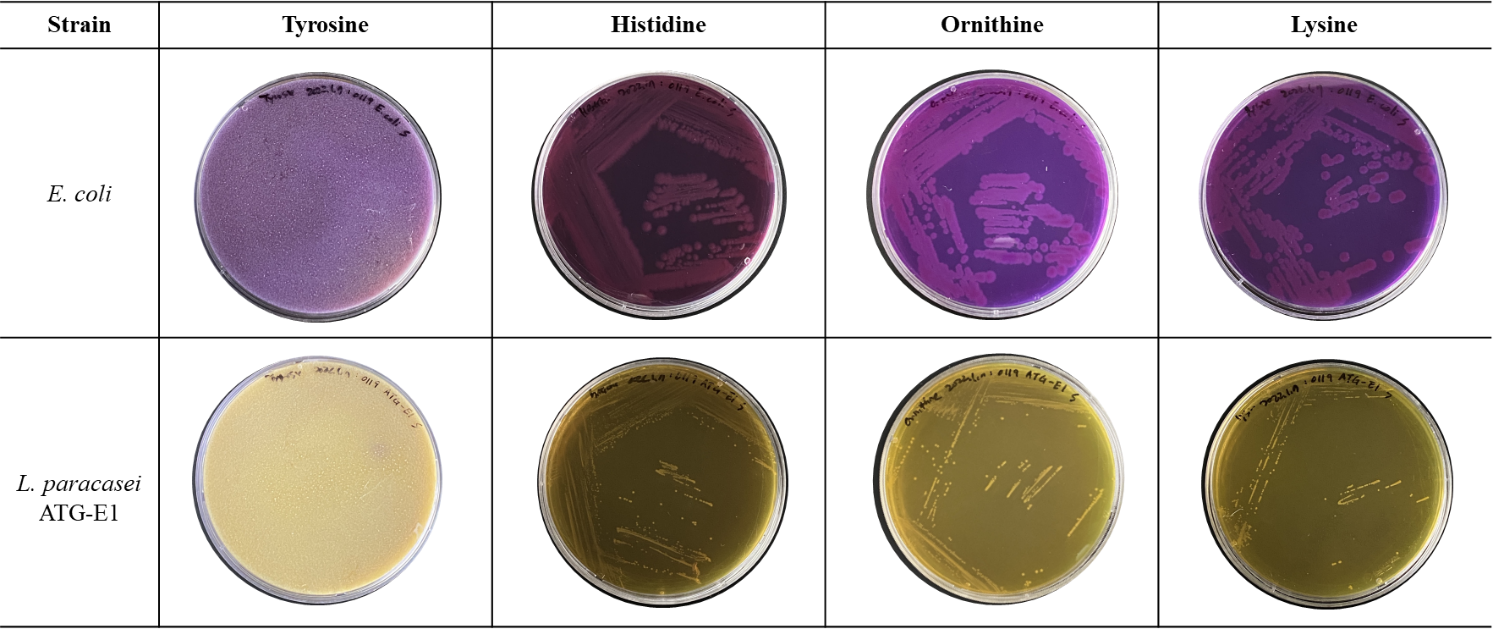


**Supplementary Figure 2.** Biogenic amine production of *L. paracasei* ATG-E1 was cultured in MRS broth containing the precursor amino acids tyrosine, histidine, ornithine, and lysine (Sigma-Aldrich, USA) to detect the production of the biogenic amines tyramine, histamine, putrescine, and cadaverine.

**Supplementary Figure 3.** The effects of *L. paracasei* ATG-E1 on cell subtype analysis in BALF of PM10D-induced airway inflammation.


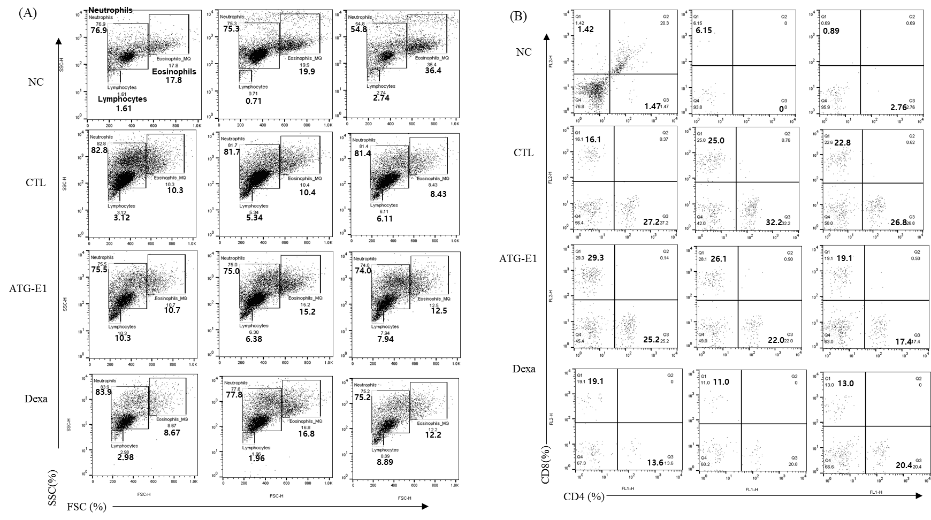


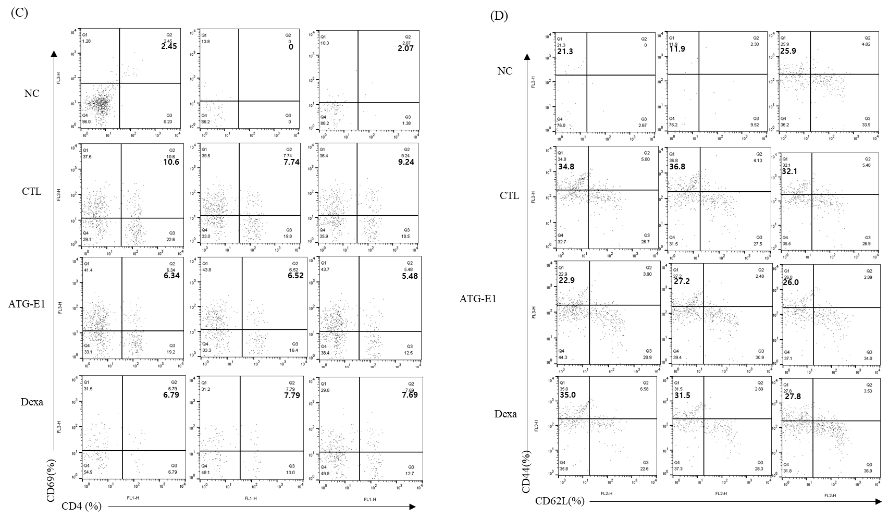


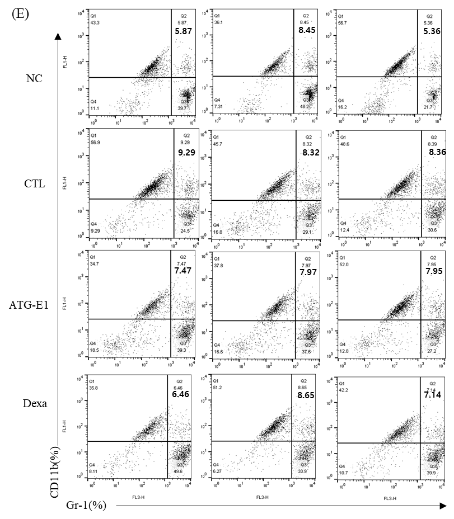


**Supplementary Figure 3.** The effects of *L. paracasei* ATG-E1 on cell subtype analysis in BALF of PM10D-induced airway inflammation. (A) Cell population, (B) CD4^+^ and CD8^+^ cell, (C) CD4^+^CD69^+^ cell, (D) CD62L^-^CD44^high+^ cell, and (E) Gr-1^+^CD11b^+^ cell. NC: BALB/c normal mice; CTL: PM_10_D-sensitized control mice; Dexa: 3 mg/kg dexamethasone-treated PM_10_D-sensitized mice; ATG-E1: 4 × 10^9^ CFU/day of *L. paracasei* ATG-E1-treated PM_10_D-sensitized mice.

**Supplementary Figure 4.** The effects of *L. paracasei* ATG-E1 on cell subtype analysis in lung of PM10D-induced airway inflammation


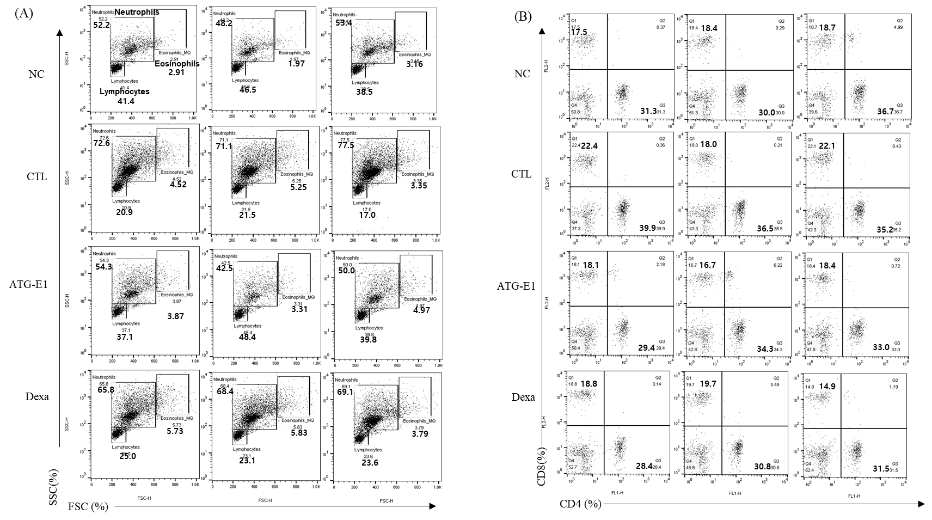


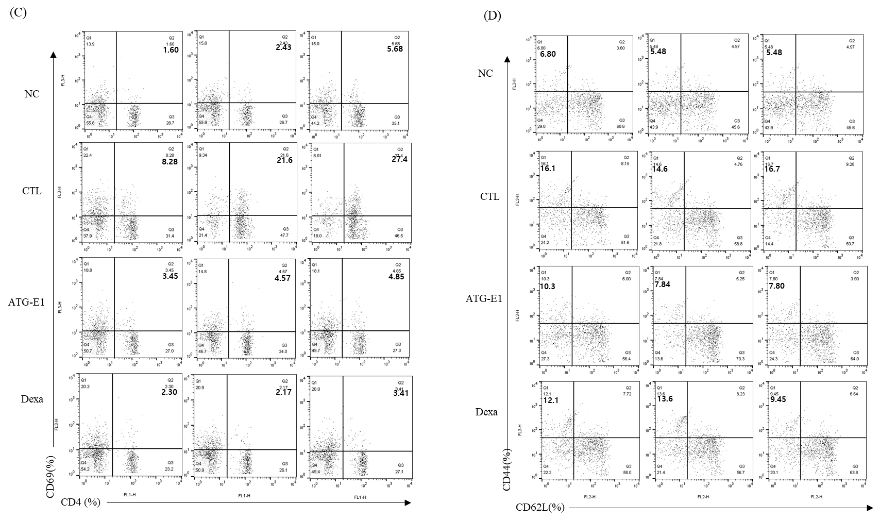


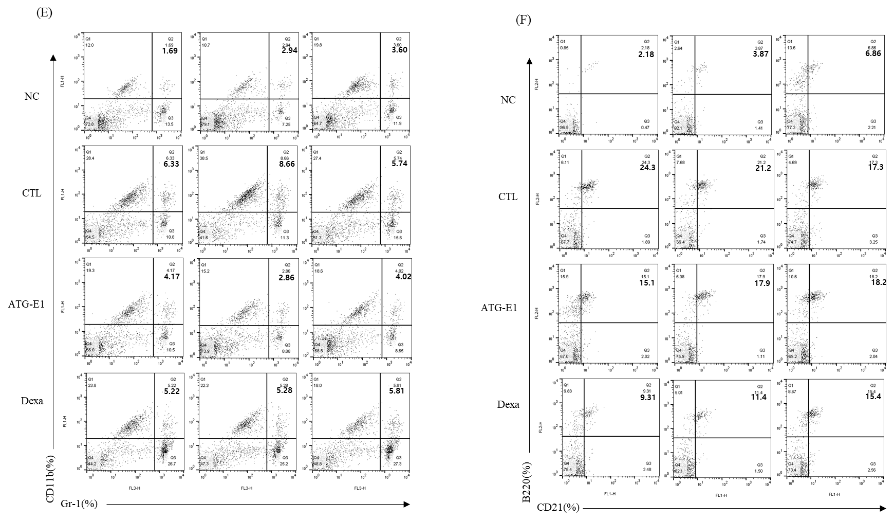


**Supplementary Figure 4.** The effects of *L. paracasei* ATG-E1 on cell subtype analysis in lung of PM10D-induced airway inflammation. (A) Cell population, (B) CD4^+^ and CD8^+^ cell, (C) CD4^+^CD69^+^ cell, (D) CD62L^-^CD44^high+^ cell, (E) Gr-1^+^CD11b^+^cell , and (F) CD21^+^B220^+^ cell. NC: BALB/c normal mice; CTL: PM_10_D-sensitized control mice; Dexa: 3 mg/kg dexamethasone-treated PM_10_D-sensitized mice; ATG-E1: 4 × 10^9^ CFU/day of *L. paracasei* ATG-E1-treated PM_10_D-sensitized mice.

**Supplementary Figure 5.** The effects of *L. paracasei* ATG-E1 on cell subtype analysis in peyer’s patch of PM10D-induced airway inflammation.


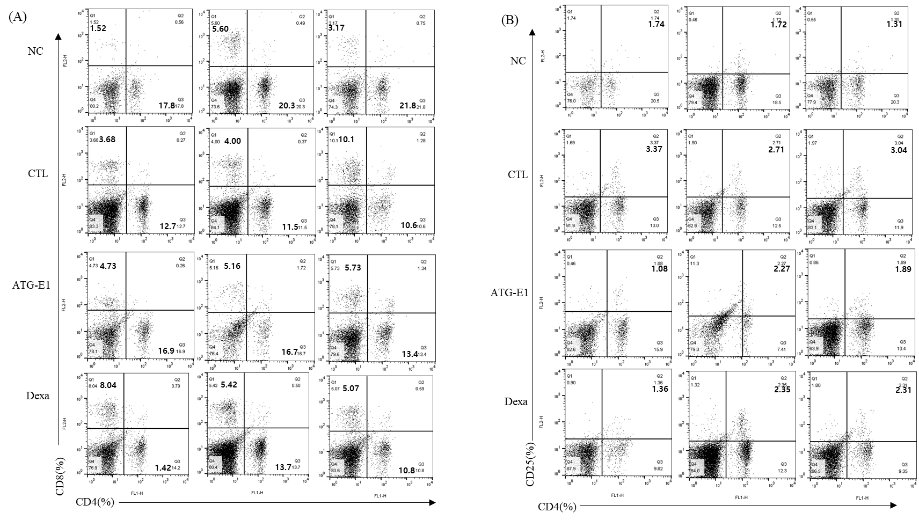


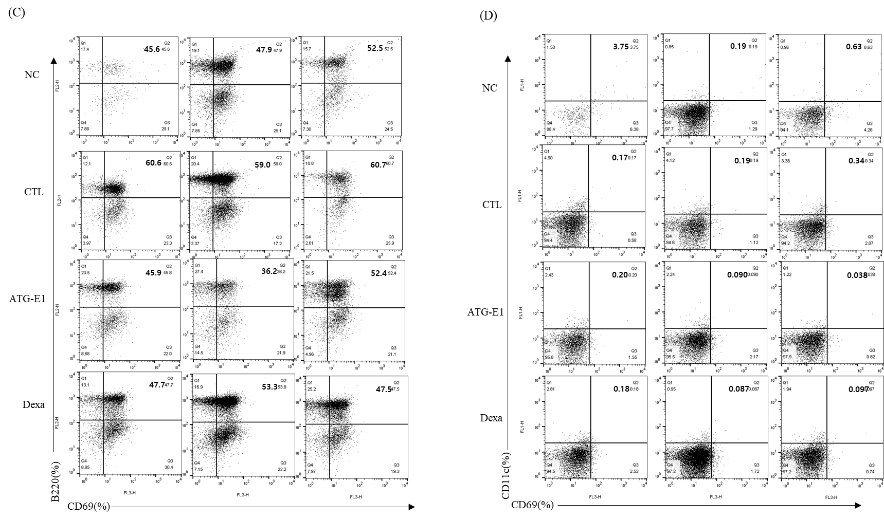


**Supplementary Figure 5.** The effects of *L. paracasei* ATG-E1 on cell subtype analysis in peyer’s patch of PM10D-induced airway inflammation. (A) Cell population, (A) CD4^+^ and CD8^+^ cell, (C) CD4^+^CD25^+^ cell, (D) CD69^+^B220^+^ cell, and (D) CD69^+^CD11c^+^cell. NC: BALB/c normal mice; CTL: PM_10_D-sensitized control mice; Dexa: 3 mg/kg dexamethasone-treated PM_10_D-sensitized mice; ATG-E1: 4 × 10^9^ CFU/day of *L. paracasei* ATG-E1-treated PM_10_D-sensitized mice.
